# Persistent burden and health inequalities of lung cancer among adolescents and young adults, 1990-2021

**DOI:** 10.3389/fonc.2025.1624401

**Published:** 2025-09-30

**Authors:** Jianhui Li, Ju-zi Wang, Qihui Chen, Miaomiao Qin, Jing Zhang, Hui Liu, Yingpin Xun

**Affiliations:** ^1^ Department of Thoracic Surgery, Shanxi Provincial People’s Hospital, Taiyuan, China; ^2^ Nursing Department, Shanxi Provincial People's Hospital, Taiyuan, China

**Keywords:** lung cancer, epidemiology, adolescents and young adults, health inequality, Global Disease Burden 2021

## Abstract

**Background:**

To assess the disease burden, trends, health inequality, and risk factors of lung cancer among adolescents and young adults (AYAs) during the period from 1990 to 2021.

**Methods:**

A secondary analysis was conducted using the Global Burden of Disease (GBD) 2021, focusing on the temporal trends, decomposition analysis, health inequality and risk factors of lung cancer burden among AYAs.

**Results:**

Globally, the rate of lung cancer among AYAs decreased between 1990 and 2021, with the age-standardized incidence rate (ASIR) decreasing from 1.3 (95% uncertainty interval [UI], 1.2 to 1.4) to 0.9 (95% UI, 0.8 to 1.0, AAPC = -1.2), age-standardized mortality rate (ASMR) decreasing from 1.1 (95% UI, 1.0 to 1.2) to 0.7 (95% UI, 0.6 to 0.8, AAPC = -1.4), and age-standardized disability-adjusted life years rate (ASDR) decreasing from 65.4 (95% UI, 60.2 to 71.1) to 42.1 (95% UI, 37.7 to 46.5, AAPC = -1.4). The global number of lung cancer among AYAs has only undergone slight changes, but the middle socio-demographic index (SDI) region, East Asia and China carried heavier lung cancer burden. Notably, the only increase in ASIR, ASDR, and ASMR was found in the low-middle SDI and low SDI regions, especially among women. Decomposition analysis showed that population growth and population aging are the primary driving forces behind the increasing lung cancer burden among AYAs. Smoking was the leading specific risk factor for men and the overall population in 2021, while ambient particulate matter pollution was identified as the leading specific risk for women. Health inequality analysis indicated that the absolute health inequalities marginally declined, while relative health inequalities remained relatively high, and the lung cancer burden among AYAs was predominantly in wealthier countries.

**Conclusions:**

While global ASIR, ASDR, and ASMR of lung cancer among AYAs have declined from 1990 to 2021, cross-national health inequality remained elevated and sustained, particularly in wealthier countries. Increased attention needs to be given to the lung cancer burden among AYAs in low- and middle-income countries and among women, while risk factors such as smoking and ambient particulate matter pollution remain critical targets for intervention.

## 1 Introduction

Adolescents and young adults (AYAs, aged 15 to 39 years) with cancer represent a unique patient group whose biological properties of cancer, timing of cancer diagnosis, and prognostic imaging patterns exhibit significantly different signatures from those of pediatric and older adult patients, making it a focal area in global public health research (1). The National Cancer Institute (NCI) clearly defines AYA cancer as a unique disease entity, characterized by the distinct molecular characteristics, risk factors, diagnoses and treatment strategies, as well as unique psychological, physical, and economic status (2–5). Notably, while AYA cancer survivors face a lower risk of late death, they encounter a higher risk of delayed diagnosis (6).

Presently, the number of AYA population worldwide exceeds 2.9 billion, with the majority residing in low- and middle-income countries (LMICs) (7). According to the International Agency for Research on Cancer (IARC), approximately 1.25 million new cases of AYA cancers and around 0.35 million deaths due to AYA cancers were reported in 2022 (8). Lung cancer, which accounts for 13% of all cancer cases and 23% of all cancer-related deaths worldwide, is the most common cause of cancer-related deaths globally and represents a primary public health problem worldwide (9). Lung cancer is an important part of the AYA cancer patient population, with a particularly significant disease burden. Although significant progress has been made in the field of lung cancer treatment (10), many gaps remain in the understanding of etiology, epidemiology, risk factors, and therapeutic strategies for the AYA lung cancer population. This is largely attributed to the neglect of cancer research specifically focused on the AYA population.

The Global Burden of Disease (GBD) study provides new perspectives to understand disease distribution, trends, and risk factors (11, 12). This study aims to comprehensively reveal the lung cancer burden and health disparities among the AYA population, providing policy implications for policymakers, optimizing the allocation of medical resources, and ultimately alleviating the health inequalities and disease burden related to lung cancer among the global AYA population.

## 2 Methods

### 2.1 Data sources

Our study leveraged data from the 2021 GBD Study. The Bayesian, regularized, and trimmed meta-regression (MR-BRT) was employed by the database to quantify the effect size of each risk-outcome pair. Nonlinear variations in relative risk (RR) functions were captured using through integrated spline functions and adjusted for systematic bias. RR estimates were derived from data obtained through systematic reviews and meta-analyses, with inclusion criteria determined by the 95% uncertainty interval (UI). Additionally, spatio-temporal Gaussian process regression (ST-GPR) and disease model meta-regression (DisMod-MR 2.1) were applied to aggregate heterogeneous data and correct for biases, thus estimating the exposure levels and distributions for each specific risk factor. Lung cancer, which is briefly described as neoplasms located in the trachea, bronchus, or lung, is classified in the International Classification of Diseases (ICD) 10 under the codes C33 and C34-C34.92 (12). The AYA age group for lung cancer includes individuals aged between 15 and 39 years.

In this study, estimates and the 95% UI for incidence, mortality, and disability-adjusted life years (DALYs) lung cancer burden among AYAs were extracted. The dataset from GBD 2021 encompasses global levels, five Socio-Demographic Index (SDI) level regions, 21 GBD regions, and 204 countries and territories from 1990 to 2021. The cancer data were categorized into age cohorts spanning five years each, specifically including aged 15-19, 20-24, 25-29, 30-34, and 35–39 years.

The SDI, developed by GBD researchers, is a composite measure used to assess socio-economic conditions affecting health outcomes across regions. In the GBD 2021 study, SDI values range from 0 to 1, with higher values indicating better socio-economic conditions and improved health outcomes. Based on 2021 SDI, regions are divided into five quintiles: low (0-0.466), low-middle (0.466-0.619), middle (0.619-0.712), high-middle (0.712-0.810), and high (0.810-1) (12).

To evaluate the attributable burden of lung cancer among AYAs associated with risk factors, two metrics (mortality or DALYs) and their corresponding population attributable fractions (PAFs) were assessed and calculated (11). The risk factors chosen, in accordance with the World Cancer Research Fund’s standards of convincing or probable evidence, encompassed smoking, exposure to secondhand smoke, ambient particulate matter pollution, household air pollution from solid fuels, diets deficient in fruits, elevated fasting plasma glucose levels, residential radon, and occupational exposure to substances such as asbestos, arsenic, beryllium, cadmium, chromium, diesel exhaust, nickel, polycyclic aromatic hydrocarbons, and silica. The detailed methodology of the GBD 2021 is presented in a series of key publications. The detailed methods of the GBD 2021 study and the risk assessment involving the lung cancer burden were comprehensively detailed in the collaborator group’s article (11).

### 2.2 Statistics analysis

The estimates were age-standardized to research data using the direct age-standardization. The purpose of age-standardization is to remove the effect of differences in age structure (13). The joinpoint regression model was used to analyze the temporal trends of the age specific rate and age-standardized rate (ASR) from 1990 to 2021. The trend was quantified using the annual percentage change (APC) and the average annual percentage change (AAPC). APC is an indicator used to describe the year-to-year change, while AAPC is an indicator used to describe the average change over a long period of time. The ASRs and numbers for mortality and DALYs were extracted for inequality analysis.

Decomposition analysis was used to identify the drivers of changes in the lung cancer burden among AYAs from 1990 to 2021. The relative contributions of three factors—population growth, aging, and changes in age-specific rate—were evaluated.

According to the World Health Organization Health Equity Assessment guidelines, slope index of inequality (SII) and concentration index serve as two fundamental measures for evaluating absolute and relative income-related inequalities, which are used to assess the health inequalities of disease burden across countries. The SII reflects the absolute difference in the above indicators between the lowest-SDI and highest-SDI countries, with higher absolute values of SII indicated greater inequality. The Concentration Index was calculated using the Lorenz curve based on per capita SDI and the corresponding national burden metrics. It reflects the area between the 45° line and the Lorenz curve. A negative index indicates the burden is higher in low-income countries. Conversely, a positive index indicates a higher burden in high-income countries.

ASR was reported per 100,000 population, with data presented as values with 95% UI. Temporal trends were assessed using joinpoint software (version 5.0.2) from the National Cancer Institute. The values of the AAPC and APC are expressed as percentages, with data presented as values accompanied by 95% confidence intervals (CI). All statistical analyses and mapping were performed using R statistical software (version 4.3.3). A two-sided P value < 0.05 was set as the significance threshold.

## 3 Results

### 3.1 Global trends by sex

The numbers and rates of lung cancer among AYAs are presented in **Supplementary Table 1**. In 2021, there were 28,004 incident cases, 22,622 mortality cases, and 129.1 million DALYs lost due to lung cancer among AYAs worldwide. The age-standardized incidence rate (ASIR), age-standardized mortality rate (ASMR), and age-standardized DALY rate (ASDR) of lung cancer among AYAs were 0.9 cases per 100,000 population, 0.7 cases per 100,000 population, and 42.1 cases per 100,000 population, respectively. With sex-specific classification, male AYAs experienced a higher burden of lung cancer than females. In 1990, the number of incident cases among males was 1.8 times that of females, but this declined to 1.5 times in 2021. Likewise, the ASIR in males was 1.8 times higher than in females in 1990, but decreased to 1.4 times in 2021. Similar patterns were observed for mortality and DALYs. These trends suggest a narrowing gender gap in the lung cancer burden among AYAs over the past three decades.

**Figure 1:**
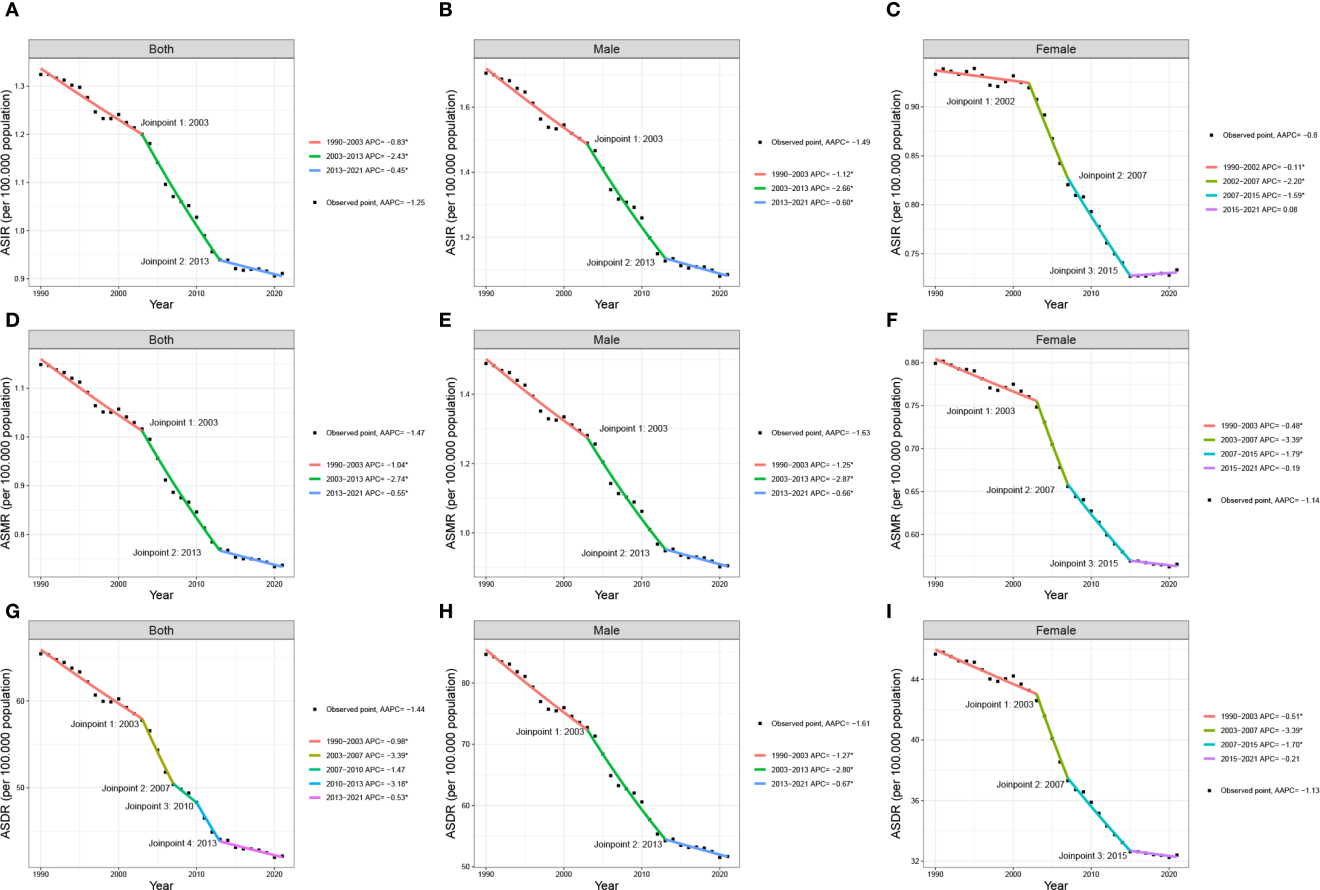
Joinpoint regression analysis in ASIR, ASDR, and ASMR of global lung cancer among AYAs from 1990 to 2021 in both sexes **(A, D, G)**, males **(B, E, H)** and females **(C, F, I)**. AYAs, adolescents and young adults; AAPC, average annual percent change; APC, annual percentage change; DALYs, disability-adjusted life-years; ASIR, age-standardized incidence rate; ASMR, age-standardized mortality rate; ASDR, age-standardized DALYs rate.

The joinpoint regression analysis identified that the ASIR, ASDR, and ASMR of lung cancer among AYAs continued to decrease globally from 1990 to 2021 (ASIR: AAPC = -1.2, 95% CI: -1.3 to -1.1; ASMR: AAPC = -1.4, 95% CI: -1.6 to -1.3; ASDR: AAPC = -1.4, 95% CI: -1.7 to -1.1). The joinpoint regression analysis revealed substantial change points in ASIR in 2003 and 2013. The ASIR decreased between 1990 and 2003 (APC= -0.8, 95% CI: -0.9 to -0.6), decreased at a greater rate between 2003 and 2013 (APC= -2.4, 95% CI: -2.6 to -2.1), and decreased slowly between 2013 and 2021 (APC= -0.4, 95% CI: -0.7 to -0.1). The ASDR and ASMR of lung cancer among AYAs followed the same pattern, that is the decline was larger between 2003 and 2013, but the downward trend became slow between 2013 and 2021. Global trends of lung cancer among AYAs can be found in **Figure 1**. With sex-specific classification, the ASIR with a greater reduction in men than in women (men: AAPC = -1.4, 95% CI: -1.6 to -1.3; women: AAPC = -0.8; 95% CI: -0.9 to -0.7). The trends in the ASDR and ASMR were similar to that of ASIR with respect to sex. Global trends of lung cancer among AYAs by sex can be found in **Figure 1**; **Supplementary Table 1**.

### 3.2 SDI, regional, national trends

Among SDI regions, increases in the ASIR, ASDR, and ASMR were solely observed in the low-middle SDI and low SDI regions. Conversely, regions classified as high SDI, middle SDI, and high-middle SDI exhibited declining trends from 1990 to 2021. Interestingly, in 2021, the high-middle SDI region recorded the highest ASIR, ASDR, and ASMR, with rates of 1.1, 0.9, and 53.6 cases per 100,000 population, respectively. In addition, the middle SDI region had the highest number of incidence, mortality, and DALYs (**Supplementary Table 1**).

At the regional level, Western Sub-Saharan Africa experienced the most significant rise in the ASIR, ASDR, and ASMR from 1990 to 2021 (ASIR: AAPC = 0.53, 95% CI: 0.47 to 0.58; ASMR: AAPC = 0.50, 95% CI: 0.45 to 0.55; ASDR: AAPC = 0.51, 95% CI: 0.46 to 0.56). The largest decrease in the ASIR, ASDR, and ASMR was observed in High-income North America and Central Asia from 1990 to 2021. In the past 32 years, it is worth noting that East Asia was the region with the highest number and ASIR, ASDR, and ASMR from lung cancer among AYAs (**Supplementary Table 1**). Lesotho experienced the most significant increase in the ASIR, ASDR, and ASMR between 1990 and 2021 (ASIR: AAPC = 3.5, 95% CI: 3.0 to 3.9; ASDR: AAPC = 3.5, 95% CI: 3.0 to 3.9; ASMR: AAPC = 3.4, 95% CI: 3.0 to 3.9). Contrariwise, Spain was the highest national rate of decline in the ASDR and ASMR (ASMR: AAPC = -4.5, 95% CI: -5.0 to -4.0; ASDR: AAPC = -4.5, 95% CI: -4.9 to -4.1), while Kazakhstan was the highest decline for the ASIR (AAPC = -4.0, 95% CI: -4.5 to -3.4). In 2021, China recorded the highest number of new cases, deaths, and DALYs, whereas Palau and Monaco had the highest ASDR, ASMR, and ASIR, respectively. (**Supplementary Table 2**).

### 3.3 Decomposition analysis

**Figure 2:**
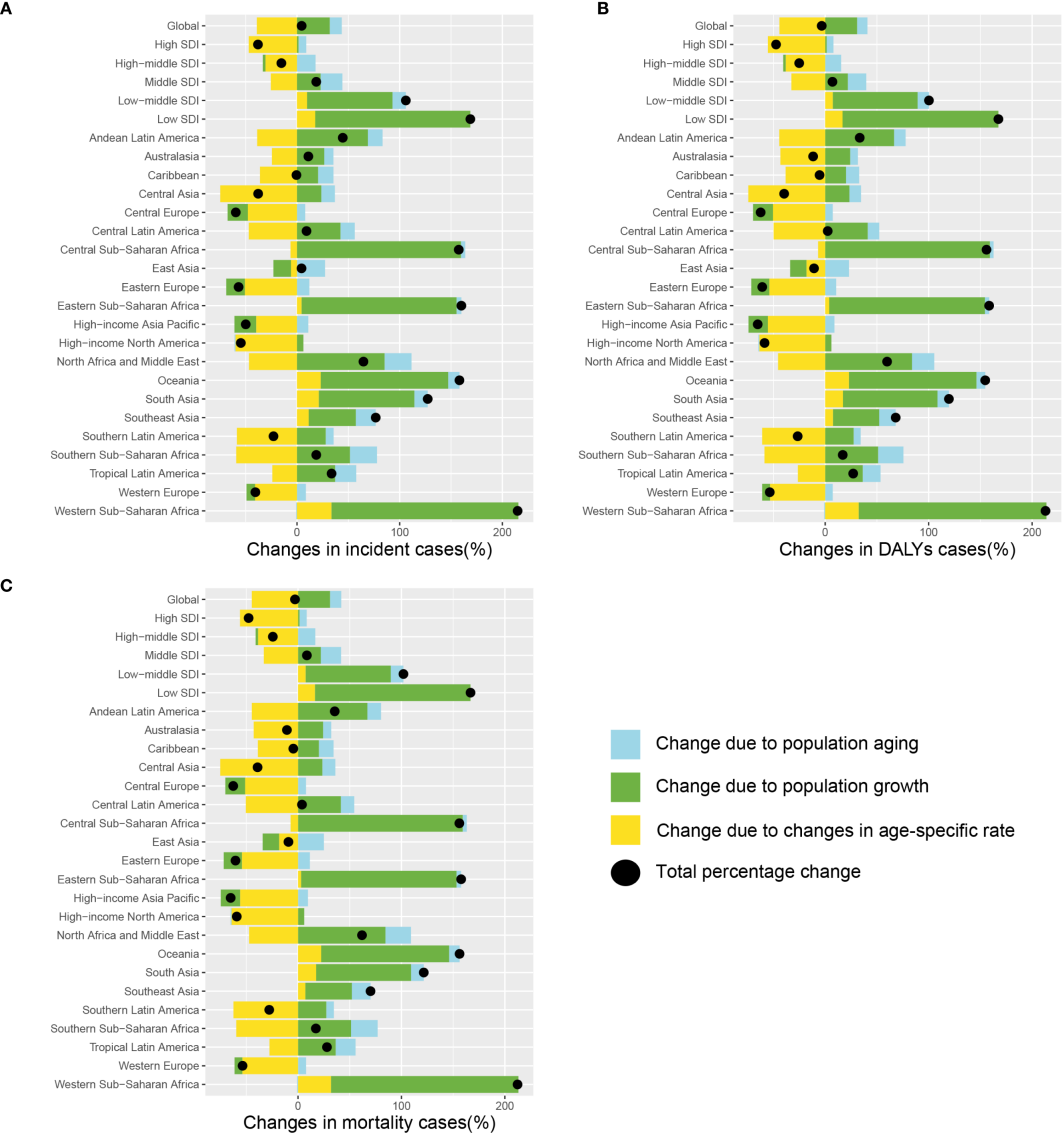
Decomposition analysis in incidence **(A)**, DALYs **(B)**, and mortality **(C)** of the change in lung cancer among AYAs at the global level, SDI level, and regional level, 1990 to 2021. The black dots denote the sum of contribution to the changes in all 3 components. For each component, the magnitude of a positive value indicated a positive contribution; the magnitude of a negative value indicates a negative contribution. Abbreviations: AYAs, adolescents and young adults; DALYs, disability-adjusted life-years; SDI, socio-demographic index.

The changes in the numbers of incident cases, mortality, and DALYs of lung cancer among AYAs from 1990 to 2021 can be attributed to population growth, population ageing, and changes in age-specific rate. During this period, the number of incident cases of lung cancer among AYAs increased from 26,790 in 1990 to 28,004 in 2021, while the number of deaths decreased from 23,309 in 1990 to 22,622 in 2021. Similarly, DALYs decreased from 1,335,868 in 1990 to 1,291,020 in 2021. These shifts provide important demographic context for interpreting the decomposition results. Globally, the overall difference of incident, mortality, and DALYs associated with lung cancer among AYAs have been vanishingly small. Population growth and population aging have contributed to increases, but changes in age-specific rate have led to decreases, which partially offset the overall increase (**Figure 2**). While the overall global changes in case numbers were relatively modest, some regions experienced marked increases, particularly Western Sub-Saharan Africa and low SDI countries. For instance, the number of incident cases with lung cancer among AYAs increased by 4.5%, with 31.8% attributable to population growth, 11.7% from population aging, and 39.0% due to declining age-specific incidence rate. For most of the region, changes in age-specific rate were the primary drivers for the decrease of lung cancer burden among AYAs, whereas population growth was the most significant factor for the increase, as seen in Andean Latin America, Australasia, Central Asia, Central Latin America, Central Sub-Saharan Africa, Southern Latin America, Southern Sub-Saharan Africa. At the regional level, the most significant increasing overall difference of incident cases, mortality, and DALYs in Western Sub-Saharan Africa (Incident cases: 214.9%; Mortality: 212.2%; DALYs: 212.9%). Among the five SDI regions, the most significant increasing changes occurred in the low SDI region (Incident cases: 168.8%; Mortality: 166.7%; DALYs: 167.4%), while the significant decreasing changes was observed in the high SDI region (Incident cases: -38.0%; Mortality: -47.7%; DALYs: -47.4%). Specific numerical values can be found in the **Supplementary Table 3**.

### 3.4 Lung cancer among AYAs health inequality

**Figure 3:**
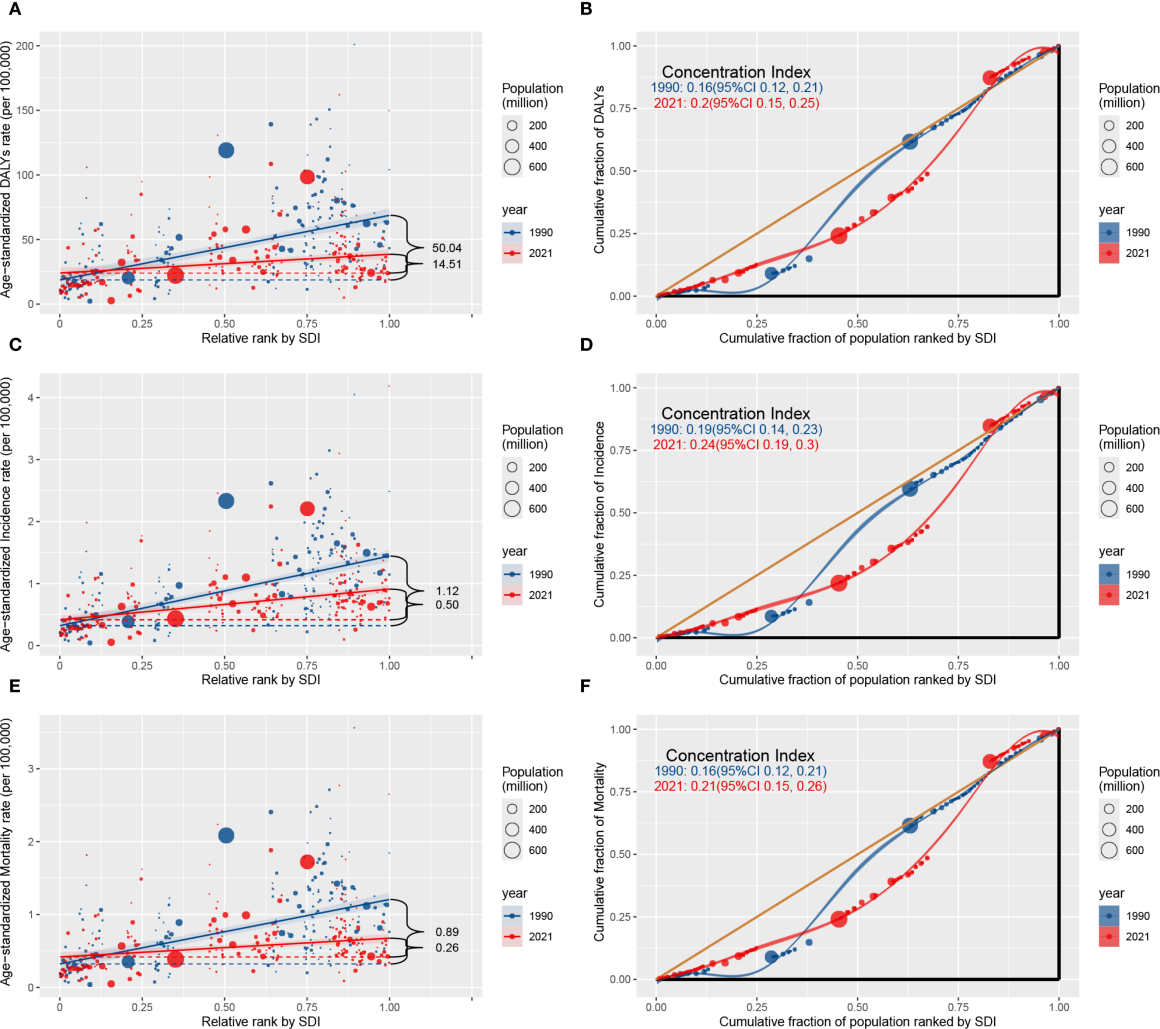
Absolute healthy inequality **(A, C, E)** and relative healthy inequality **(B, D, F)** for ASIR, ASDR, and ASMR of lung cancer among AYAs in both sexes worldwide, 1990 and 2021. The slope index of inequality, shown as the slope of the regression line, represents the absolute difference in lung cancer burden among AYAs between countries or territories with the highest and lowest SDI. The concentration index, calculated as twice the area between the 45° diagonal line and the Lorenz curve, representing the relative extent to which the lung cancer burden among AYAs is concentrated among the poor (negative value) or the rich (positive value). Abbreviations: AYAs, adolescents and young adults; DALYs, disability-adjusted life-years; SDI, Socio-demographic index; ASIR, age-standardized incidence rate; ASMR, age-standardized mortality rate; ASDR, age-standardized DALYs rate.

Significant absolute and relative disparities in the lung cancer burden among AYAs were identified, in the ASIR, ASDR, and ASMR, the slope index has shown a significant decrease over time, whereas the concentration index has exhibited significant increase over the same period (**Figure 3**). From 1990 to 2021, the SII for the ASIR were 1.12 and 0.50; the SII for the ASMR were 0.89 and 0.26; the SII for the ASDR were 50.04 and 14.51, indicating positive correlation between the ASIR, ASDR, and ASMR and SDI. The substantial reduction observed points to a diminishing disparity in the age-standardized health burden of lung cancer among AYAs across the highest SDI and the lowest SDI countries during this period. For example, in 1990, the SII for the ASDR was 50.04, indicating an excess mortality of 50.04 per 100,000 population in the country with the highest SDI compared to the country with the lowest SDI. By 2021, this disparity had decreased to 14.51, reflecting a notable reduction in inequality over time.

By contrast, the analysis of relative inequalities revealed that the concentration indices for the ASIR, ASDR, and ASMR showed consistent upward trends from 1990 and 2021. For example, the concentration index of the ASDR was 0.16 (95% CI 0.12 to 0.21) in 1990 and 0.20 (95% CI 0.15 to 0.25) in 2021. Although the absolute inequality in the burden of lung cancer among AYAs has decreased regionally between high SDI and low SDI countries, inequality remains prevalent. This suggests that the burden of lung cancer among AYAs was excessively focused in wealthier countries. In addition, the decline in the absolute inequality was greater in men than in women, the relative increase in the relative inequality was greater among women than among men (**Supplementary Figures 1**, **2**).

### 3.5 Risk factors of lung cancer burden among AYAs

**Figure 4:**
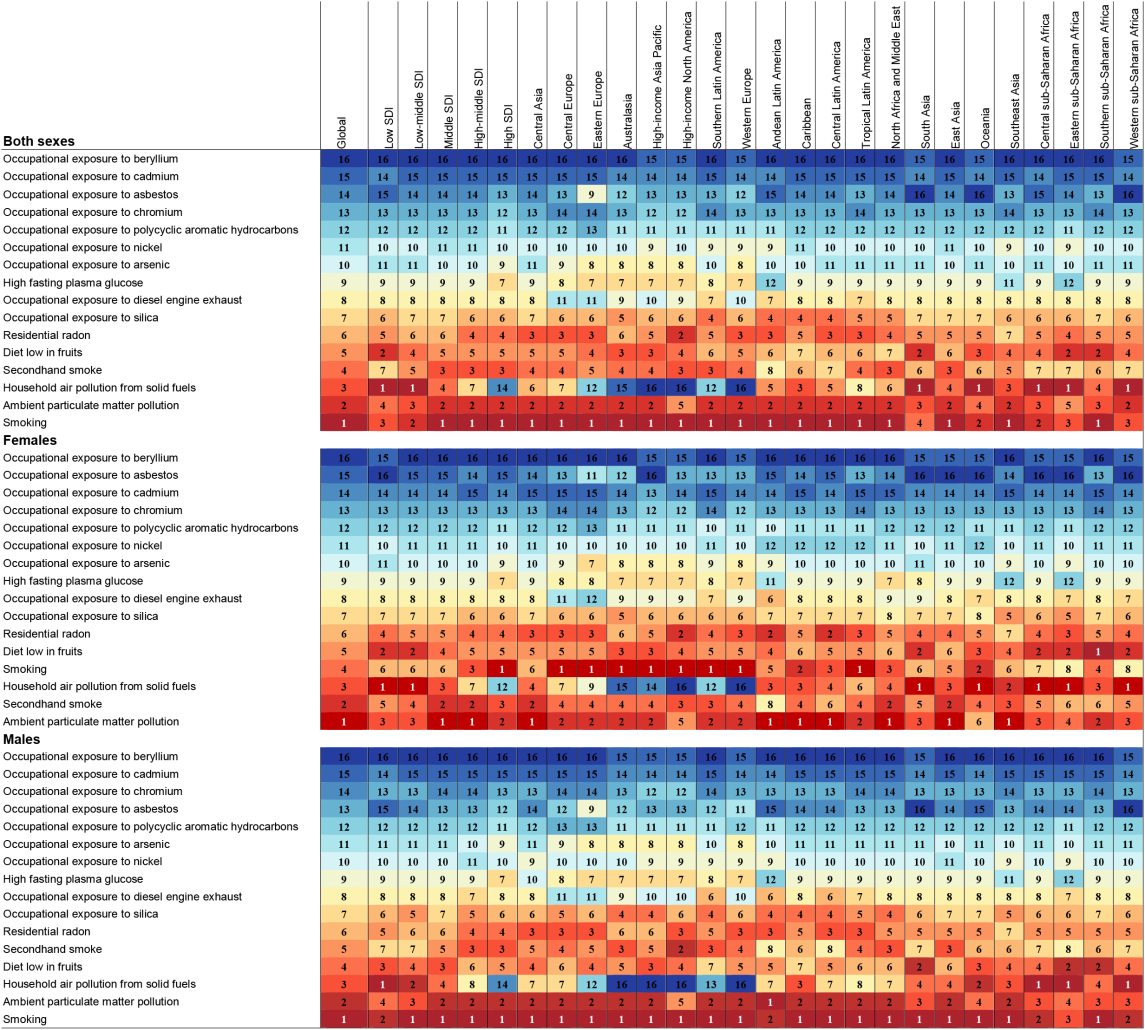
Ranked contribution of risk factors to the ASMR of lung cancer among AYAs by region, 2021, for both sexes, females, and males. Risk factors are ranked from 1 (leading risk factor for ASMR; dark red) to 16 (lowest risk factor for ASMR; dark blue). AYAs, adolescents and young adults; ASMR, age-standardized mortality rate; SDI, socio-demographic index.

**Figure 5:**
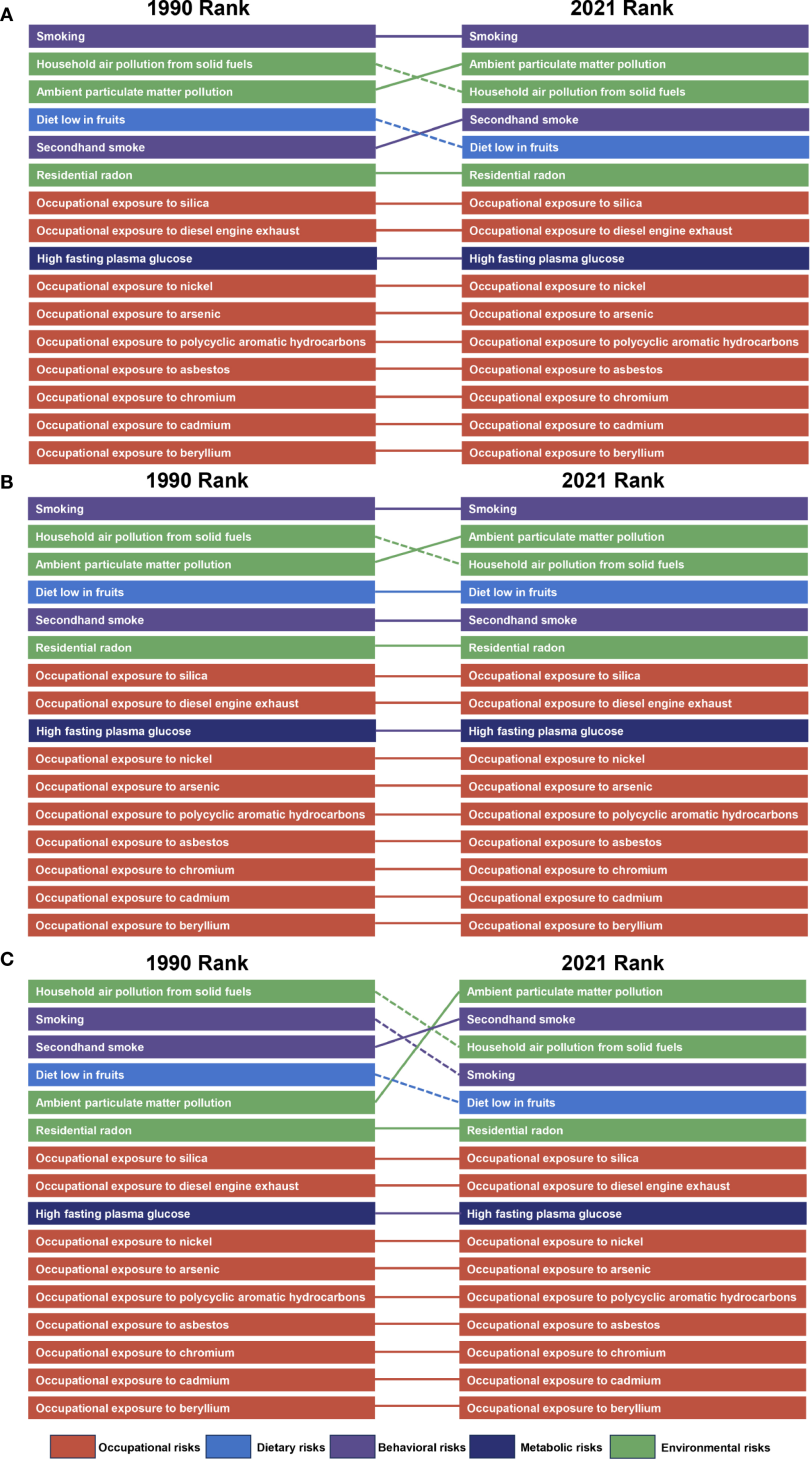
Leading risk factors at the most detailed level for risk-attributable lung cancer among AYAs ASMR globally, 1990-2021. The risk factors for ASMR of lung cancer among AYAs and risk factors moving in or out between 1990 and 2021 are displayed for the global level. Dashed lines indicate decrease in rank. Solid lines indicate increase or no change in rank. From top to bottom: both sexes **(A)**, males **(B)**, and females **(C)**. AYAs, adolescents and young adults; ASMR, age-standardized mortality rate.

In 2021, we assessed the contribution of 16 risk factors to lung cancer burden among AYAs in 21 GBD-defined regions. Globally, smoking, secondhand smoke, household air pollution from solid fuel use, and ambient particulate matter pollution were identified as the main contributor to lung cancer burden among AYAs. The primary risk factors affecting both the general and male populations have exhibited relative stability over recent decades. Nevertheless, the main risk factors in women generally shifted to ambient particulate matter pollution and secondhand smoke, compared to household air pollution from solid fuel use and smoking (**Figure 4**; **Supplementary Figure 3**). In regions characterized by high, high-middle, and middle SDI levels, smoking emerged as the predominant risk factor. Conversely, in low and low-middle SDI regions, the primary risk factor was household air pollution resulting from the use of solid fuels. Ambient particulate matter pollution was the main risk factor in women worldwide. While in men worldwide, the main risk factor remained smoking (**Figure 5**; **Supplementary Figure 4**).Taken together, these findings highlight the varied contributions of risk factors to lung cancer burden among AYAs globally, setting the stage for deeper interpretation in the Discussion section.

## 4 Discussion

This study offers the most comprehensive evaluation of the global lung cancer burden among AYAs, building on the risk factor insights presented above. To the best of our knowledge, it is the first to detail the incidence, mortality, and DALYs, alongside the rates of change in lung cancer among AYAs aged 15–39 years across 204 countries, examined at global, regional, and national levels over this period, thereby filling a gap and providing new insights. Our study revealed that the global lung cancer burden among AYAs in the overall population is on a downward trend. However, the burden has significantly increased among women and in lower and low-middle SDI regions. The lung cancer burden among AYAs is concentrated in wealthier countries. Health inequality, particularly for women, remains severe.

The findings of the global burden of lung cancer among AYAs reveal a complex, evolving phenomenon with significant regional heterogeneity and sex specificity. From 1990 to 2021, in the global AYA population, the numbers of mortality and DALYs due to lung cancer decreased, while the numbers of incidence exhibited a small increase, suggesting that population growth and aging play driving roles while changes in age-specific rate exerted a negative impact according to decomposition analysis. Moreover, the global ASIR, ASDR, and ASMR maintained continuous declines.

In the GBD 2021 study, the observed fluctuation in the burden of lung cancer among AYAs in 2020 and 2021 may reflect changes in healthcare service availability, diagnosis, and reporting, rather than true changes in disease incidence. The examination of data following the COVID-19 pandemic revealed that during the pandemic’s peak in 2020, a temporary decrease in the measured global burden of lung cancer among AYAs was observed, followed by a return to near pre-pandemic levels in 2021. Although this fluctuation was not directly attributed to COVID-19 itself, it highlighted the pandemic’s effect on global systems for lung cancer care. The decrease in the lung cancer burden among AYAs in 2020 was linked to the pressure on healthcare resources, postponements in cancer screenings, and interruptions in treatments during the early stages of the COVID-19 outbreak, leading to under-detection and underreporting of cases (14, 15). Conversely, the rebound observed in 2021 may be partially attributed to the introduction of vaccinations, improvements in epidemic control strategies, and the restoration of healthcare services, resulting in a rise in the measured burden (16).

Our study observed that the lung cancer burden among AYAs demonstrates an overall downward trend, largely attributable to reductions in smoking, secondhand smoke, household air pollution from solid fuel use, and ambient particulate matter pollution. Notably, the lung cancer burden remained greater in men than in women, however, the decline in lung cancer burden was more pronounced in men and the gap in lung cancer burden between men and women has narrowed. However, the relative increase in the burden of lung cancer among women within the AYAs population cannot be solely attributed to smoking. Previous research from the United States has suggested that the higher rates of lung cancer in young women, compared to young men, could not be adequately explained by differences in tobacco use or smoking intensity (17). Although the smoking rate in men is generally higher than in women, the success of smoking cessation campaigns has resulted in a significant increase in the quitting rate among men (18, 19). By contrast, changes in smoking patterns among women have been more limited (20). Our findings demonstrate that, particularly in the high SDI region, Europe, Australia, high-income Asia-Pacific areas, and most of the Americas, smoking has emerged as the leading specific risk factor for lung cancer among young women. Compared to men, women do not demonstrate a significantly enhanced susceptibility to the pulmonary carcinogenic effects of smoking (21, 22). However, within the family environment, exposure to secondhand smoke from cohabiting partners plays a significant role in elevating the risk of lung cancer among women (23). In 2021, secondhand smoke was identified as the second leading risk factor for lung cancer among young women globally. Notably, within China’s lung cancer screening initiatives, young females without a smoking history who receive a lung cancer diagnosis frequently exhibit ground-glass opacity (GGO) nodules in radiology, a specific subtype with a favorable prognosis when detected early (24, 25). Particularly since the onset of the COVID-19 pandemic, a marked rise in the incidental detection of GGO nodules among AYAs has been observed, often during medical visits prompted by typical respiratory complaints such as cough, pneumonia, and breathing difficulties (26). The high incidence of this particular subtype is highlighted, emphasizing the uniqueness and favorable prognosis associated with the lung cancer burden in young women.

When evaluating the impact of household air pollution from solid fuels and environmental particulate matter pollution on young women, regional heterogeneity must be fully accounted. Our research findings indicate that over the past 32 years, substantial progress has been made globally in reducing the lung cancer burden among young women attributable to household air pollution from solid fuels, however environmental particulate matter pollution has increased. In 2021, household air pollution from solid fuels remained the leading risk factor for the lung cancer burden among young women in the low SDI, lower-middle SDI, South Asia, Oceania regions, and in central, eastern, and western sub-Saharan Africa regions. Environmental particulate matter pollution was the first-ranked specific risk factor for the lung cancer burden among young women in the middle SDI, high-middle SDI regions, and the global population. Emissions from the unprocessed biomass fuels contain carcinogens such as benzene and polycyclic aromatic hydrocarbons (PAHs), which are associated with an increased risk of lung cancer (27, 28). In LMICs, due to ethno-cultural and economic status, women’s roles within households result in more frequent exposure to household air pollution from solid fuels, exacerbating the risk of the lung cancer burden in young women (29, 30).

Pollution from environmental particulate matter, particularly ambient fine particles like PM2.5 and PM10, has been identified as a significant contributor to the increasing lung cancer incidence, especially among those who do not smoke (31, 32). In 2016, it was reported that 95% of the global population resided in regions where ambient PM2.5 concentrations surpassed the guidelines set by the World Health Organization (33). In the United States, significant progress in reducing the lung cancer burden linked to environmental particulate matter pollution has been made through the expansion of regulatory measures under the Clean Air Act. While China previously faced a severe lung cancer burden due to particulate matter pollution (34), effective measures, including waste gas emission controls, reduction in coal usage, and adjustments to industrial structures, have led to improvements in public health and a decrease in PM2.5 pollution (35).

The low-middle SDI and low SDI regions showed upward trend in the ASIR, ASDR, and ASMR, with this upward trend being predominantly observed in the female population. In the high SDI regions, the ASIR, ASDR, and ASMR declined at the fastest pace, with this downward trend being most pronounced in the male population. Meanwhile, within the global context, the heaviest disease burden was carried by East Asia and China. The male smoking rate has notably been reduced in the high SDI regions through stringent tobacco control policies. Coupled with heightened health awareness and abundant medical resources, the escalation of the lung cancer burden has been effectively controlled (36). In contrast, little progress has been made in low SDI and lower-middle SDI regions (37). In addition to the impact of various risk factors on the lung cancer burden among AYAs in China and East Asia, the excessive application of LDCT in Asia and China has greatly increased early detection of lung cancer (38, 39), indirectly leading to increased lung cancer burden. This is also one of the key factors contributing to the increasing lung cancer burden in China and the East Asia region (25, 40).

To place the lung cancer burden among AYAs within a broader population context, we further conducted a comparative analysis with older adults aged 60 or 70 years and above. Overall, the lung cancer burden in the older population was significantly higher than that in AYAs, which is consistent with the age-related risk profile of lung cancer. However, the two age groups exhibited distinct patterns in temporal trends and demographic characteristics. From 1990 to 2021, the increase in lung cancer burden was more pronounced in the older population, with the most rapid rises in ASIR, ASMR, and ASDR observed in East Asia and western sub-Saharan Africa, while the most significant declines occurred in high-income North America and central Latin America. In contrast, although the overall burden of lung cancer among AYAs remained lower and generally showed a downward trend, the relative increase in burden among AYA females in low- and lower-middle-SDI regions was more pronounced. This suggests a shifting epidemiological pattern that warrants attention beyond the traditionally high-risk older adult population (41, 42). A comparative analysis of global cancer among AYAs has shown that the incidence rate of cancer in female AYAs is approximately 1.9 times higher than that in their male counterparts. Over the past decade, the incidence of AYAs cancers has increased significantly, whereas mortality rates have declined. Notably, AYAs cancers are characterized by marked health inequalities: incidence rates are substantially higher in high Human Development Index (HDI) countries, while mortality rates tend to be disproportionately higher in countries with lower HDI levels (43). Our findings regarding lung cancer among AYAs partially align with these broader epidemiological patterns. Although the overall burden of lung cancer remains higher in males than in females, we observed an increasing trend in lung cancer burden among AYAs females in low-middle and low-SDI regions. Furthermore, our results also reflect health inequities in AYA lung cancer burden, with incidence and DALY rates concentrated in wealth countries. However, it is important to note that, in contrast to the general rise in AYA cancer incidence, the overall burden of lung cancer among AYAs has shown a downward trend globally.

This study revealed the geographical and socioeconomic distribution of lung cancer burden among AYAs, but lacked optimal comparability for health inequalities. After the introduction of the SII and the Concentration Index, from 1990 to 2021, the lung cancer burden was positively correlated with socio-economic status, with absolute health inequalities slightly decreasing yet relative health inequalities remaining moderately high, particularly in higher SDI regions. The SII indicates absolute health inequality, reflecting the size of disease burden disparities; the Concentration Index demonstrates relative health inequality, showing proportional health differences (44). Absolute inequality declined with disease indicators, whereas relative inequality demonstrated that the lung cancer burden among AYAs concentrated in wealthier countries. Chen et al. proposed that wealthier countries suffer the most severe macroeconomic losses due to cancer, with lung cancer contributing to the largest macroeconomic burden among all types of cancer (45). Furthermore, health inequalities were more pronounced in women, highlighting the critical role of gender in health inequality (46, 47).

### 4.1 Limitations

In this study, firstly, the sources of GBD data largely depends on the availability and quality of GBD 2021 data. The GBD database relies on various data sources worldwide, including hospital records, death registries, censuses, health surveys, and academic research. However, the availability and quality of data vary significantly across countries and regions, particularly in resource-limited countries or regions, where incomplete or low-quality data are particularly prominent issues. This can affect the accuracy of the model’s estimation results. Secondly, the GBD 2021 database did not differentiate between bronchus and lung cancer, resulting in a lack of specific clinical information. Thirdly, the study used manual calculations to determine age-standardized rates for lung cancer among AYA population. While this method removes the impact of variations in age structure, it also restricts the ability to calculate uncertainty intervals. Fourth, the GBD risk attribution model also has certain methodological limitations. The GBD employs a counterfactual comparative risk assessment framework, which assumes that the health effects of individual risk factors are independent. However, in real-world settings, risk factors such as smoking, ambient air pollution, occupational exposures, and secondhand smoke frequently co-occur within the same populations, and may exhibit synergistic or interactive effects. The current model does not fully account for the nonlinear or non-additive impact of multiple simultaneous exposures, which may lead to either overestimation or underestimation of the disease burden attributable to a specific risk factor.

Fifth, we acknowledge that data quality and availability have a substantial impact on the estimation of lung cancer burden, particularly in LMICs, where these issues are especially prominent. Estimates of lung cancer burden may be influenced by missing or poor-quality data, which, in turn, can affect the accuracy of health inequality analyses. Although the GBD framework incorporates various statistical methods to address data sparsity and reporting heterogeneity, these approaches cannot fully eliminate the uncertainty and potential bias introduced by insufficient or low-quality data. One such important source of bias is survivorship bias, which is particularly relevant in populations with relatively low incidence and mortality rates, such as AYAs. In settings with limited resources, constrained case capture, and inadequate follow-up infrastructure, individuals with longer survival times are more likely to be recorded in cancer registries or health databases. In contrast, those with rapidly progressing disease or poor access to care may be systematically underrepresented or missed. Finally, although the GBD database provides a wealth of global and regional health data, there is still insufficient detail in terms of regional granularity and population stratification in some cases. For example, GBD data often focus on the overall disease burden of a country or region, overlooking differences between subpopulations within a country, such as ethnic minorities or specific genders, which prevents the reflection of inequalities at the micro level and leads to insufficiently targeted policy interventions.

## 5 Conclusions

In summary, this study revealed a decline in the global lung cancer burden among AYAs from 1990 to 2021. While higher SDI regions experienced a significant reduction, lower SDI regions exhibited an upward trend. Compared to young men, young women will bear a higher burden of lung cancer in the future. Smoking remains a leading risk factor among the whole population and men worldwide, and ambient particulate matter pollution has become a leading risk factor for young women. Despite advances in surgery and improvements in diagnosis and treatment, the lung cancer burden among AYAs remains concentrated in wealthier countries.

## Data Availability

The datasets presented in this study can be found in online repositories. The names of the repository/repositories and accession number(s) can be found below: The data used in this study came from a public database that everyone can access through the link provided in this article (https://vizhub.healthdata.org/gbd-results/).
