# Supplementary material for: Persistent burden and health inequalities of lung cancer among adolescents and young adults, 1990-2021
Source: Front Oncol. 2025 Sep 30;15:1624401. doi: 10.3389/fonc.2025.1624401 (PMC12518105; doi:10.3389/fonc.2025.1624401)
Supplement: Supplementary file 2 [file DataSheet2.docx]

**Supplemental figure 2.** Absolute healthy inequality (A,C,E) and relative healthy inequality (B,D,F) for ASDR, ASMR, and ASIR of lung cancer among AYAs in females worldwide, 1990 and 2021.


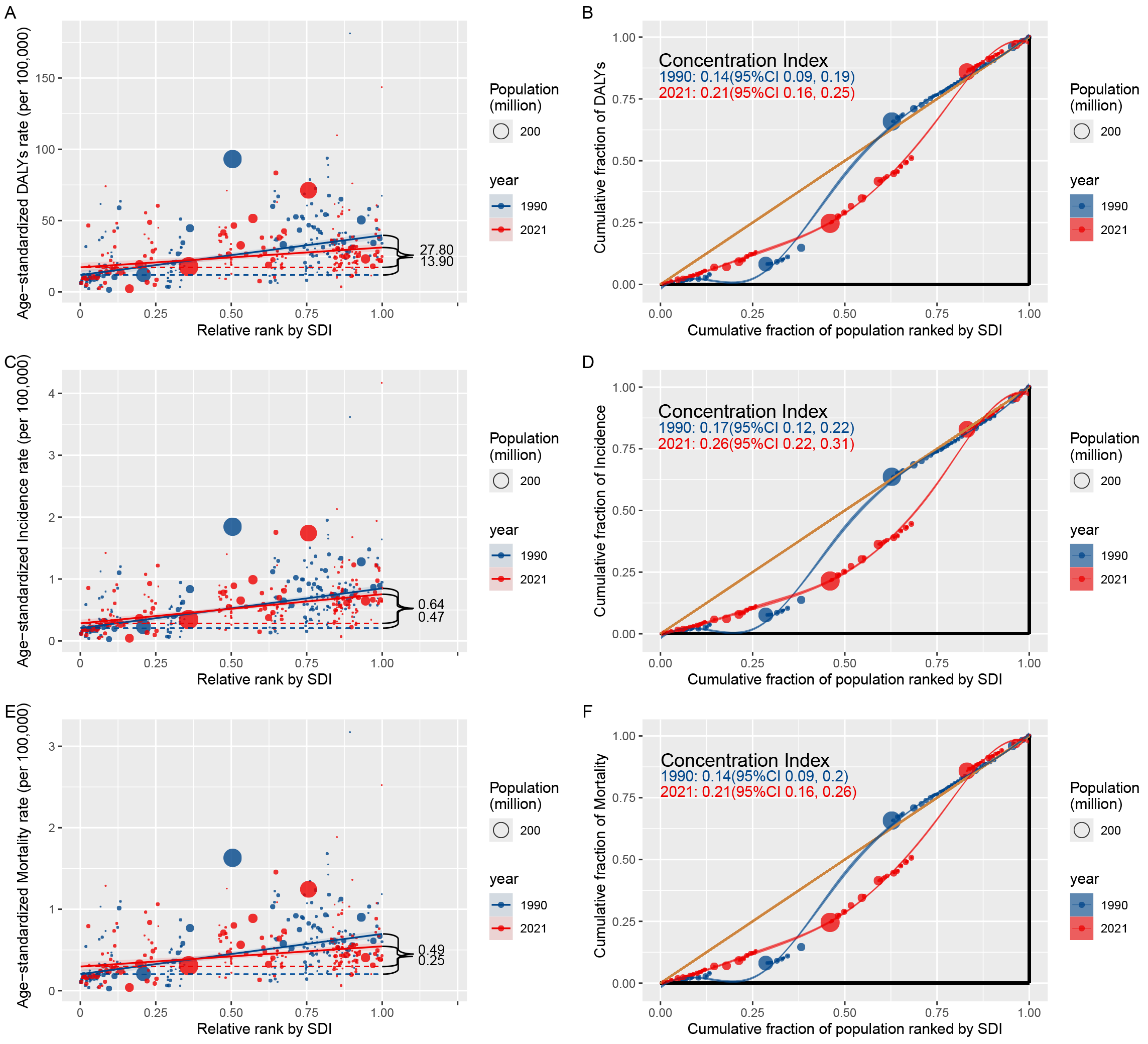
The slope index of inequality, shown as the slope of the regression line, represents the absolute difference in lung cancer burden among AYAs between countries or territories with the highest and lowest SDI. The concentration index, calculated as twice the area between the 45° diagonal line and the Lorenz curve, representing the relative extent to which the lung cancer burden among AYAs is concentrated among the poor (negative value) or the rich (positive value). Abbreviations: AYAs, adolescents and young adults; DALYs, disability-adjusted life-years; ASIR, age-standardized incidence rate; ASMR, age-standardized mortality rate; ASDR, age-standardized DALYs rate. SDI, Socio-demographic index.
