# Supplementary material for: Persistent burden and health inequalities of lung cancer among adolescents and young adults, 1990-2021
Source: Front Oncol. 2025 Sep 30;15:1624401. doi: 10.3389/fonc.2025.1624401 (PMC12518105; doi:10.3389/fonc.2025.1624401)
Supplement: Supplementary file 3 [file DataSheet3.docx]

**Supplemental figure 3**: Leading risk factors at the most detailed level for risk-attributable lung cancer among AYAs ASDR globally, 1990-2021
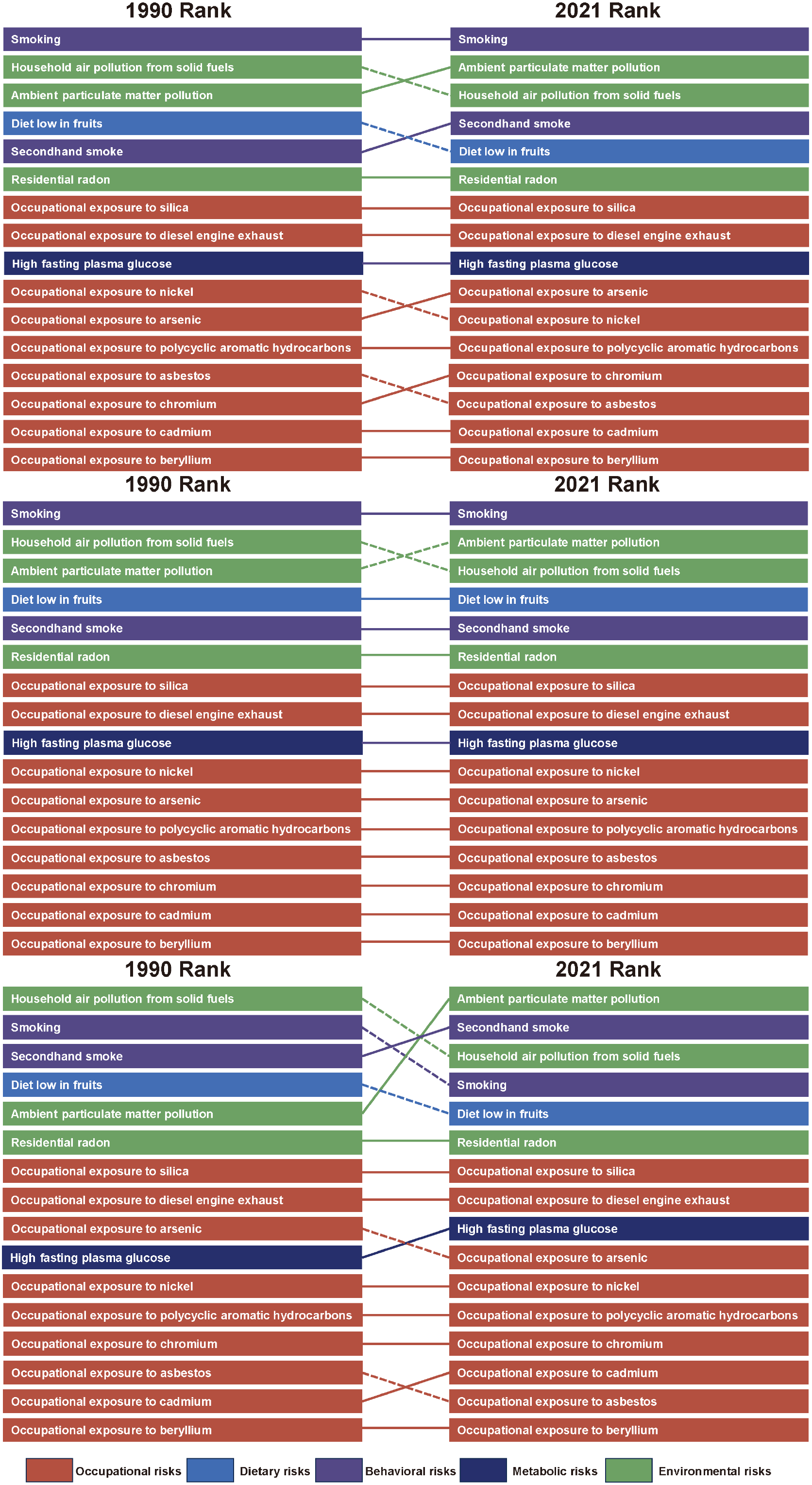


The risk factors for ASDR of lung cancer among AYAs and risk factors moving in or out between 1990 and 2021 are displayed for the global level. Dashed lines indicate decrease in rank. Solid lines indicate increase or no change in rank. From top to bottom: both sexes, males, and females. Abbreviations: AYAs, adolescents and young adults; DALYs, disability-adjusted life-years; ASDR, age-standardized DALYs rate.
