# Supplementary material for: Persistent burden and health inequalities of lung cancer among adolescents and young adults, 1990-2021
Source: Front Oncol. 2025 Sep 30;15:1624401. doi: 10.3389/fonc.2025.1624401 (PMC12518105; doi:10.3389/fonc.2025.1624401)
Supplement: Supplementary file 4 [file DataSheet4.docx]

**Supplemental figure 4**: Ranked contribution of risk factors to the ASDR of lung cancer among AYAs by region, 2021, for both sexes combined, females, and males

| **DALYs**  **Both sexes** | Global | Low SDI | Low-middle SDI | Middle SDI | High-middle SDI | High SDI | Central Asia | Central Europe | Eastern Europe | Australasia | High-income Asia Pacific | High-income North America | Southern Latin America | Western Europe | Andean Latin America | Caribbean | Central Latin America | Tropical Latin America | North Africa and Middle East | South Asia | East Asia | Oceania | Southeast Asia | Central sub-Saharan Africa | Eastern sub-Saharan Africa | Southern sub-Saharan Africa | Western sub-Saharan Africa |
| --- | --- | --- | --- | --- | --- | --- | --- | --- | --- | --- | --- | --- | --- | --- | --- | --- | --- | --- | --- | --- | --- | --- | --- | --- | --- | --- | --- |
| Occupational exposure to beryllium | **16** | **15** | **16** | **16** | **16** | **16** | **16** | **16** | **16** | **16** | **15** | **15** | **16** | **15** | **14** | **16** | **16** | **16** | **16** | **15** | **16** | **15** | **16** | **16** | **16** | **16** | **15** |
| Occupational exposure to cadmium | **15** | **14** | **14** | **15** | **15** | **15** | **15** | **15** | **15** | **14** | **14** | **14** | **15** | **14** | **1** | **15** | **14** | **15** | **15** | **14** | **15** | **14** | **15** | **14** | **14** | **15** | **14** |
| Occupational exposure to asbestos | **14** | **16** | **15** | **14** | **14** | **14** | **14** | **13** | **10** | **12** | **13** | **13** | **13** | **12** | **12** | **14** | **15** | **13** | **14** | **16** | **14** | **16** | **13** | **15** | **15** | **13** | **16** |
| Occupational exposure to chromium | **13** | **13** | **13** | **13** | **13** | **12** | **13** | **14** | **14** | **13** | **12** | **12** | **14** | **13** | **5** | **13** | **13** | **14** | **13** | **13** | **13** | **13** | **14** | **13** | **13** | **14** | **13** |
| Occupational exposure to polycyclic aromatic hydrocarbons | **12** | **12** | **12** | **12** | **12** | **11** | **12** | **12** | **13** | **11** | **11** | **11** | **11** | **11** | **2** | **12** | **12** | **12** | **12** | **12** | **12** | **12** | **12** | **12** | **11** | **12** | **12** |
| Occupational exposure to nickel | **11** | **10** | **10** | **11** | **11** | **10** | **10** | **10** | **9** | **10** | **9** | **10** | **9** | **9** | **4** | **11** | **10** | **10** | **10** | **10** | **11** | **10** | **9** | **10** | **9** | **10** | **10** |
| Occupational exposure to arsenic | **10** | **11** | **11** | **10** | **10** | **9** | **11** | **9** | **8** | **8** | **8** | **8** | **10** | **8** | **11** | **10** | **11** | **11** | **11** | **11** | **10** | **11** | **10** | **11** | **10** | **11** | **11** |
| High fasting plasma glucose | **9** | **9** | **9** | **9** | **9** | **7** | **9** | **8** | **7** | **7** | **7** | **7** | **8** | **7** | **13** | **9** | **9** | **9** | **9** | **9** | **9** | **9** | **11** | **9** | **12** | **9** | **9** |
| Occupational exposure to diesel engine exhaust | **8** | **8** | **8** | **8** | **8** | **8** | **8** | **11** | **12** | **9** | **10** | **9** | **7** | **10** | **3** | **8** | **8** | **7** | **8** | **8** | **8** | **8** | **8** | **8** | **8** | **8** | **8** |
| Occupational exposure to silica | **7** | **7** | **7** | **7** | **6** | **6** | **7** | **6** | **6** | **5** | **6** | **6** | **5** | **6** | **6** | **5** | **4** | **5** | **5** | **7** | **7** | **7** | **6** | **6** | **6** | **7** | **6** |
| Residential radon | **6** | **5** | **6** | **6** | **4** | **4** | **3** | **3** | **3** | **6** | **5** | **2** | **4** | **3** | **9** | **4** | **3** | **3** | **4** | **5** | **5** | **4** | **7** | **5** | **4** | **5** | **5** |
| Diet low in fruits | **5** | **2** | **4** | **5** | **5** | **5** | **5** | **5** | **4** | **3** | **3** | **4** | **6** | **5** | **16** | **7** | **6** | **6** | **7** | **2** | **6** | **3** | **4** | **4** | **2** | **2** | **3** |
| Secondhand smoke | **4** | **6** | **5** | **3** | **3** | **3** | **4** | **4** | **5** | **4** | **4** | **3** | **3** | **4** | **15** | **6** | **7** | **4** | **3** | **6** | **3** | **6** | **5** | **7** | **7** | **6** | **7** |
| Household air pollution from solid fuels | **3** | **1** | **1** | **4** | **7** | **13** | **6** | **7** | **11** | **15** | **16** | **16** | **12** | **16** | **7** | **3** | **5** | **8** | **6** | **1** | **4** | **1** | **3** | **1** | **1** | **4** | **1** |
| Ambient particulate matter pollution | **2** | **4** | **3** | **2** | **2** | **2** | **2** | **2** | **2** | **2** | **2** | **5** | **2** | **2** | **10** | **2** | **1** | **2** | **2** | **3** | **2** | **5** | **2** | **3** | **5** | **3** | **2** |
| Smoking | **1** | **3** | **2** | **1** | **1** | **1** | **1** | **1** | **1** | **1** | **1** | **1** | **1** | **1** | **8** | **1** | **2** | **1** | **1** | **4** | **1** | **2** | **1** | **2** | **3** | **1** | **4** |
| **Females** | | | | | | | | | | | | | | | | | | | | | | | | | | | |
| Occupational exposure to beryllium | **16** | **15** | **16** | **16** | **16** | **16** | **16** | **16** | **16** | **16** | **15** | **15** | **16** | **15** | **16** | **16** | **16** | **16** | **16** | **15** | **15** | **15** | **16** | **15** | **15** | **16** | **15** |
| Occupational exposure to asbestos | **15** | **16** | **15** | **15** | **14** | **15** | **14** | **13** | **11** | **12** | **16** | **13** | **13** | **13** | **15** | **14** | **15** | **13** | **14** | **16** | **16** | **16** | **14** | **16** | **16** | **13** | **16** |
| Occupational exposure to cadmium | **14** | **14** | **14** | **14** | **15** | **14** | **15** | **15** | **15** | **14** | **13** | **14** | **15** | **14** | **14** | **15** | **14** | **15** | **15** | **14** | **14** | **14** | **15** | **14** | **14** | **15** | **14** |
| Occupational exposure to chromium | **13** | **13** | **13** | **13** | **13** | **13** | **13** | **14** | **14** | **13** | **12** | **12** | **14** | **12** | **13** | **13** | **13** | **14** | **13** | **13** | **13** | **13** | **13** | **13** | **13** | **14** | **13** |
| Occupational exposure to polycyclic aromatic hydrocarbons | **12** | **12** | **12** | **12** | **12** | **11** | **12** | **12** | **13** | **11** | **11** | **11** | **10** | **11** | **10** | **11** | **11** | **11** | **12** | **12** | **12** | **11** | **11** | **12** | **11** | **12** | **12** |
| Occupational exposure to nickel | **11** | **10** | **11** | **11** | **11** | **10** | **11** | **10** | **10** | **10** | **10** | **10** | **11** | **10** | **11** | **12** | **12** | **12** | **11** | **10** | **11** | **12** | **10** | **11** | **10** | **11** | **11** |
| Occupational exposure to arsenic | **10** | **11** | **10** | **10** | **10** | **9** | **10** | **9** | **7** | **8** | **8** | **8** | **9** | **8** | **9** | **10** | **10** | **10** | **10** | **11** | **10** | **10** | **9** | **10** | **9** | **10** | **10** |
| High fasting plasma glucose | **9** | **9** | **9** | **9** | **9** | **7** | **9** | **8** | **8** | **7** | **7** | **7** | **8** | **7** | **12** | **9** | **9** | **9** | **7** | **8** | **9** | **9** | **12** | **9** | **12** | **9** | **9** |
| Occupational exposure to diesel engine exhaust | **8** | **8** | **8** | **8** | **8** | **8** | **8** | **11** | **12** | **9** | **9** | **9** | **7** | **9** | **6** | **8** | **8** | **8** | **9** | **9** | **8** | **7** | **8** | **8** | **7** | **8** | **7** |
| Occupational exposure to silica | **7** | **7** | **7** | **7** | **6** | **6** | **7** | **6** | **6** | **5** | **6** | **6** | **6** | **6** | **7** | **7** | **7** | **7** | **8** | **7** | **7** | **8** | **5** | **6** | **5** | **7** | **6** |
| Residential radon | **6** | **4** | **5** | **5** | **4** | **4** | **3** | **3** | **3** | **6** | **5** | **2** | **4** | **3** | **2** | **5** | **2** | **3** | **4** | **4** | **4** | **5** | **6** | **4** | **3** | **5** | **4** |
| Diet low in fruits | **5** | **2** | **2** | **4** | **5** | **5** | **5** | **5** | **5** | **3** | **3** | **4** | **5** | **5** | **4** | **6** | **5** | **5** | **6** | **2** | **6** | **3** | **4** | **2** | **2** | **1** | **2** |
| Smoking | **4** | **6** | **6** | **6** | **3** | **1** | **6** | **1** | **1** | **1** | **1** | **1** | **1** | **1** | **5** | **2** | **3** | **1** | **3** | **6** | **5** | **2** | **7** | **7** | **8** | **4** | **8** |
| Household air pollution from solid fuels | **3** | **1** | **1** | **3** | **7** | **12** | **4** | **7** | **9** | **15** | **14** | **16** | **12** | **16** | **3** | **3** | **4** | **6** | **5** | **1** | **3** | **1** | **2** | **1** | **1** | **3** | **1** |
| Secondhand smoke | **2** | **5** | **4** | **2** | **2** | **3** | **2** | **4** | **4** | **4** | **4** | **3** | **3** | **4** | **8** | **4** | **6** | **4** | **2** | **5** | **2** | **4** | **3** | **5** | **6** | **6** | **5** |
| Ambient particulate matter pollution | **1** | **3** | **3** | **1** | **1** | **2** | **1** | **2** | **2** | **2** | **2** | **5** | **2** | **2** | **1** | **1** | **1** | **2** | **1** | **3** | **1** | **6** | **1** | **3** | **4** | **2** | **3** |
| **Males** | | | | | | | | | | | | | | | | | | | | | | | | | | | |
| Occupational exposure to beryllium | **16** | **16** | **16** | **16** | **16** | **16** | **16** | **16** | **16** | **15** | **15** | **15** | **16** | **15** | **16** | **16** | **16** | **16** | **16** | **15** | **16** | **16** | **16** | **16** | **16** | **16** | **15** |
| Occupational exposure to cadmium | **15** | **14** | **15** | **15** | **15** | **15** | **15** | **15** | **15** | **14** | **14** | **14** | **15** | **14** | **14** | **15** | **15** | **15** | **15** | **14** | **15** | **14** | **15** | **14** | **15** | **15** | **14** |
| Occupational exposure to chromium | **14** | **13** | **13** | **14** | **14** | **13** | **13** | **14** | **14** | **13** | **12** | **12** | **14** | **13** | **13** | **13** | **13** | **14** | **13** | **13** | **13** | **13** | **14** | **13** | **13** | **14** | **13** |
| Occupational exposure to asbestos | **13** | **15** | **14** | **13** | **13** | **12** | **14** | **12** | **10** | **12** | **13** | **13** | **12** | **11** | **15** | **14** | **14** | **13** | **14** | **16** | **14** | **15** | **13** | **15** | **14** | **13** | **16** |
| Occupational exposure to polycyclic aromatic hydrocarbons | **12** | **12** | **12** | **12** | **12** | **11** | **12** | **13** | **13** | **11** | **11** | **11** | **11** | **12** | **11** | **12** | **12** | **12** | **12** | **12** | **12** | **12** | **12** | **12** | **11** | **12** | **12** |
| Occupational exposure to arsenic | **11** | **11** | **11** | **11** | **10** | **9** | **11** | **9** | **8** | **8** | **8** | **8** | **10** | **8** | **10** | **11** | **11** | **11** | **11** | **11** | **10** | **11** | **10** | **11** | **10** | **11** | **11** |
| Occupational exposure to nickel | **10** | **10** | **10** | **10** | **11** | **10** | **9** | **10** | **9** | **10** | **9** | **9** | **9** | **9** | **9** | **10** | **10** | **10** | **10** | **10** | **11** | **10** | **9** | **10** | **9** | **10** | **10** |
| High fasting plasma glucose | **9** | **9** | **9** | **9** | **9** | **7** | **10** | **8** | **7** | **7** | **7** | **7** | **8** | **7** | **12** | **9** | **9** | **9** | **9** | **9** | **9** | **9** | **11** | **9** | **12** | **9** | **9** |
| Occupational exposure to diesel engine exhaust | **8** | **8** | **8** | **8** | **7** | **8** | **8** | **11** | **11** | **9** | **10** | **10** | **6** | **10** | **6** | **8** | **6** | **7** | **8** | **8** | **8** | **8** | **8** | **8** | **7** | **8** | **8** |
| Occupational exposure to silica | **7** | **6** | **5** | **7** | **6** | **6** | **6** | **6** | **6** | **4** | **4** | **6** | **4** | **6** | **4** | **4** | **4** | **5** | **4** | **6** | **7** | **7** | **5** | **6** | **6** | **7** | **6** |
| Residential radon | **6** | **5** | **6** | **6** | **4** | **4** | **3** | **3** | **3** | **6** | **6** | **2** | **5** | **3** | **3** | **5** | **3** | **3** | **5** | **5** | **5** | **4** | **7** | **5** | **5** | **5** | **5** |
| Secondhand smoke | **5** | **7** | **7** | **5** | **3** | **3** | **5** | **4** | **5** | **3** | **5** | **3** | **3** | **4** | **8** | **6** | **8** | **4** | **3** | **7** | **3** | **6** | **6** | **7** | **8** | **6** | **7** |
| Diet low in fruits | **4** | **3** | **4** | **3** | **5** | **5** | **4** | **5** | **4** | **5** | **3** | **4** | **7** | **5** | **5** | **7** | **5** | **6** | **6** | **2** | **6** | **3** | **4** | **4** | **2** | **2** | **4** |
| Household air pollution from solid fuels | **3** | **1** | **2** | **4** | **8** | **14** | **7** | **7** | **12** | **16** | **16** | **16** | **13** | **16** | **7** | **3** | **7** | **8** | **7** | **4** | **4** | **2** | **3** | **1** | **1** | **4** | **1** |
| Ambient particulate matter pollution | **2** | **4** | **3** | **2** | **2** | **2** | **2** | **2** | **2** | **2** | **2** | **5** | **2** | **2** | **1** | **2** | **2** | **2** | **2** | **3** | **2** | **5** | **2** | **3** | **4** | **3** | **3** |
| Smoking | **1** | **2** | **1** | **1** | **1** | **1** | **1** | **1** | **1** | **1** | **1** | **1** | **1** | **1** | **2** | **1** | **1** | **1** | **1** | **1** | **1** | **1** | **1** | **2** | **3** | **1** | **2** |

Notes: Risk factors are ranked from 1 (leading risk factor for ASDR; dark red) to 16 (lowest risk factor for ASDR; dark blue). Abbreviations: AYAs, adolescents and young adults; ASDR, age-standardized DALYs rate; DALYs, disability-adjusted life-years; SDI, socio-demographic index.
