# Supplementary material for: Persistent burden and health inequalities of lung cancer among adolescents and young adults, 1990-2021
Source: Front Oncol. 2025 Sep 30;15:1624401. doi: 10.3389/fonc.2025.1624401 (PMC12518105; doi:10.3389/fonc.2025.1624401)
Supplement: Supplementary file 5 [file Table1.docx]

**Supplemental table 1.****Incident, mortality, and DALYs of** **lung cancer** **among AYAs in 1990 and 2021, and AAPC from 1990 to 2021, by sex, SDI quintile, and region level.**

| **Location** | **Incidence** | | | | | **Mortality** | | | | | **DALYs** | | | | |
| --- | --- | --- | --- | --- | --- | --- | --- | --- | --- | --- | --- | --- | --- | --- | --- |
|  | **incident cases** **(1990)** | **ASIR (1990)** | **incident cases (2021)** | **ASIR (2021)** | **AAPC (1990–2021)** | **Mortality cases (1990)** | **ASMR (1990)** | **Mortality cases (2021)** | **ASMR (2021)** | **AAPC (1990–2021)** | **DALYs cases (1990)** | **ASDR (1990)** | **DALYs cases (2021)** | **ASDR ( 2021)** | **AAPC (1990–2021)** |
| **Global** | 26790(24733 to 29038) | 1.3(1.2 to 1.4) | 28004(25084 to 31021) | 0.9(0.8 to 1.0) | -1.2(-1.3 to -1.1) | 23309(21480to 25335) | 1.1(1.0 to 1.2) | 22622(20240to 24997) | 0.7(0.6 to 0.8) | -1.4(-1.5 to -1.3) | 1335868(1229640 to 1453508) | 65.4(60.2 to 71.1) | 1291020(1156482 to 1426189) | 42.1(37.7 to 46.5) | -1.4(-1.7 to -1.1) |
| **Sex** | | | | | | | | | | | | | | | |
| **Female** | 9329(8184 to 10662) | 0.9(0.8 to 1.0) | 11148(9711 to 12843) | 0.7(0.6 to 0.8) | -0.7(-0.8 to -0.7) | 9329(8184 to 10662) | 0.7(0.6 to 0.9) | 11148(9711 to 12843) | 0.5(0.4 to 0.6) | -1.1(-1.2 to -1.0) | 460977(402666 to 530101) | 45.6(39.9 to 52.4) | 490374(429634 to 561349) | 32.4(28.3 to 37.0) | -1.1(-1.2 to -1.0) |
| **Male** | 17461(15871 to 19413) | 1.7(1.5 to 1.8) | 16855(14520 to 19220) | 1.0(0.9 to 1.2) | -1.4(-1.6 to -1.3) | 17461(15871 to 19413) | 1.4(1.3 to 1.6) | 16855(14520 to 19220) | 0.9(0.7 to 1.0) | -1.6(-1.7 to -1.4) | 874891(792282 to 978409) | 84.6(76.7 to 94.5) | 800646(688215 to 910751) | 51.6(44.4 to 58.7) | -1.6(-1.7 to -1.4) |
| **SDI region** | | | | | | | | | | | | | | | |
| **High SDI** | 5320(5135 to 5512) | 1.4(1.3 to 1.4) | 3298(3111 to 3516) | 0.8(0.7 to 0.8) | -1.8(-2.0 to -1.6) | 4127(3978 to 4284) | 1.1(1.0 to 1.1) | 2154(2030 to 2306) | 0.5(0.4 to 0.5) | -2.3(-2.6 to -2.1) | 231167(222555 to 240189) | 62.6(60.3 to 65.1) | 121386(114370 to 129904) | 29.9(28.2 to 32.0) | -2.3(-2.6 to -2.0) |
| **High-middle SDI** | 9485(8502 to 10536) | 2.1(1.9 to 2.3) | 8042(6774 to 9443) | 1.5(1.2 to 1.7) | -1.0(-1.5 to -0.5) | 8318(7433 to 9274) | 1.8(1.6 to 2.0) | 6290(5303 to 7400) | 1.2(1.0 to 1.4) | -1.4(-1.9 to -0.9) | 474078(423291 to 528989) | 105.9(94.6 to 118.2) | 355050(299369 to 417303) | 68.5(57.8 to 80.4) | -1.4(-1.8 to -0.9) |
| **Middle SDI** | 9461(8395 to 10695) | 1.4(1.2 to 1.6) | 11236(9573 to 12855) | 1.1(0.9 to 1.2) | -0.7(-1.0 to -0.4) | 8545(7583 to 9663) | 1.2(1.1 to 1.4) | 9276(7893 to 10601) | 0.9(0.7 to 1.0) | -1.0(-1.3 to -0.7) | 529921(451039 to 605012) | 73.1(64.9 to 82.7) | 495211(439576 to 560412) | 53.5(45.6 to 61.1) | -1.0(-1.3 to -0.7) |
| **Low-middle SDI** | 2074(1835 to 2372) | 0.5(0.4 to 0.5) | 4272(3781 to 4919) | 0.5(0.4 to 0.6) | 0.2(0 to 0.4) | 1906(1684 to 2181) | 0.4(0.4 to 0.5) | 3849(3401 to 4441) | 0.5(0.4 to 0.5) | 0.1(0 to 0.3) | 223453(197329 to 258034) | 27.5(24.3 to 31.5) | 111565(98525 to 127828) | 28.9(25.5 to 33.4) | 0.1(-0.03 to 0.37) |
| **Low SDI** | 421(347 to 529) | 0.2(0.2 to 0.3) | 1133(924 to 1400) | 0.2(0.2 to 0.3) | 0.3(0 to 0.5) | 387(319 to 486) | 0.2(0.2 to 0.3) | 1034(841 to 1280) | 0.2(0.2 to 0.3) | 0.2(0 to 0.5) | 22525(18557 to 28285) | 14.1(11.7 to 17.7) | 60234(48973 to 74678) | 15.5(12.6 to 19.2) | 0.3(0.06 to 0.54) |
| **Regions** |  |  |  |  |  |  |  |  |  |  |  |  |  |  |  |
| **Andean Latin America** | 135(110 to 168) | 0.9(0.8 to 1.2) | 195(146 to 253) | 0.7(0.5 to 0.9) | -0.9(-1.9 to 0) | 124(101 to 154) | 0.9(0.7 to 1.1) | 168(126 to 217) | 0.6(0.4 to 0.8) | -1.1(-2.1 to -0.2) | 7433(6044 to 9255) | 53.1(43.3 to 66.1) | 9913(7416 to 12769) | 36.8(27.5 to 47.4) | -1.1(-2.0 to -0.2) |
| **Australasia** | 73(63 to 84) | 0.8(0.7 to 0.9) | 81(66 to 99) | 0.6(0.5 to 0.8) | -0.6(-1.2 to 0) | 49(43 to 56) | 0.5(0.5 to 0.6) | 44(37 to 52) | 0.3(0.3 to 0.4) | -1.4(-1.9 to -0.8) | 2800(2447 to 3202) | 33.1(28.9 to 37.8) | 2473(2092 to 2932) | 20.9(17.6 to 24.8) | -1.4(-1.9 to -0.9) |
| **Caribbean** | 139(123 to 157) | 1.0(0.9 to 1.2) | 139(115 to 167) | 0.7(0.6 to 0.9) | -1.2(-1.5 to -0.8) | 119(105 to 133) | 0.9(0.8 to 1.0) | 113(93. to 138) | 0.6(0.5 to 0.7) | -1.3(-1.7 to -1.0) | 6908(6128 to 7757) | 52.4(46.5 to 58.8) | 6533(5348 to 7974) | 35.5(29.1 to 43.3) | -1.2(-1.6 to -0.9) |
| **Central Asia** | 512(476 to 548) | 1.9(1.8 to 2.1) | 318(279 to 363) | 0.7(0.6 to 0.9) | -2.9(-3.2 to -2.5) | 462(429 to 495) | 1.7(1.6 to 1.8) | 281(247 to 322) | 0.7(0.6 to 0.8) | -2.9(-3.4 to -2.4) | 26880(25003 to 28806) | 101.7(94.5 to 109.1) | 16216(14245 to 18620) | 40.7(35.8 to 46.8) | -2.9(-3.3 to -2.4) |
| **Central Europe** | 990(929 to 1059) | 1.8(1.7 to 2.0) | 400(360 to 437) | 0.9(0.8 to 1.0) | -2.2(-2.7 to -1.8) | 859(807 to 919) | 1.6(1.5 to 1.7) | 320(289 to 350) | 0.7(0.6 to 0.8) | -2.5(-3.0 to -1.9) | 47683(44786 to 51052) | 91.9(86.3 to 98.5) | 17937(16176 to 19621) | 42.6(38.4 to 46.6) | -2.4(-3.0 to -1.9) |
| **Central Latin America** | 513(487 to 541) | 0.8(0.8 to 0.9) | 560(491 to 637) | 0.5(0.4 to 0.6) | -1.4(-2.0 to -0.7) | 468(445 to 494) | 0.7(0.7 to 0.8) | 486(425 to 554) | 0.4(0.4 to 0.5) | -1.5(-2.1 to -0.9) | 27858(26471 to 29387) | 45.5(43.3 to 48.1) | 28535(24982 to 32511) | 28.2(24.7 to 32.2) | -1.5(-2.1 to -0.8) |
| **Central Sub-Saharan Africa** | 60(40 to 88) | 0.3(0.2 to 0.5) | 156(102 to 241) | 0.3(0.2 to 0.5) | -0.1(-0.2 to 0) | 55(37 to 81) | 0.3(0.2 to 0.4) | 141(92 to 223) | 0.3(0.2 to 0.4) | -0.1(-0.2 to -0.06) | 3203(2141 to 4683) | 18.4(12.3 to 26.9) | 8196(5315 to 12852) | 17.7(11.5 to 27.8) | -0.1(-0.29 to -0.05) |
| **East Asia** | 12123(10312 to 14100) | 2.3(1.9 to 2.6) | 12657(10174 to 15389) | 2.1(1.7 to 2.6) | -0.1(-0.6 to 0.2) | 10853(9248 to 12634) | 2.0(1.7 to 2.4) | 9844(7885 to 12012) | 1.7(1.3 to 2.0) | -0.6(-1.0 to -0.1) | 623403(530589 to 726243) | 117.9(100.3 to 137.2) | 556118(445835 to 678453) | 97.4(78.0 to 118.8) | -0.6(-1.0 to -0.2) |
| **Eastern Europe** | 1734(1644 to 1841) | 1.8(1.7 to 1.9) | 747(670 to 834) | 0.8(0.7 to 0.9) | -2.3(-3.1 to -1.5) | 1456(1382 to 1540) | 1.5(1.4 to 1.6) | 575(519 to 636) | 0.6(0.6 to 0.7) | -2.6(-3.4 to -1.8) | 81861(77672 to 86605) | 86.1(81.7 to 91.) | 32049(28931 to 35406) | 37.6(33.9 to 41.5) | -2.6(-3.4 to -1.8) |
| **Eastern Sub-Saharan Africa** | 145(121 to 180) | 0.2(0.2 to 0.3) | 379(302 to 486) | 0.2(0.2 to 0.3) | 0.06(-0.03 to 0.16) | 133(111 to 166) | 0.2(0.1 to 0.2) | 344(274 to 444) | 0.2(0.1 to 0.3) | 0.03(-0.05 to 0.13) | 7711(6413 to 9593) | 13.3(11.1 to 16.6) | 19928(15790 to 25768) | 13.5(10.7 to 17.4) | 0.05(-0.04 to 0.15) |
| **High-income Asia Pacific** | 892(814 to 982) | 1.3(1.1 to 1.4) | 447(392 to 517) | 0.7(0.6 to 0.8) | -1.7(-2.1 to -1.3) | 673(610 to 745) | 0.9(0.8 to 1.0) | 234(210 to 264) | 0.3(0.3 to 0.4) | -2.8(-3.3 to -2.3) | 38156(34482 to 42358) | 55.7(50.3 to 61.9) | 13274(11898 to 15002) | 22.7(20.3 to 25.7) | -2.8(-3.3 to -2.3) |
| **High-income North America** | 1860(1800 to 1922) | 1.4(1.4 to 1.5) | 842(804 to 885) | 0.6(0.6 to 0.6) | -2.6(-2.8 to -2.4) | 1388(1345 to 1433) | 1.1(1.0 to 1.1) | 565(541 to 591) | 0.4(0.4 to 0.4) | -2.9(-3.1 to -2.7) | 77412(75049 to 79940) | 62.4(60.5 to 64.4) | 32059(30690 to 33541) | 24.3(23.2 to 25.4) | -2.9(-3.1 to -2.7) |
| **North Africa and Middle East** | 1134(883 to 1435) | 0.9(0.7 to 1.2) | 1867(1591 to 2197) | 0.7(0.5 to 0.8) | -1.0(-1.1 to -0.9) | 1049(817 to 1322) | 0.9(0.7 to 1.1) | 1699(1442 to 2001) | 0.6(0.5 to 0.7) | -1.1(-1.2 to -1.0) | 61776(48149 to 77808) | 52.5(40.8 to 66.0) | 98739(83807 to 116381) | 37.4(31.7 to 44.1) | -1.0(-1.1 to -0.9) |
| **Oceania** | 19(12 to 30) | 0.8(0.5 to 1.2) | 49(32 to 75) | 0.9(0.5 to 1.4) | 0.4(0.3 to 0.6) | 17(11 to 28) | 0.7(0.4 to 1.1) | 45(29 to 70) | 0.8(0.5 to 1.3) | 0.4(0.3 to 0.5) | 1060(683 to 1694) | 43.8(28.4 to 69.4) | 2700(1747 to 4192) | 49.8(32.2 to 77.3) | 0.4(0.3 to 0.5) |
| **South Asia** | 1498(1283 to 1795) | 0.3(0.3 to 0.4) | 3406(2920 to 3994) | 0.4(0.3 to 0.5) | 0.4(0 to 0.7) | 1374(1174 to 1648) | 0.3(0.3 to 0.4) | 3046(2615 to 3577) | 0.3(0.3 to 0.4) | 0.3(-0.02 to 0.69) | 80132(68330 to 96246) | 20.4(17.4 to 24.5) | 175955(150962 to 207431) | 22.8(19.6 to 26.9) | 0.3(-0.02 to 0.67) |
| **Southeast Asia** | 1764(1497 to 2016) | 1.0(0.8 to 1.1) | 3117(2492 to 3795) | 1.0(0.8 to 1.3) | 0.2(0 to 0.4) | 1618(1368 to 1851) | 0.9(0.7 to 1.0) | 2753(2194 to 3351) | 0.9(0.7 to 1.1) | 0.1(-0.1 to 0.3) | 94707(79875 to 108384) | 53.0(44.9 to 60.7) | 159236(126917 to 193894) | 56.0(44.6 to 68.2) | 0.1(-0.1 to 0.3) |
| **Southern Latin America** | 272(235 to 316) | 1.4(1.2 to 1.7) | 210(177 to 248) | 0.7(0.6 to 0.9) | -2.1(-2.6 to -1.5) | 239(206 to 279) | 1.3(1.1 to 1.5) | 172(144 to 203) | 0.6(0.5 to 0.7) | -2.3(-2.8 to -1.7) | 13520(11622 to 15751) | 73.8(63.5 to 86.0) | 9924(8297 to 11745) | 37.2(31.1 to 44.0) | -2.2(-2.7 to -1.6) |
| **Southern Sub-Saharan Africa** | 214(177 to 251) | 1.2(1.0 to 1.4) | 254(212 to 307) | 0.7(0.6 to 0.8) | -1.6(-2.3 to -0.8) | 191(159 to 225) | 1.0(0.9 to 1.2) | 224(187 to 272) | 0.6(0.5 to 0.7) | -1.6(-2.4 to -0.8) | 10803(8986 to 12727) | 60.8(50.5 to 71.7) | 12637(10526 to 15308) | 36.2(30.1 to 43.8) | -1.6(-2.4 to -0.9) |
| **Tropical Latin America** | 476(446 to 509) | 0.8(0.7 to 0.8) | 637(593 to 686) | 0.6(0.6 to 0.7) | -0.6(-1.0 to -0.1) | 430(403 to 460) | 0.7(0.6 to 0.7) | 550(512 to 592) | 0.5(0.5 to 0.6) | -0.7(-1.1 to -0.3) | 24935(23359 to 26698) | 42.3(39.6 to 45.3) | 31684(29507 to 34108) | 33.6(31.3 to 36.2) | -0.7(-1.1 to -0.2) |
| **Western Europe** | 2146(2009 to 2284) | 1.4(1.3 to 1.5) | 1276(1169 to 1401) | 0.8(0.7 to 0.9) | -1.7(-2.1 to -1.2) | 1665(1563 to 1771) | 1.1(1.0 to 1.1) | 771(715 to 834) | 0.5(0.4 to 0.5) | -2.5(-2.8 to -2.3) | 93267(87566 to 99242) | 62.9(59.1 to 67.0) | 43293(40143 to 46855) | 29.5(27.3 to 32.0) | -2.5(-2.8 to -2.3) |
| **Western Sub-Saharan Africa** | 82(66 to 101) | 0.1(0.1 to 0.1) | 258(189 to 344) | 0.1(0.1 to 0.2) | 0.5(0.4 to 0.5) | 75(60 to 93) | 0.1(0.1 to 0.1) | 235(171 to 313) | 0.1(0.1 to 0.1) | 0.5(0.4 to 0.5) | 4349(3500 to 5401) | 7.2(5.8 to 8.9) | 13611(9920 to 18174) | 8.4(6.1 to 11.2) | 0.5(0.4 to 0.5) |

Rates are reported per 100,000 person-years. Data in parentheses are 95% uncertainty intervals for cases and age-standardized rates of incident, mortality, and DALYs, and 95% confidence intervals for AAPCs. Abbreviations: AYAs, adolescents and young adults; DALYs, disability-adjusted life-years; ASIR, age-standardized incidence rate; ASMR, age-standardized mortality rate; ASDR, age-standardized DALYs rate; AAPC, average annual percent change; SDI, socio-demographic index; UI, uncertainty interval.
