# Supplementary material for: Persistent burden and health inequalities of lung cancer among adolescents and young adults, 1990-2021
Source: Front Oncol. 2025 Sep 30;15:1624401. doi: 10.3389/fonc.2025.1624401 (PMC12518105; doi:10.3389/fonc.2025.1624401)
Supplement: Supplementary file 6 [file Table2.docx]

**Supplemental table 2.Incident, mortality, and DALYs of lung cancer among AYAs in 1990 and 2021, and AAPC from 1990 to 2021, by countries.**

| **Location** | **Incidence** | | | | | **Mortality** | | | | | **DALYs** | | | | |
| --- | --- | --- | --- | --- | --- | --- | --- | --- | --- | --- | --- | --- | --- | --- | --- |
|  | **incident cases (1990)** | **ASIR (1990)** | **incident cases (2021)** | **ASIR (2021)** | **AAPC (1990–2021)** | **Mortality cases (1990)** | **ASMR (1990)** | **Mortality cases (2021)** | **ASMR (2021)** | **AAPC (1990–2021)** | **DALYs cases (1990)** | **ASDR (1990)** | **DALYs cases (2021)** | **ASDR ( 2021)** | **AAPC (1990–2021)** |
| **Afghanistan** | 14 (7 to 25 ) | 0.6 (0.3 to 1.0 ) | 77 (45 to 125 ) | 0.8 (0.5 to 1.3 ) | 1.0 (0.8 to 1.2 ) | 13 (6 to 23 ) | 0.6 (0.3 to 1.0 ) | 72 (42 to 116 ) | 0.7 (0.4 to 1.2 ) | 1.0 (0.8 to 1.2 ) | 800 (385 to 1382 ) | 32.3 (15.4 to 56.1 ) | 4368 (2534 to 7037 ) | 43.6 (25.2 to 70.2 ) | 1.0 (0.7 to 1.3 ) |
| **Albania** | 14 (10 to 19 ) | 1.1 (0.8 to 1.5 ) | 10 (6 to 14 ) | 1.0 (0.7 to 1.4 ) | -0.5 (-1.4 to 0.5 ) | 12 (9 to 17 ) | 1.0 (0.7 to 1.3 ) | 8 (5 to 11 ) | 0.8 (0.5 to 1.2 ) | -0.8 (-1.7 to 0.2 ) | 725 (507 to 995 ) | 55.6 (38.9 to 76.2 ) | 446 (292 to 636 ) | 46.6 (30.5 to 66.4 ) | -0.8 (-1.8 to 0.2 ) |
| **Algeria** | 27 (17 to 39 ) | 0.3 (0.2 to 0.5 ) | 48 (30 to 74 ) | 0.2 (0.2 to 0.4 ) | -0.9 (-1.2 to -0.6 ) | 24 (16 to 36 ) | 0.3 (0.2 to 0.4 ) | 43 (27 to 66 ) | 0.2 (0.1 to 0.3 ) | -1.0 (-1.3 to -0.7 ) | 1421 (925 to 2095 ) | 17.3 (11.2 to 25.5 ) | 2409 (1542 to 3721 ) | 12.7 (8.1 to 19.6 ) | -1.0 (-1.3 to -0.7 ) |
| **American Samoa** | 0 (0 to 0 ) | 1.5 (1.0 to 2.2 ) | 0 (0 to 0 ) | 1.6 (1.0 to 2.3 ) | 0.2 (0.0 to 0.5 ) | 0 (0 to 0 ) | 1.3 (0.9 to 2.0 ) | 0 (0 to 0 ) | 1.4 (0.9 to 2.1 ) | 0.2 (-0.1 to 0.4 ) | 15 (10 to 22 ) | 78.9 (51.2 to 116.6 ) | 14 (9 to 20 ) | 83.0 (52.9 to 121.1 ) | 0.2 (-0.1 to 0.4 ) |
| **Andorra** | 0 (0 to 1 ) | 1.3 (0.8 to 2.0 ) | 0 (0 to 0 ) | 0.9 (0.5 to 1.3 ) | -1.4 (-1.9 to -0.9 ) | 0 (0 to 0 ) | 1.0 (0.6 to 1.5 ) | 0 (0 to 0 ) | 0.5 (0.3 to 0.8 ) | -2.0 (-2.5 to -1.5 ) | 15 (9 to 23 ) | 54.4 (34.3 to 84.0 ) | 10 (6 to 15 ) | 30.8 (18.5 to 46.9 ) | -2.0 (-2.4 to -1.5 ) |
| **Angola** | 14 (8 to 22 ) | 0.4 (0.3 to 0.7 ) | 45 (29 to 69 ) | 0.4 (0.3 to 0.7 ) | 0.2 (-0.2 to 0.7 ) | 13 (8 to 20 ) | 0.4 (0.2 to 0.6 ) | 41 (26 to 64 ) | 0.4 (0.3 to 0.6 ) | 0.2 (-0.2 to 0.6 ) | 739 (453 to 1179 ) | 21.9 (13.4 to 34.9 ) | 2392 (1503 to 3712 ) | 23.2 (14.6 to 35.9 ) | 0.2 (-0.2 to 0.7 ) |
| **Antigua and Barbuda** | 0 (0 to 0 ) | 0.5 (0.4 to 0.5 ) | 0 (0 to 0 ) | 0.3 (0.2 to 0.3 ) | -2.0 (-4.6 to 0.6 ) | 0 (0 to 0 ) | 0.4 (0.3 to 0.5 ) | 0 (0 to 0 ) | 0.2 (0.2 to 0.2 ) | -2.2 (-4.7 to 0.4 ) | 5 (5 to 6 ) | 22.1 (19.0 to 25.6 ) | 4 (4 to 5 ) | 11.9 (10.2 to 13.8 ) | -2.1 (-4.6 to 0.4 ) |
| **Argentina** | 205 (171 to 245 ) | 1.7 (1.4 to 2.1 ) | 146 (119 to 177 ) | 0.8 (0.7 to 1.0 ) | -2.5 (-3.1 to -1.8 ) | 180 (150 to 215 ) | 1.5 (1.3 to 1.8 ) | 122 (100 to 148 ) | 0.7 (0.6 to 0.8 ) | -2.6 (-3.3 to -2.0 ) | 10116 (8450 to 12121 ) | 84.8 (70.9 to 101.7 ) | 7018 (5715 to 8517 ) | 39.0 (31.7 to 47.4 ) | -2.6 (-3.2 to -2.0 ) |
| **Armenia** | 38 (35 to 42 ) | 2.7 (2.5 to 2.9 ) | 11 (10 to 13 ) | 0.9 (0.8 to 1.0 ) | -3.5 (-4.6 to -2.4 ) | 34 (32 to 37 ) | 2.4 (2.2 to 2.6 ) | 10 (9 to 11 ) | 0.8 (0.7 to 0.9 ) | -3.6 (-4.7 to -2.5 ) | 1992 (1825 to 2161 ) | 139.0 (127.4 to 150.6 ) | 552 (488 to 620 ) | 44.8 (39.6 to 50.3 ) | -3.5 (-4.6 to -2.5 ) |
| **Australia** | 63 (53 to 74 ) | 0.9 (0.8 to 1.0 ) | 68 (54 to 85 ) | 0.7 (0.5 to 0.9 ) | -0.7 (-1.6 to 0.3 ) | 41 (35 to 48 ) | 0.6 (0.5 to 0.7 ) | 35 (29 to 43 ) | 0.4 (0.3 to 0.4 ) | -1.5 (-2.4 to -0.6 ) | 2332 (1980 to 2734 ) | 33.0 (28.0 to 38.7 ) | 1973 (1627 to 2390 ) | 19.9 (16.4 to 24.2 ) | -1.5 (-2.4 to -0.7 ) |
| **Austria** | 35 (29 to 42 ) | 1.2 (1.0 to 1.4 ) | 23 (18 to 29 ) | 0.7 (0.6 to 0.9 ) | -1.6 (-2.5 to -0.6 ) | 27 (22 to 32 ) | 0.9 (0.7 to 1.1 ) | 13 (11 to 16 ) | 0.4 (0.3 to 0.5 ) | -2.4 (-3.5 to -1.3 ) | 1495 (1231 to 1801 ) | 50.4 (41.6 to 60.6 ) | 760 (610 to 938 ) | 23.9 (19.2 to 29.5 ) | -2.4 (-3.5 to -1.3 ) |
| **Azerbaijan** | 54 (43 to 67 ) | 1.9 (1.5 to 2.3 ) | 47 (32 to 66 ) | 1.0 (0.7 to 1.4 ) | -2.1 (-3.2 to -1.0 ) | 49 (39 to 61 ) | 1.7 (1.4 to 2.1 ) | 42 (28 to 58 ) | 0.9 (0.6 to 1.2 ) | -2.2 (-3.2 to -1.1 ) | 2880 (2292 to 3558 ) | 99.0 (78.4 to 122.7 ) | 2381 (1634 to 3285 ) | 50.5 (34.8 to 69.4 ) | -2.3 (-3.3 to -1.2 ) |
| **Bahamas** | 1 (1 to 1 ) | 0.9 (0.8 to 1.0 ) | 2 (1 to 2 ) | 1.0 (0.8 to 1.3 ) | 0.5 (-0.2 to 1.2 ) | 1 (1 to 1 ) | 0.8 (0.7 to 0.9 ) | 1 (1 to 2 ) | 0.9 (0.7 to 1.1 ) | 0.3 (-0.4 to 1.1 ) | 47 (41 to 53 ) | 43.9 (38.2 to 50.2 ) | 76 (59 to 99 ) | 48.8 (37.5 to 63.1 ) | 0.3 (-0.4 to 1.0 ) |
| **Bahrain** | 3 (2 to 3 ) | 0.9 (0.7 to 1.3 ) | 6 (4 to 9 ) | 0.8 (0.5 to 1.1 ) | -0.5 (-1.0 to 0.0 ) | 2 (2 to 3 ) | 0.9 (0.6 to 1.2 ) | 6 (4 to 8 ) | 0.7 (0.5 to 1.0 ) | -0.6 (-1.1 to -0.1 ) | 136 (97 to 186 ) | 50.6 (35.9 to 69.3 ) | 325 (220 to 469 ) | 41.9 (28.4 to 60.2 ) | -0.6 (-1.1 to -0.1 ) |
| **Bangladesh** | 119 (72 to 205 ) | 0.3 (0.2 to 0.6 ) | 219 (131 to 398 ) | 0.3 (0.2 to 0.6 ) | 0.0 (-0.4 to 0.4 ) | 110 (66 to 189 ) | 0.3 (0.2 to 0.5 ) | 196 (116 to 358 ) | 0.3 (0.2 to 0.5 ) | -0.1 (-0.5 to 0.4 ) | 6469 (3861 to 11067 ) | 17.9 (10.7 to 30.7 ) | 11397 (6754 to 20862 ) | 17.0 (10.1 to 31.1 ) | -0.1 (-0.5 to 0.4 ) |
| **Barbados** | 1 (0 to 1 ) | 0.5 (0.4 to 0.6 ) | 0 (0 to 1 ) | 0.4 (0.3 to 0.5 ) | -0.6 (-1.6 to 0.3 ) | 0 (0 to 1 ) | 0.4 (0.4 to 0.5 ) | 0 (0 to 0 ) | 0.3 (0.3 to 0.4 ) | -0.9 (-1.7 to 0.0 ) | 27 (23 to 30 ) | 24.4 (21.4 to 27.7 ) | 20 (15 to 25 ) | 18.7 (14.5 to 24.2 ) | -0.9 (-1.7 to 0.0 ) |
| **Belarus** | 59 (48 to 73 ) | 1.4 (1.1 to 1.7 ) | 28 (20 to 36 ) | 0.7 (0.5 to 0.9 ) | -2.4 (-4.0 to -0.8 ) | 51 (41 to 62 ) | 1.2 (1.0 to 1.5 ) | 20 (15 to 27 ) | 0.5 (0.4 to 0.7 ) | -2.9 (-4.5 to -1.3 ) | 2832 (2292 to 3455 ) | 67.2 (54.4 to 82.0 ) | 1120 (831 to 1473 ) | 29.5 (21.8 to 38.8 ) | -2.9 (-4.5 to -1.3 ) |
| **Belgium** | 69 (57 to 82 ) | 1.7 (1.4 to 2.1 ) | 40 (33 to 49 ) | 1.0 (0.8 to 1.3 ) | -2.0 (-2.7 to -1.3 ) | 54 (45 to 64 ) | 1.4 (1.1 to 1.6 ) | 26 (22 to 31 ) | 0.7 (0.5 to 0.8 ) | -2.6 (-3.2 to -1.9 ) | 3023 (2495 to 3585 ) | 76.0 (62.7 to 90.2 ) | 1470 (1210 to 1767 ) | 37.6 (30.9 to 45.3 ) | -2.5 (-3.2 to -1.9 ) |
| **Belize** | 0 (0 to 0 ) | 0.4 (0.4 to 0.5 ) | 1 (1 to 1 ) | 0.6 (0.5 to 0.7 ) | 0.8 (-0.4 to 2.0 ) | 0 (0 to 0 ) | 0.4 (0.3 to 0.4 ) | 1 (1 to 1 ) | 0.5 (0.4 to 0.6 ) | 0.7 (-0.5 to 1.8 ) | 14 (12 to 16 ) | 22.8 (20.0 to 26.0 ) | 52 (43 to 61 ) | 29.5 (24.5 to 35.1 ) | 0.6 (-0.5 to 1.7 ) |
| **Benin** | 3 (2 to 5 ) | 0.2 (0.1 to 0.3 ) | 12 (7 to 19 ) | 0.3 (0.2 to 0.4 ) | 0.6 (0.4 to 0.7 ) | 3 (2 to 4 ) | 0.2 (0.1 to 0.3 ) | 11 (6 to 18 ) | 0.2 (0.1 to 0.4 ) | 0.6 (0.4 to 0.7 ) | 172 (108 to 258 ) | 11.8 (7.4 to 17.5 ) | 616 (348 to 1034 ) | 14.0 (7.9 to 23.4 ) | 0.6 (0.4 to 0.7 ) |
| **Bermuda** | 0 (0 to 1 ) | 1.4 (1.1 to 1.8 ) | 0 (0 to 0 ) | 1.1 (0.8 to 1.4 ) | -1.1 (-1.8 to -0.4 ) | 0 (0 to 0 ) | 1.2 (0.9 to 1.5 ) | 0 (0 to 0 ) | 0.7 (0.5 to 0.9 ) | -2.0 (-2.3 to -1.6 ) | 19 (15 to 24 ) | 68.0 (53.4 to 86.0 ) | 8 (6 to 11 ) | 38.2 (27.1 to 51.0 ) | -2.0 (-2.3 to -1.6 ) |
| **Bhutan** | 1 (0 to 1 ) | 0.3 (0.1 to 0.4 ) | 1 (1 to 2 ) | 0.3 (0.2 to 0.5 ) | 0.4 (0.3 to 0.5 ) | 1 (0 to 1 ) | 0.3 (0.1 to 0.4 ) | 1 (1 to 2 ) | 0.3 (0.2 to 0.5 ) | 0.3 (0.2 to 0.5 ) | 31 (17 to 51 ) | 14.5 (7.8 to 23.5 ) | 58 (32 to 96 ) | 16.5 (9.2 to 27.5 ) | 0.3 (0.2 to 0.5 ) |
| **Bolivia (Plurinational State of)** | 18 (12 to 26 ) | 0.8 (0.6 to 1.2 ) | 31 (19 to 47 ) | 0.6 (0.4 to 1.0 ) | -0.8 (-0.9 to -0.8 ) | 17 (11 to 24 ) | 0.8 (0.5 to 1.1 ) | 28 (17 to 42 ) | 0.6 (0.4 to 0.9 ) | -0.9 (-1.0 to -0.8 ) | 986 (656 to 1418 ) | 44.3 (29.5 to 63.6 ) | 1624 (995 to 2483 ) | 33.8 (20.8 to 51.7 ) | -0.9 (-0.9 to -0.8 ) |
| **Bosnia and Herzegovina** | 30 (22 to 40 ) | 1.6 (1.2 to 2.1 ) | 11 (7 to 16 ) | 1.0 (0.6 to 1.4 ) | -1.6 (-2.3 to -0.9 ) | 27 (19 to 36 ) | 1.4 (1.0 to 1.8 ) | 9 (6 to 13 ) | 0.8 (0.5 to 1.2 ) | -1.8 (-2.5 to -1.1 ) | 1504 (1098 to 2014 ) | 78.0 (57.0 to 104.4 ) | 535 (343 to 751 ) | 46.9 (30.3 to 65.7 ) | -1.8 (-2.4 to -1.1 ) |
| **Botswana** | 2 (1 to 4 ) | 0.6 (0.3 to 0.9 ) | 6 (3 to 9 ) | 0.5 (0.3 to 0.8 ) | -0.6 (-1.3 to 0.1 ) | 2 (1 to 4 ) | 0.5 (0.3 to 0.9 ) | 5 (3 to 8 ) | 0.5 (0.3 to 0.7 ) | -0.6 (-1.3 to 0.1 ) | 121 (70 to 200 ) | 29.7 (17.2 to 49.2 ) | 288 (173 to 440 ) | 25.5 (15.3 to 39.0 ) | -0.6 (-1.3 to 0.1 ) |
| **Brazil** | 471 (440 to 503 ) | 0.8 (0.8 to 0.9 ) | 622 (577 to 672 ) | 0.7 (0.6 to 0.7 ) | -0.6 (-1.1 to -0.2 ) | 425 (397 to 455 ) | 0.7 (0.7 to 0.8 ) | 537 (499 to 579 ) | 0.6 (0.5 to 0.6 ) | -0.8 (-1.2 to -0.3 ) | 24610 (23013 to 26352 ) | 42.8 (40.0 to 45.8 ) | 30900 (28710 to 33343 ) | 33.9 (31.5 to 36.6 ) | -0.7 (-1.2 to -0.3 ) |
| **Brunei Darussalam** | 2 (1 to 3 ) | 1.5 (1.0 to 2.2 ) | 3 (2 to 4 ) | 1.2 (0.8 to 1.6 ) | -0.8 (-1.3 to -0.2 ) | 2 (1 to 2 ) | 1.3 (0.9 to 2.0 ) | 2 (1 to 3 ) | 0.9 (0.6 to 1.3 ) | -1.0 (-1.6 to -0.5 ) | 90 (61 to 135 ) | 74.8 (50.0 to 111.9 ) | 119 (83 to 167 ) | 52.3 (36.2 to 73.1 ) | -1.1 (-1.7 to -0.5 ) |
| **Bulgaria** | 69 (56 to 82 ) | 2.1 (1.7 to 2.5 ) | 35 (27 to 44 ) | 1.4 (1.1 to 1.9 ) | -1.1 (-2.9 to 0.6 ) | 60 (50 to 71 ) | 1.8 (1.5 to 2.2 ) | 29 (22 to 37 ) | 1.2 (0.9 to 1.6 ) | -1.3 (-3.1 to 0.5 ) | 3322 (2751 to 3958 ) | 102.6 (84.9 to 122.5 ) | 1614 (1243 to 2048 ) | 69.0 (53.0 to 88.0 ) | -1.3 (-3.1 to 0.5 ) |
| **Burkina Faso** | 5 (3 to 9 ) | 0.2 (0.1 to 0.3 ) | 18 (11 to 31 ) | 0.2 (0.1 to 0.4 ) | 0.7 (0.4 to 1.0 ) | 5 (3 to 8 ) | 0.2 (0.1 to 0.3 ) | 17 (10 to 28 ) | 0.2 (0.1 to 0.4 ) | 0.7 (0.4 to 1.0 ) | 272 (163 to 470 ) | 10.3 (6.2 to 17.9 ) | 965 (556 to 1624 ) | 12.9 (7.5 to 21.7 ) | 0.7 (0.5 to 1.0 ) |
| **Burundi** | 5 (3 to 8 ) | 0.3 (0.2 to 0.4 ) | 11 (6 to 18 ) | 0.2 (0.1 to 0.4 ) | -0.8 (-1.1 to -0.5 ) | 5 (3 to 7 ) | 0.3 (0.2 to 0.4 ) | 10 (6 to 16 ) | 0.2 (0.1 to 0.4 ) | -0.8 (-1.1 to -0.5 ) | 268 (174 to 411 ) | 15.0 (9.7 to 22.9 ) | 560 (324 to 950 ) | 11.9 (6.9 to 20.2 ) | -0.8 (-1.1 to -0.4 ) |
| **Cabo Verde** | 1 (1 to 2 ) | 1.2 (0.8 to 1.7 ) | 2 (1 to 3 ) | 0.8 (0.5 to 1.2 ) | -1.3 (-1.9 to -0.7 ) | 1 (1 to 2 ) | 1.1 (0.7 to 1.5 ) | 2 (1 to 3 ) | 0.7 (0.4 to 1.1 ) | -1.4 (-1.9 to -0.9 ) | 66 (44 to 96 ) | 62.4 (42.6 to 89.3 ) | 105 (67 to 167 ) | 40.6 (25.6 to 64.3 ) | -1.5 (-1.7 to -1.2 ) |
| **Cambodia** | 33 (22 to 49 ) | 1.0 (0.7 to 1.5 ) | 79 (48 to 125 ) | 1.1 (0.7 to 1.7 ) | 0.2 (0.1 to 0.4 ) | 31 (20 to 46 ) | 0.9 (0.6 to 1.4 ) | 71 (44 to 113 ) | 1.0 (0.6 to 1.6 ) | 0.2 (0.0 to 0.3 ) | 1819 (1169 to 2727 ) | 54.0 (35.0 to 80.8 ) | 4146 (2533 to 6604 ) | 57.1 (34.9 to 90.9 ) | 0.2 (0.1 to 0.3 ) |
| **Cameroon** | 10 (7 to 14 ) | 0.3 (0.2 to 0.5 ) | 44 (26 to 69 ) | 0.4 (0.2 to 0.6 ) | 0.7 (0.6 to 0.8 ) | 9 (6 to 13 ) | 0.3 (0.2 to 0.4 ) | 40 (23 to 63 ) | 0.4 (0.2 to 0.6 ) | 0.7 (0.5 to 0.8 ) | 526 (358 to 756 ) | 16.6 (11.4 to 23.7 ) | 2327 (1328 to 3648 ) | 20.5 (11.8 to 32.0 ) | 0.7 (0.5 to 0.8 ) |
| **Canada** | 182 (160 to 206 ) | 1.5 (1.3 to 1.7 ) | 95 (79 to 114 ) | 0.7 (0.6 to 0.8 ) | -2.3 (-2.7 to -1.8 ) | 135 (119 to 152 ) | 1.1 (1.0 to 1.2 ) | 59 (51 to 69 ) | 0.4 (0.4 to 0.5 ) | -2.8 (-3.3 to -2.4 ) | 7557 (6666 to 8518 ) | 61.5 (54.2 to 69.4 ) | 3356 (2866 to 3912 ) | 25.2 (21.4 to 29.4 ) | -2.8 (-3.3 to -2.3 ) |
| **Central African Republic** | 4 (2 to 6 ) | 0.4 (0.2 to 0.8 ) | 7 (4 to 14 ) | 0.4 (0.2 to 0.8 ) | -0.3 (-0.8 to 0.1 ) | 3 (2 to 6 ) | 0.4 (0.2 to 0.7 ) | 7 (3 to 13 ) | 0.4 (0.2 to 0.7 ) | -0.3 (-0.8 to 0.1 ) | 190 (108 to 344 ) | 22.0 (12.5 to 40.3 ) | 379 (191 to 748 ) | 20.3 (10.2 to 40.4 ) | -0.3 (-0.8 to 0.1 ) |
| **Chad** | 3 (2 to 5 ) | 0.2 (0.1 to 0.3 ) | 13 (8 to 21 ) | 0.3 (0.2 to 0.4 ) | 1.5 (1.3 to 1.7 ) | 3 (2 to 4 ) | 0.2 (0.1 to 0.3 ) | 12 (7 to 19 ) | 0.2 (0.2 to 0.4 ) | 1.5 (1.3 to 1.7 ) | 159 (97 to 259 ) | 9.0 (5.5 to 14.6 ) | 695 (428 to 1113 ) | 13.9 (8.7 to 22.2 ) | 1.5 (1.3 to 1.7 ) |
| **Chile** | 46 (38 to 55 ) | 0.9 (0.7 to 1.1 ) | 46 (36 to 57 ) | 0.6 (0.5 to 0.8 ) | -1.1 (-1.6 to -0.5 ) | 41 (34 to 49 ) | 0.8 (0.6 to 0.9 ) | 35 (27 to 44 ) | 0.5 (0.4 to 0.6 ) | -1.5 (-2.2 to -0.7 ) | 2334 (1929 to 2803 ) | 43.8 (36.3 to 52.6 ) | 2015 (1576 to 2508 ) | 27.0 (21.1 to 33.7 ) | -1.5 (-2.4 to -0.7 ) |
| **China** | 11847 (10038 to 13827 ) | 2.3 (2.0 to 2.7 ) | 12364 (9857 to 15097 ) | 2.2 (1.8 to 2.7 ) | -0.2 (-0.7 to 0.3 ) | 10612 (9006 to 12396 ) | 2.1 (1.8 to 2.4 ) | 9603 (7611 to 11776 ) | 1.7 (1.4 to 2.1 ) | -0.6 (-1.1 to -0.2 ) | 609492 (516859 to 712709 ) | 119.1 (101.0 to 139.2 ) | 542424 (430333 to 664815 ) | 98.4 (78.0 to 120.7 ) | -0.6 (-1.0 to -0.2 ) |
| **Colombia** | 126 (106 to 147 ) | 1.0 (0.8 to 1.1 ) | 110 (86 to 139 ) | 0.5 (0.4 to 0.7 ) | -2.0 (-2.8 to -1.2 ) | 115 (97 to 135 ) | 0.9 (0.8 to 1.0 ) | 93 (73 to 117 ) | 0.5 (0.4 to 0.6 ) | -2.2 (-3.0 to -1.4 ) | 6857 (5782 to 8043 ) | 52.4 (44.2 to 61.5 ) | 5455 (4266 to 6866 ) | 27.1 (21.2 to 34.1 ) | -2.2 (-3.0 to -1.3 ) |
| **Comoros** | 0 (0 to 1 ) | 0.3 (0.2 to 0.5 ) | 1 (1 to 2 ) | 0.3 (0.2 to 0.5 ) | 0.3 (-1.6 to 2.3 ) | 0 (0 to 1 ) | 0.3 (0.1 to 0.4 ) | 1 (1 to 1 ) | 0.3 (0.2 to 0.5 ) | 0.3 (-1.7 to 2.3 ) | 21 (10 to 34 ) | 15.1 (7.9 to 24.1 ) | 50 (30 to 79 ) | 17.4 (10.3 to 27.5 ) | 0.3 (-1.8 to 2.4 ) |
| **Congo** | 5 (3 to 7 ) | 0.6 (0.4 to 0.9 ) | 11 (7 to 18 ) | 0.5 (0.3 to 0.9 ) | -0.2 (-0.6 to 0.3 ) | 4 (2 to 6 ) | 0.6 (0.3 to 0.9 ) | 10 (6 to 16 ) | 0.5 (0.3 to 0.8 ) | -0.2 (-0.7 to 0.2 ) | 240 (141 to 364 ) | 31.9 (18.8 to 48.3 ) | 585 (340 to 937 ) | 28.0 (16.3 to 45.0 ) | -0.2 (-0.6 to 0.3 ) |
| **Cook Islands** | 0 (0 to 0 ) | 1.4 (0.9 to 2.2 ) | 0 (0 to 0 ) | 1.4 (0.8 to 2.2 ) | 0.0 (-0.2 to 0.1 ) | 0 (0 to 0 ) | 1.3 (0.8 to 2.0 ) | 0 (0 to 0 ) | 1.2 (0.7 to 1.9 ) | -0.3 (-0.4 to -0.2 ) | 5 (3 to 9 ) | 75.5 (47.0 to 118.8 ) | 4 (2 to 6 ) | 68.8 (40.0 to 112.0 ) | -0.3 (-0.4 to -0.2 ) |
| **Costa Rica** | 5 (4 to 6 ) | 0.4 (0.4 to 0.5 ) | 9 (7 to 11 ) | 0.5 (0.4 to 0.6 ) | 0.0 (-0.4 to 0.3 ) | 4 (4 to 5 ) | 0.4 (0.3 to 0.5 ) | 8 (6 to 9 ) | 0.4 (0.3 to 0.5 ) | -0.2 (-0.6 to 0.1 ) | 260 (216 to 311 ) | 22.1 (18.4 to 26.4 ) | 435 (352 to 540 ) | 21.7 (17.6 to 27.0 ) | -0.2 (-0.4 to 0.0 ) |
| **Coted'Ivoire** | 5 (3 to 8 ) | 0.1 (0.1 to 0.2 ) | 16 (9 to 25 ) | 0.2 (0.1 to 0.2 ) | 0.4 (0.1 to 0.7 ) | 5 (3 to 7 ) | 0.1 (0.1 to 0.2 ) | 14 (8 to 23 ) | 0.1 (0.1 to 0.2 ) | 0.4 (0.1 to 0.7 ) | 272 (179 to 404 ) | 6.9 (4.6 to 10.2 ) | 808 (451 to 1327 ) | 7.8 (4.4 to 12.8 ) | 0.4 (0.1 to 0.7 ) |
| **Croatia** | 37 (30 to 44 ) | 1.8 (1.5 to 2.2 ) | 15 (12 to 20 ) | 1.0 (0.8 to 1.3 ) | -1.7 (-3.5 to 0.2 ) | 29 (24 to 36 ) | 1.4 (1.2 to 1.7 ) | 10 (8 to 13 ) | 0.7 (0.5 to 0.9 ) | -2.3 (-4.0 to -0.5 ) | 1637 (1352 to 1984 ) | 80.6 (66.4 to 97.7 ) | 567 (442 to 715 ) | 38.9 (30.3 to 49.2 ) | -2.3 (-4.0 to -0.5 ) |
| **Cuba** | 75 (63 to 89 ) | 1.8 (1.5 to 2.1 ) | 39 (31 to 48 ) | 1.0 (0.8 to 1.2 ) | -1.9 (-2.5 to -1.3 ) | 62 (52 to 74 ) | 1.5 (1.2 to 1.7 ) | 29 (23 to 35 ) | 0.7 (0.6 to 0.9 ) | -2.2 (-2.8 to -1.5 ) | 3607 (3025 to 4276 ) | 84.0 (70.5 to 99.6 ) | 1632 (1296 to 2016 ) | 42.4 (33.7 to 52.4 ) | -2.2 (-2.8 to -1.6 ) |
| **Cyprus** | 1 (1 to 2 ) | 0.4 (0.3 to 0.6 ) | 3 (2 to 4 ) | 0.5 (0.3 to 0.7 ) | 0.4 (-0.5 to 1.3 ) | 1 (1 to 2 ) | 0.4 (0.2 to 0.5 ) | 2 (1 to 3 ) | 0.3 (0.2 to 0.4 ) | -0.4 (-1.2 to 0.5 ) | 63 (41 to 92 ) | 20.4 (13.3 to 29.6 ) | 111 (77 to 158 ) | 17.4 (11.9 to 24.7 ) | -0.4 (-1.2 to 0.5 ) |
| **Czechia** | 64 (52 to 78 ) | 1.5 (1.2 to 1.9 ) | 33 (26 to 41 ) | 0.9 (0.7 to 1.1 ) | -1.6 (-2.4 to -0.8 ) | 54 (44 to 66 ) | 1.3 (1.1 to 1.6 ) | 23 (19 to 29 ) | 0.7 (0.5 to 0.8 ) | -2.1 (-3.1 to -1.2 ) | 2980 (2445 to 3625 ) | 71.4 (58.5 to 86.9 ) | 1321 (1066 to 1654 ) | 37.5 (30.1 to 47.1 ) | -2.0 (-3.0 to -1.1 ) |
| **Democratic People's Republic of Korea** | 131 (81 to 210 ) | 1.7 (1.1 to 2.8 ) | 184 (98 to 325 ) | 1.7 (0.9 to 3.0 ) | -0.1 (-0.1 to 0.0 ) | 120 (74 to 188 ) | 1.6 (1.0 to 2.5 ) | 161 (87 to 290 ) | 1.5 (0.8 to 2.7 ) | -0.2 (-0.2 to -0.1 ) | 6915 (4250 to 10913 ) | 89.9 (55.5 to 141.4 ) | 9174 (4949 to 16535 ) | 85.0 (45.9 to 153.1 ) | -0.2 (-0.3 to -0.1 ) |
| **Democratic Republic of the Congo** | 36 (21 to 59 ) | 0.3 (0.2 to 0.5 ) | 85 (45 to 156 ) | 0.3 (0.2 to 0.5 ) | -0.3 (-0.5 to -0.1 ) | 33 (19 to 54 ) | 0.3 (0.2 to 0.5 ) | 78 (41 to 144 ) | 0.3 (0.1 to 0.5 ) | -0.4 (-0.6 to -0.1 ) | 1906 (1109 to 3140 ) | 16.0 (9.3 to 26.3 ) | 4476 (2366 to 8289 ) | 14.6 (7.7 to 27.1 ) | -0.4 (-0.6 to -0.1 ) |
| **Denmark** | 29 (25 to 34 ) | 1.4 (1.2 to 1.7 ) | 16 (13 to 20 ) | 0.9 (0.7 to 1.1 ) | -1.4 (-2.7 to -0.1 ) | 23 (20 to 27 ) | 1.1 (1.0 to 1.3 ) | 11 (9 to 13 ) | 0.6 (0.5 to 0.7 ) | -2.0 (-3.3 to -0.7 ) | 1276 (1087 to 1495 ) | 63.8 (54.3 to 74.8 ) | 619 (503 to 753 ) | 33.2 (27.0 to 40.3 ) | -1.9 (-3.2 to -0.7 ) |
| **Djibouti** | 0 (0 to 1 ) | 0.3 (0.2 to 0.4 ) | 2 (1 to 3 ) | 0.4 (0.2 to 0.6 ) | 0.8 (0.5 to 1.0 ) | 0 (0 to 1 ) | 0.2 (0.1 to 0.4 ) | 2 (1 to 3 ) | 0.3 (0.2 to 0.5 ) | 0.7 (0.5 to 1.0 ) | 19 (12 to 31 ) | 13.9 (8.5 to 22.0 ) | 98 (55 to 167 ) | 18.0 (10.2 to 30.6 ) | 0.7 (0.5 to 1.0 ) |
| **Dominica** | 0 (0 to 0 ) | 0.5 (0.4 to 0.7 ) | 0 (0 to 0 ) | 0.7 (0.5 to 1.1 ) | 1.1 (0.9 to 1.2 ) | 0 (0 to 0 ) | 0.5 (0.3 to 0.6 ) | 0 (0 to 0 ) | 0.6 (0.4 to 0.9 ) | 1.0 (0.8 to 1.1 ) | 7 (5 to 9 ) | 26.9 (19.4 to 36.2 ) | 10 (6 to 14 ) | 36.5 (24.2 to 53.1 ) | 0.9 (0.8 to 1.1 ) |
| **Dominican Republic** | 19 (14 to 26 ) | 0.8 (0.6 to 1.0 ) | 42 (27 to 60 ) | 0.9 (0.6 to 1.3 ) | 0.7 (0.1 to 1.2 ) | 17 (13 to 23 ) | 0.7 (0.5 to 0.9 ) | 36 (23 to 52 ) | 0.8 (0.5 to 1.2 ) | 0.5 (0.0 to 1.1 ) | 1012 (749 to 1363 ) | 38.7 (28.7 to 51.9 ) | 2067 (1309 to 2984 ) | 46.1 (29.2 to 66.6 ) | 0.6 (0.0 to 1.1 ) |
| **Ecuador** | 28 (23 to 33 ) | 0.8 (0.6 to 0.9 ) | 43 (32 to 58 ) | 0.6 (0.4 to 0.8 ) | -0.8 (-2.5 to 0.9 ) | 26 (21 to 30 ) | 0.7 (0.6 to 0.8 ) | 38 (28 to 51 ) | 0.5 (0.4 to 0.7 ) | -0.9 (-2.6 to 0.8 ) | 1517 (1259 to 1814 ) | 40.8 (33.9 to 48.7 ) | 2240 (1634 to 3004 ) | 31.2 (22.8 to 41.8 ) | -0.9 (-2.6 to 0.8 ) |
| **Egypt** | 145 (107 to 196 ) | 0.8 (0.6 to 1.0 ) | 417 (294 to 578 ) | 1.0 (0.7 to 1.4 ) | 0.9 (0.4 to 1.5 ) | 134 (99 to 181 ) | 0.7 (0.5 to 0.9 ) | 384 (270 to 536 ) | 0.9 (0.7 to 1.3 ) | 0.9 (0.4 to 1.5 ) | 7833 (5783 to 10555 ) | 39.8 (29.4 to 53.5 ) | 22511 (15832 to 31410 ) | 54.2 (38.1 to 75.6 ) | 1.0 (0.5 to 1.5 ) |
| **El Salvador** | 12 (9 to 14 ) | 0.7 (0.5 to 0.8 ) | 16 (12 to 22 ) | 0.7 (0.5 to 0.9 ) | -0.2 (-1.2 to 0.9 ) | 11 (9 to 13 ) | 0.6 (0.5 to 0.7 ) | 14 (11 to 19 ) | 0.6 (0.4 to 0.8 ) | -0.4 (-1.4 to 0.7 ) | 649 (521 to 791 ) | 35.7 (28.6 to 43.5 ) | 831 (618 to 1105 ) | 34.2 (25.4 to 45.5 ) | -0.4 (-1.5 to 0.7 ) |
| **Equatorial Guinea** | 1 (0 to 1 ) | 0.4 (0.2 to 0.7 ) | 3 (2 to 6 ) | 0.6 (0.3 to 1.0 ) | 1.1 (0.5 to 1.7 ) | 0 (0 to 1 ) | 0.4 (0.2 to 0.6 ) | 3 (2 to 5 ) | 0.5 (0.3 to 0.9 ) | 1.0 (0.4 to 1.7 ) | 28 (16 to 47 ) | 21.6 (12.4 to 36.5 ) | 171 (95 to 286 ) | 29.2 (16.2 to 48.6 ) | 1.0 (0.4 to 1.7 ) |
| **Eritrea** | 3 (2 to 5 ) | 0.3 (0.2 to 0.5 ) | 10 (6 to 16 ) | 0.4 (0.3 to 0.7 ) | 1.0 (0.7 to 1.2 ) | 3 (2 to 4 ) | 0.3 (0.2 to 0.4 ) | 9 (6 to 14 ) | 0.4 (0.2 to 0.6 ) | 1.0 (0.7 to 1.2 ) | 164 (106 to 247 ) | 15.7 (10.2 to 23.5 ) | 536 (316 to 822 ) | 21.4 (12.6 to 32.9 ) | 1.0 (0.7 to 1.2 ) |
| **Estonia** | 10 (8 to 12 ) | 1.6 (1.3 to 1.9 ) | 3 (2 to 4 ) | 0.6 (0.5 to 0.8 ) | -3.1 (-4.2 to -2.0 ) | 9 (7 to 10 ) | 1.4 (1.1 to 1.7 ) | 2 (2 to 3 ) | 0.5 (0.3 to 0.6 ) | -3.4 (-4.5 to -2.3 ) | 485 (394 to 585 ) | 79.1 (64.2 to 95.5 ) | 125 (94 to 157 ) | 26.4 (19.9 to 33.2 ) | -3.4 (-4.5 to -2.4 ) |
| **Eswatini** | 1 (1 to 2 ) | 0.5 (0.3 to 0.9 ) | 4 (2 to 7 ) | 0.9 (0.4 to 1.4 ) | 1.8 (1.5 to 2.1 ) | 1 (1 to 2 ) | 0.5 (0.2 to 0.8 ) | 4 (2 to 6 ) | 0.8 (0.4 to 1.3 ) | 1.7 (1.5 to 2.0 ) | 59 (32 to 103 ) | 25.5 (13.9 to 44.8 ) | 200 (97 to 328 ) | 43.3 (21.0 to 70.8 ) | 1.7 (1.4 to 2.0 ) |
| **Ethiopia** | 61 (43 to 84 ) | 0.4 (0.3 to 0.6 ) | 109 (83 to 141 ) | 0.3 (0.2 to 0.4 ) | -1.1 (-1.2 to -1.0 ) | 56 (39 to 77 ) | 0.4 (0.3 to 0.5 ) | 99 (76 to 129 ) | 0.3 (0.2 to 0.3 ) | -1.1 (-1.2 to -1.0 ) | 3210 (2244 to 4415 ) | 21.0 (14.6 to 28.9 ) | 5745 (4368 to 7444 ) | 14.9 (11.4 to 19.3 ) | -1.1 (-1.2 to -1.0 ) |
| **Fiji** | 2 (1 to 2 ) | 0.5 (0.3 to 0.8 ) | 2 (1 to 3 ) | 0.5 (0.3 to 0.7 ) | -0.4 (-0.9 to 0.2 ) | 1 (1 to 2 ) | 0.5 (0.3 to 0.7 ) | 1 (1 to 2 ) | 0.4 (0.2 to 0.6 ) | -0.4 (-0.9 to 0.1 ) | 84 (53 to 129 ) | 27.7 (17.2 to 42.2 ) | 88 (53 to 138 ) | 24.4 (14.7 to 38.2 ) | -0.4 (-0.9 to 0.1 ) |
| **Finland** | 22 (18 to 26 ) | 1.0 (0.9 to 1.3 ) | 15 (12 to 20 ) | 0.8 (0.6 to 1.0 ) | -0.8 (-1.6 to 0.0 ) | 17 (14 to 20 ) | 0.8 (0.7 to 1.0 ) | 9 (7 to 12 ) | 0.5 (0.4 to 0.6 ) | -1.5 (-2.3 to -0.7 ) | 925 (768 to 1123 ) | 44.5 (36.8 to 54.1 ) | 522 (415 to 661 ) | 28.0 (22.1 to 35.6 ) | -1.5 (-2.2 to -0.7 ) |
| **France** | 425 (356 to 510 ) | 1.8 (1.5 to 2.1 ) | 354 (286 to 436 ) | 1.6 (1.3 to 2.0 ) | -0.4 (-0.7 to 0.0 ) | 326 (273 to 391 ) | 1.4 (1.2 to 1.6 ) | 179 (148 to 214 ) | 0.8 (0.7 to 1.0 ) | -1.8 (-2.5 to -1.1 ) | 18027 (15135 to 21628 ) | 76.3 (64.1 to 91.5 ) | 9993 (8231 to 11978 ) | 46.2 (38.0 to 55.4 ) | -1.7 (-2.4 to -1.0 ) |
| **Gabon** | 2 (1 to 3 ) | 0.6 (0.3 to 1.0 ) | 4 (2 to 6 ) | 0.6 (0.3 to 0.9 ) | -0.1 (-0.4 to 0.2 ) | 2 (1 to 3 ) | 0.5 (0.3 to 0.9 ) | 3 (2 to 5 ) | 0.5 (0.3 to 0.8 ) | -0.1 (-0.4 to 0.2 ) | 100 (57 to 164 ) | 30.6 (17.4 to 50.0 ) | 193 (111 to 316 ) | 28.8 (16.6 to 47.0 ) | -0.1 (-0.4 to 0.2 ) |
| **Gambia** | 0 (0 to 0 ) | 0.1 (0.1 to 0.2 ) | 1 (1 to 2 ) | 0.1 (0.1 to 0.2 ) | 0.6 (-0.1 to 1.4 ) | 0 (0 to 0 ) | 0.1 (0.1 to 0.1 ) | 1 (1 to 1 ) | 0.1 (0.1 to 0.2 ) | 0.6 (-0.1 to 1.4 ) | 16 (10 to 24 ) | 5.2 (3.3 to 8.0 ) | 52 (30 to 83 ) | 6.1 (3.6 to 9.9 ) | 0.6 (-0.1 to 1.4 ) |
| **Georgia** | 52 (44 to 59 ) | 2.4 (2.1 to 2.8 ) | 14 (12 to 17 ) | 1.1 (0.9 to 1.3 ) | -2.7 (-4.8 to -0.5 ) | 46 (39 to 53 ) | 2.1 (1.8 to 2.5 ) | 12 (11 to 15 ) | 1.0 (0.8 to 1.1 ) | -2.7 (-4.8 to -0.6 ) | 2628 (2262 to 3037 ) | 121.8 (104.9 to 140.8 ) | 708 (602 to 832 ) | 55.2 (47.0 to 64.7 ) | -2.7 (-4.8 to -0.6 ) |
| **Germany** | 458 (374 to 543 ) | 1.5 (1.2 to 1.7 ) | 202 (159 to 249 ) | 0.7 (0.5 to 0.8 ) | -2.5 (-3.0 to -2.0 ) | 358 (294 to 425 ) | 1.1 (0.9 to 1.3 ) | 125 (101 to 154 ) | 0.4 (0.3 to 0.5 ) | -3.3 (-4.0 to -2.5 ) | 19909 (16387 to 23686 ) | 63.2 (52.0 to 75.1 ) | 7035 (5641 to 8650 ) | 24.0 (19.3 to 29.5 ) | -3.2 (-4.0 to -2.5 ) |
| **Ghana** | 13 (8 to 19 ) | 0.3 (0.2 to 0.4 ) | 26 (15 to 42 ) | 0.2 (0.1 to 0.3 ) | -0.9 (-1.0 to -0.8 ) | 11 (7 to 17 ) | 0.2 (0.1 to 0.4 ) | 23 (14 to 37 ) | 0.2 (0.1 to 0.3 ) | -0.9 (-1.0 to -0.8 ) | 660 (420 to 991 ) | 13.4 (8.5 to 20.1 ) | 1329 (803 to 2160 ) | 10.1 (6.1 to 16.4 ) | -0.9 (-1.0 to -0.8 ) |
| **Greece** | 73 (64 to 83 ) | 1.9 (1.7 to 2.2 ) | 34 (30 to 39 ) | 1.0 (0.9 to 1.2 ) | -2.1 (-2.6 to -1.5 ) | 55 (49 to 63 ) | 1.4 (1.3 to 1.6 ) | 23 (20 to 26 ) | 0.7 (0.6 to 0.8 ) | -2.4 (-3.0 to -1.7 ) | 3118 (2759 to 3530 ) | 81.3 (72.0 to 92.0 ) | 1288 (1137 to 1446 ) | 39.1 (34.6 to 43.9 ) | -2.4 (-3.0 to -1.7 ) |
| **Greenland** | 1 (1 to 2 ) | 4.0 (2.7 to 5.8 ) | 0 (0 to 1 ) | 1.5 (0.9 to 2.3 ) | -3.1 (-3.4 to -2.9 ) | 1 (1 to 1 ) | 3.6 (2.4 to 5.2 ) | 0 (0 to 0 ) | 1.2 (0.7 to 1.9 ) | -3.3 (-3.6 to -3.1 ) | 53 (35 to 77 ) | 201.0 (132.7 to 291.7 ) | 15 (9 to 23 ) | 68.6 (39.4 to 105.1 ) | -3.4 (-3.7 to -3.1 ) |
| **Grenada** | 0 (0 to 0 ) | 0.8 (0.7 to 1.0 ) | 0 (0 to 0 ) | 0.6 (0.4 to 0.7 ) | -1.3 (-2.2 to -0.4 ) | 0 (0 to 0 ) | 0.7 (0.6 to 0.9 ) | 0 (0 to 0 ) | 0.5 (0.4 to 0.6 ) | -1.5 (-2.3 to -0.6 ) | 12 (10 to 15 ) | 41.5 (32.8 to 51.7 ) | 10 (8 to 13 ) | 26.5 (20.2 to 34.1 ) | -1.5 (-2.3 to -0.7 ) |
| **Guam** | 1 (1 to 1 ) | 1.6 (1.3 to 2.0 ) | 1 (1 to 1 ) | 2.0 (1.6 to 2.5 ) | 0.9 (-0.6 to 2.5 ) | 1 (1 to 1 ) | 1.5 (1.2 to 1.9 ) | 1 (1 to 1 ) | 1.8 (1.4 to 2.2 ) | 0.8 (-0.9 to 2.5 ) | 56 (44 to 70 ) | 88.9 (69.6 to 111.7 ) | 56 (44 to 71 ) | 102.7 (80.8 to 130.8 ) | 0.7 (-0.9 to 2.2 ) |
| **Guatemala** | 21 (19 to 24 ) | 0.8 (0.7 to 0.9 ) | 29 (24 to 35 ) | 0.5 (0.4 to 0.6 ) | -1.9 (-2.5 to -1.4 ) | 20 (17 to 22 ) | 0.8 (0.7 to 0.9 ) | 26 (22 to 31 ) | 0.4 (0.4 to 0.5 ) | -2.0 (-2.5 to -1.5 ) | 1164 (1034 to 1291 ) | 45.0 (39.8 to 50.0 ) | 1549 (1294 to 1830 ) | 25.0 (20.9 to 29.6 ) | -2.0 (-2.5 to -1.5 ) |
| **Guinea** | 5 (3 to 7 ) | 0.3 (0.2 to 0.4 ) | 15 (9 to 24 ) | 0.3 (0.2 to 0.5 ) | 1.0 (0.8 to 1.1 ) | 4 (3 to 6 ) | 0.2 (0.2 to 0.3 ) | 13 (8 to 22 ) | 0.3 (0.2 to 0.5 ) | 1.0 (0.9 to 1.1 ) | 239 (157 to 344 ) | 13.0 (8.6 to 18.8 ) | 768 (457 to 1255 ) | 17.5 (10.5 to 28.6 ) | 1.0 (0.9 to 1.1 ) |
| **Guinea-Bissau** | 1 (1 to 2 ) | 0.4 (0.2 to 0.6 ) | 3 (2 to 5 ) | 0.5 (0.3 to 0.7 ) | 0.7 (0.6 to 0.8 ) | 1 (1 to 2 ) | 0.3 (0.2 to 0.5 ) | 3 (2 to 5 ) | 0.4 (0.3 to 0.7 ) | 0.7 (0.6 to 0.8 ) | 60 (39 to 92 ) | 19.4 (12.6 to 29.5 ) | 177 (108 to 276 ) | 23.8 (14.5 to 37.2 ) | 0.7 (0.6 to 0.8 ) |
| **Guyana** | 1 (1 to 1 ) | 0.3 (0.3 to 0.4 ) | 1 (1 to 2 ) | 0.5 (0.3 to 0.6 ) | 1.0 (0.0 to 2.0 ) | 1 (1 to 1 ) | 0.3 (0.2 to 0.4 ) | 1 (1 to 2 ) | 0.4 (0.3 to 0.6 ) | 0.9 (-0.5 to 2.3 ) | 52 (39 to 66 ) | 17.6 (13.4 to 22.3 ) | 65 (44 to 91 ) | 23.3 (15.9 to 32.5 ) | 0.9 (-0.4 to 2.3 ) |
| **Haiti** | 14 (8 to 20 ) | 0.6 (0.4 to 0.9 ) | 27 (16 to 42 ) | 0.5 (0.3 to 0.8 ) | -0.6 (-0.9 to -0.3 ) | 13 (7 to 18 ) | 0.6 (0.3 to 0.8 ) | 24 (15 to 38 ) | 0.5 (0.3 to 0.7 ) | -0.6 (-0.9 to -0.3 ) | 726 (425 to 1055 ) | 33.0 (19.3 to 47.9 ) | 1392 (832 to 2196 ) | 26.0 (15.5 to 41.0 ) | -0.6 (-0.9 to -0.3 ) |
| **Honduras** | 15 (10 to 21 ) | 1.0 (0.7 to 1.4 ) | 30 (15 to 54 ) | 0.8 (0.4 to 1.4 ) | -0.9 (-1.3 to -0.5 ) | 14 (10 to 19 ) | 0.9 (0.7 to 1.3 ) | 27 (13 to 49 ) | 0.7 (0.3 to 1.2 ) | -1.0 (-1.4 to -0.6 ) | 798 (568 to 1113 ) | 53.4 (38.2 to 74.0 ) | 1575 (767 to 2861 ) | 39.7 (19.4 to 71.8 ) | -1.0 (-1.4 to -0.6 ) |
| **Hungary** | 139 (114 to 167 ) | 3.1 (2.6 to 3.8 ) | 36 (27 to 46 ) | 1.1 (0.9 to 1.5 ) | -3.2 (-4.4 to -2.0 ) | 119 (98 to 143 ) | 2.7 (2.2 to 3.3 ) | 29 (22 to 36 ) | 0.9 (0.7 to 1.2 ) | -3.5 (-4.5 to -2.4 ) | 6552 (5408 to 7878 ) | 150.6 (124.0 to 181.5 ) | 1621 (1247 to 2057 ) | 52.1 (40.0 to 66.2 ) | -3.4 (-4.4 to -2.4 ) |
| **Iceland** | 1 (1 to 1 ) | 1.2 (1.0 to 1.4 ) | 2 (1 to 2 ) | 1.4 (1.1 to 1.7 ) | 0.8 (-0.5 to 2.1 ) | 1 (1 to 1 ) | 0.8 (0.7 to 1.0 ) | 1 (1 to 1 ) | 0.8 (0.6 to 1.0 ) | 0.0 (-1.2 to 1.2 ) | 50 (42 to 60 ) | 48.0 (39.7 to 57.0 ) | 58 (47 to 70 ) | 44.6 (36.6 to 54.4 ) | 0.0 (-1.1 to 1.1 ) |
| **India** | 1198 (1012 to 1417 ) | 0.4 (0.3 to 0.5 ) | 2585 (2193 to 3049 ) | 0.4 (0.4 to 0.5 ) | 0.3 (-0.1 to 0.8 ) | 1099 (928 to 1302 ) | 0.4 (0.3 to 0.4 ) | 2306 (1954 to 2728 ) | 0.4 (0.3 to 0.5 ) | 0.3 (-0.2 to 0.7 ) | 64132 (54081 to 76061 ) | 20.4 (17.2 to 24.1 ) | 133254 (112789 to 158191 ) | 22.3 (18.8 to 26.4 ) | 0.2 (-0.2 to 0.7 ) |
| **Indonesia** | 676 (499 to 845 ) | 1.0 (0.7 to 1.2 ) | 1286 (885 to 1796 ) | 1.1 (0.8 to 1.5 ) | 0.4 (0.3 to 0.5 ) | 624 (459 to 780 ) | 0.9 (0.7 to 1.1 ) | 1159 (795 to 1613 ) | 1.0 (0.7 to 1.4 ) | 0.3 (0.3 to 0.4 ) | 36684 (26874 to 45898 ) | 51.7 (38.0 to 64.5 ) | 67347 (46199 to 93781 ) | 57.7 (39.6 to 80.4 ) | 0.4 (0.3 to 0.4 ) |
| **Iran (Islamic Republic of)** | 148 (118 to 178 ) | 0.8 (0.6 to 0.9 ) | 330 (290 to 376 ) | 0.8 (0.7 to 0.9 ) | 0.0 (-0.3 to 0.3 ) | 139 (111 to 167 ) | 0.7 (0.6 to 0.9 ) | 299 (263 to 342 ) | 0.7 (0.6 to 0.9 ) | 0.0 (-0.3 to 0.3 ) | 8428 (6702 to 10150 ) | 43.1 (34.4 to 51.8 ) | 17302 (15179 to 19797 ) | 44.0 (38.5 to 50.5 ) | 0.0 (-0.3 to 0.3 ) |
| **Iraq** | 36 (21 to 54 ) | 0.6 (0.4 to 0.9 ) | 88 (55 to 139 ) | 0.5 (0.3 to 0.9 ) | -0.5 (-0.8 to -0.1 ) | 33 (20 to 50 ) | 0.6 (0.3 to 0.9 ) | 80 (50 to 128 ) | 0.5 (0.3 to 0.8 ) | -0.5 (-0.9 to -0.1 ) | 1916 (1159 to 2932 ) | 32.4 (19.5 to 49.6 ) | 4637 (2914 to 7398 ) | 28.6 (18.0 to 45.8 ) | -0.5 (-0.8 to -0.1 ) |
| **Ireland** | 14 (12 to 17 ) | 1.1 (0.9 to 1.3 ) | 18 (15 to 23 ) | 1.0 (0.8 to 1.3 ) | 0.1 (-0.6 to 0.7 ) | 11 (10 to 13 ) | 0.9 (0.7 to 1.0 ) | 11 (9 to 13 ) | 0.6 (0.5 to 0.7 ) | -0.9 (-1.9 to 0.1 ) | 644 (539 to 761 ) | 48.3 (40.4 to 57.0 ) | 612 (501 to 750 ) | 34.3 (27.9 to 42.2 ) | -0.9 (-1.9 to 0.0 ) |
| **Israel** | 14 (12 to 18 ) | 0.8 (0.6 to 0.9 ) | 19 (15 to 23 ) | 0.6 (0.4 to 0.7 ) | -1.1 (-1.8 to -0.4 ) | 12 (10 to 14 ) | 0.6 (0.5 to 0.8 ) | 13 (10 to 16 ) | 0.4 (0.3 to 0.5 ) | -1.6 (-2.3 to -1.0 ) | 663 (538 to 806 ) | 35.2 (28.5 to 42.8 ) | 721 (579 to 879 ) | 21.4 (17.2 to 26.1 ) | -1.6 (-2.3 to -1.0 ) |
| **Italy** | 283 (263 to 305 ) | 1.3 (1.2 to 1.4 ) | 132 (118 to 146 ) | 0.7 (0.7 to 0.8 ) | -1.9 (-2.1 to -1.7 ) | 227 (212 to 243 ) | 1.1 (1.0 to 1.1 ) | 89 (82 to 96 ) | 0.5 (0.5 to 0.5 ) | -2.5 (-2.6 to -2.3 ) | 12836 (12006 to 13746 ) | 60.6 (56.7 to 65.0 ) | 4997 (4603 to 5416 ) | 28.0 (25.7 to 30.4 ) | -2.5 (-2.6 to -2.3 ) |
| **Jamaica** | 5 (4 to 6 ) | 0.6 (0.5 to 0.7 ) | 8 (6 to 11 ) | 0.7 (0.5 to 1.0 ) | 0.1 (-1.5 to 1.7 ) | 4 (3 to 5 ) | 0.5 (0.4 to 0.6 ) | 7 (5 to 9 ) | 0.6 (0.4 to 0.8 ) | 0.0 (-1.5 to 1.6 ) | 245 (194 to 301 ) | 29.6 (23.5 to 36.3 ) | 376 (264 to 517 ) | 31.9 (22.4 to 43.8 ) | 0.0 (-1.5 to 1.6 ) |
| **Japan** | 558 (531 to 587 ) | 1.2 (1.1 to 1.2 ) | 259 (231 to 297 ) | 0.7 (0.6 to 0.8 ) | -1.8 (-2.1 to -1.4 ) | 387 (374 to 402 ) | 0.8 (0.8 to 0.8 ) | 132 (127 to 137 ) | 0.4 (0.3 to 0.4 ) | -2.8 (-3.0 to -2.6 ) | 21589 (20844 to 22389 ) | 45.8 (44.2 to 47.5 ) | 7465 (7180 to 7755 ) | 20.2 (19.4 to 21.0 ) | -2.7 (-2.9 to -2.6 ) |
| **Jordan** | 7 (4 to 9 ) | 0.6 (0.4 to 0.8 ) | 25 (16 to 37 ) | 0.5 (0.3 to 0.7 ) | -0.5 (-1.0 to -0.1 ) | 6 (4 to 9 ) | 0.5 (0.4 to 0.8 ) | 22 (15 to 34 ) | 0.4 (0.3 to 0.7 ) | -0.6 (-1.1 to 0.0 ) | 357 (243 to 509 ) | 30.9 (21.1 to 44.0 ) | 1293 (856 to 1941 ) | 25.5 (16.9 to 38.3 ) | -0.6 (-1.1 to 0.0 ) |
| **Kazakhstan** | 185 (169 to 202 ) | 2.8 (2.5 to 3.0 ) | 63 (56 to 69 ) | 0.8 (0.7 to 0.9 ) | -4.0 (-4.6 to -3.4 ) | 166 (151 to 182 ) | 2.5 (2.3 to 2.7 ) | 54 (48 to 60 ) | 0.7 (0.6 to 0.8 ) | -4.0 (-5.3 to -2.7 ) | 9609 (8760 to 10499 ) | 143.2 (130.4 to 156.7 ) | 3089 (2765 to 3431 ) | 40.2 (36.0 to 44.6 ) | -4.0 (-5.3 to -2.7 ) |
| **Kenya** | 5 (4 to 8 ) | 0.1 (0.1 to 0.1 ) | 24 (17 to 34 ) | 0.1 (0.1 to 0.2 ) | 1.4 (1.2 to 1.7 ) | 5 (3 to 8 ) | 0.1 (0.0 to 0.1 ) | 21 (15 to 31 ) | 0.1 (0.1 to 0.2 ) | 1.4 (1.2 to 1.6 ) | 287 (185 to 440 ) | 4.2 (2.7 to 6.4 ) | 1214 (863 to 1763 ) | 6.5 (4.6 to 9.3 ) | 1.4 (1.2 to 1.5 ) |
| **Kiribati** | 0 (0 to 0 ) | 0.9 (0.6 to 1.4 ) | 1 (0 to 1 ) | 1.2 (0.7 to 1.9 ) | 0.9 (0.9 to 1.0 ) | 0 (0 to 0 ) | 0.9 (0.6 to 1.3 ) | 1 (0 to 1 ) | 1.2 (0.7 to 1.8 ) | 0.9 (0.8 to 1.0 ) | 16 (10 to 23 ) | 53.4 (34.2 to 80.2 ) | 34 (20 to 53 ) | 69.2 (40.6 to 109.9 ) | 0.9 (0.8 to 0.9 ) |
| **Kuwait** | 3 (2 to 4 ) | 0.3 (0.3 to 0.4 ) | 9 (7 to 13 ) | 0.3 (0.3 to 0.5 ) | 0.3 (-0.8 to 1.4 ) | 3 (2 to 3 ) | 0.3 (0.2 to 0.4 ) | 8 (6 to 11 ) | 0.3 (0.2 to 0.4 ) | 0.3 (-0.8 to 1.4 ) | 153 (117 to 196 ) | 17.1 (13.1 to 21.8 ) | 471 (347 to 633 ) | 18.0 (13.4 to 24.1 ) | 0.3 (-0.8 to 1.5 ) |
| **Kyrgyzstan** | 33 (26 to 39 ) | 2.0 (1.6 to 2.4 ) | 21 (16 to 27 ) | 0.8 (0.6 to 1.0 ) | -3.0 (-3.7 to -2.3 ) | 30 (24 to 36 ) | 1.8 (1.5 to 2.2 ) | 19 (14 to 23 ) | 0.7 (0.5 to 0.8 ) | -3.1 (-3.8 to -2.4 ) | 1719 (1394 to 2087 ) | 103.3 (84.0 to 125.3 ) | 1070 (820 to 1335 ) | 39.0 (30.0 to 48.6 ) | -3.1 (-3.8 to -2.4 ) |
| **Lao People's Democratic Republic** | 15 (8 to 24 ) | 1.1 (0.6 to 1.8 ) | 34 (20 to 55 ) | 1.1 (0.6 to 1.8 ) | 0.0 (-0.2 to 0.2 ) | 14 (8 to 22 ) | 1.0 (0.6 to 1.7 ) | 31 (18 to 51 ) | 1.0 (0.6 to 1.6 ) | 0.0 (-0.1 to 0.2 ) | 796 (459 to 1316 ) | 58.5 (33.8 to 96.2 ) | 1826 (1071 to 2973 ) | 58.2 (34.2 to 94.7 ) | 0.0 (-0.1 to 0.2 ) |
| **Latvia** | 14 (12 to 17 ) | 1.4 (1.2 to 1.7 ) | 5 (4 to 6 ) | 0.8 (0.6 to 1.0 ) | -2.2 (-3.9 to -0.4 ) | 12 (10 to 15 ) | 1.2 (1.0 to 1.5 ) | 4 (3 to 5 ) | 0.6 (0.4 to 0.7 ) | -2.5 (-4.2 to -0.8 ) | 684 (563 to 832 ) | 68.2 (56.2 to 83.0 ) | 216 (167 to 272 ) | 32.7 (25.1 to 41.2 ) | -2.5 (-4.2 to -0.8 ) |
| **Lebanon** | 9 (6 to 13 ) | 0.9 (0.6 to 1.3 ) | 18 (12 to 26 ) | 0.7 (0.5 to 1.0 ) | -0.8 (-1.0 to -0.5 ) | 8 (5 to 12 ) | 0.8 (0.5 to 1.2 ) | 16 (11 to 23 ) | 0.6 (0.4 to 0.9 ) | -0.9 (-1.1 to -0.6 ) | 473 (304 to 691 ) | 45.6 (29.4 to 66.5 ) | 893 (598 to 1299 ) | 34.7 (23.2 to 50.5 ) | -0.8 (-1.1 to -0.6 ) |
| **Lesotho** | 1 (1 to 2 ) | 0.3 (0.2 to 0.5 ) | 6 (3 to 10 ) | 0.8 (0.5 to 1.3 ) | 3.5 (3.1 to 4.0 ) | 1 (1 to 2 ) | 0.3 (0.2 to 0.4 ) | 5 (3 to 9 ) | 0.7 (0.4 to 1.2 ) | 3.5 (3.1 to 3.9 ) | 68 (41 to 117 ) | 14.6 (8.7 to 25.1 ) | 303 (169 to 487 ) | 41.7 (23.2 to 67.0 ) | 3.5 (3.1 to 3.9 ) |
| **Liberia** | 2 (1 to 3 ) | 0.2 (0.1 to 0.3 ) | 5 (3 to 9 ) | 0.3 (0.1 to 0.5 ) | 0.5 (0.2 to 0.8 ) | 2 (1 to 3 ) | 0.2 (0.1 to 0.3 ) | 5 (2 to 8 ) | 0.2 (0.1 to 0.4 ) | 0.5 (0.2 to 0.7 ) | 97 (61 to 150 ) | 11.8 (7.5 to 18.1 ) | 277 (142 to 492 ) | 13.6 (7.0 to 24.3 ) | 0.5 (0.2 to 0.8 ) |
| **Libya** | 12 (7 to 19 ) | 0.9 (0.6 to 1.4 ) | 30 (18 to 45 ) | 0.9 (0.5 to 1.4 ) | -0.1 (-0.6 to 0.4 ) | 11 (7 to 17 ) | 0.8 (0.5 to 1.3 ) | 27 (16 to 41 ) | 0.8 (0.5 to 1.2 ) | -0.1 (-0.7 to 0.4 ) | 631 (379 to 969 ) | 47.2 (28.3 to 72.5 ) | 1496 (917 to 2315 ) | 45.2 (27.7 to 70.0 ) | -0.1 (-0.7 to 0.4 ) |
| **Lithuania** | 21 (17 to 25 ) | 1.5 (1.2 to 1.8 ) | 7 (6 to 9 ) | 0.8 (0.6 to 1.0 ) | -2.0 (-2.8 to -1.1 ) | 17 (14 to 21 ) | 1.2 (1.0 to 1.5 ) | 5 (4 to 7 ) | 0.6 (0.5 to 0.8 ) | -2.3 (-4.0 to -0.6 ) | 961 (787 to 1169 ) | 68.6 (56.3 to 83.5 ) | 306 (236 to 385 ) | 33.7 (26.0 to 42.5 ) | -2.3 (-4.0 to -0.6 ) |
| **Luxembourg** | 3 (2 to 3 ) | 1.6 (1.4 to 1.8 ) | 2 (1 to 2 ) | 0.7 (0.6 to 0.8 ) | -2.8 (-3.5 to -2.1 ) | 2 (2 to 2 ) | 1.3 (1.1 to 1.4 ) | 1 (1 to 1 ) | 0.4 (0.4 to 0.5 ) | -3.5 (-4.3 to -2.6 ) | 115 (102 to 131 ) | 70.5 (62.2 to 79.9 ) | 62 (53 to 70 ) | 23.6 (20.5 to 26.8 ) | -3.6 (-4.3 to -2.8 ) |
| **Madagascar** | 10 (7 to 14 ) | 0.3 (0.2 to 0.4 ) | 27 (17 to 40 ) | 0.3 (0.2 to 0.4 ) | 0.1 (0.0 to 0.2 ) | 9 (6 to 13 ) | 0.2 (0.2 to 0.3 ) | 25 (16 to 37 ) | 0.2 (0.2 to 0.4 ) | 0.1 (0.0 to 0.2 ) | 531 (361 to 747 ) | 14.0 (9.6 to 19.7 ) | 1431 (901 to 2132 ) | 14.3 (9.0 to 21.2 ) | 0.1 (0.0 to 0.2 ) |
| **Malawi** | 2 (1 to 3 ) | 0.1 (0.0 to 0.1 ) | 7 (4 to 10 ) | 0.1 (0.1 to 0.2 ) | 1.0 (0.8 to 1.2 ) | 2 (1 to 3 ) | 0.1 (0.0 to 0.1 ) | 6 (4 to 9 ) | 0.1 (0.1 to 0.1 ) | 0.9 (0.7 to 1.1 ) | 119 (77 to 180 ) | 4.0 (2.6 to 5.9 ) | 348 (212 to 545 ) | 5.3 (3.2 to 8.2 ) | 0.9 (0.7 to 1.1 ) |
| **Malaysia** | 38 (27 to 53 ) | 0.6 (0.4 to 0.8 ) | 98 (68 to 135 ) | 0.7 (0.5 to 0.9 ) | 0.6 (0.3 to 1.0 ) | 35 (24 to 48 ) | 0.5 (0.4 to 0.7 ) | 84 (58 to 117 ) | 0.6 (0.4 to 0.8 ) | 0.5 (0.1 to 0.8 ) | 2000 (1396 to 2755 ) | 29.1 (20.3 to 39.9 ) | 4749 (3301 to 6617 ) | 33.1 (23.0 to 46.1 ) | 0.4 (0.1 to 0.8 ) |
| **Maldives** | 0 (0 to 1 ) | 0.7 (0.3 to 1.0 ) | 1 (1 to 2 ) | 0.4 (0.2 to 0.5 ) | -1.9 (-2.4 to -1.4 ) | 0 (0 to 1 ) | 0.6 (0.3 to 0.9 ) | 1 (1 to 1 ) | 0.3 (0.2 to 0.5 ) | -2.1 (-2.6 to -1.6 ) | 22 (12 to 35 ) | 34.3 (18.1 to 54.1 ) | 59 (38 to 85 ) | 18.4 (12.0 to 26.5 ) | -2.1 (-2.6 to -1.6 ) |
| **Mali** | 4 (3 to 6 ) | 0.2 (0.1 to 0.2 ) | 13 (8 to 20 ) | 0.2 (0.1 to 0.3 ) | 0.4 (0.2 to 0.6 ) | 4 (3 to 5 ) | 0.2 (0.1 to 0.2 ) | 12 (7 to 18 ) | 0.2 (0.1 to 0.3 ) | 0.4 (0.2 to 0.6 ) | 220 (153 to 310 ) | 8.6 (6.0 to 12.0 ) | 688 (433 to 1056 ) | 9.8 (6.2 to 14.9 ) | 0.4 (0.2 to 0.6 ) |
| **Malta** | 1 (1 to 1 ) | 0.6 (0.5 to 0.8 ) | 1 (1 to 1 ) | 0.6 (0.5 to 0.8 ) | 0.1 (-0.9 to 1.2 ) | 1 (1 to 1 ) | 0.5 (0.4 to 0.6 ) | 1 (1 to 1 ) | 0.4 (0.3 to 0.5 ) | -0.5 (-1.5 to 0.5 ) | 45 (36 to 55 ) | 29.5 (23.7 to 36.6 ) | 37 (29 to 47 ) | 23.2 (18.2 to 29.3 ) | -0.5 (-1.5 to 0.5 ) |
| **Marshall Islands** | 0 (0 to 0 ) | 1.3 (0.8 to 2.2 ) | 0 (0 to 1 ) | 1.8 (1.0 to 2.9 ) | 1.0 (0.9 to 1.1 ) | 0 (0 to 0 ) | 1.2 (0.7 to 2.0 ) | 0 (0 to 1 ) | 1.6 (0.9 to 2.6 ) | 0.9 (0.8 to 1.0 ) | 12 (7 to 20 ) | 73.6 (42.1 to 122.6 ) | 22 (12 to 36 ) | 94.9 (53.1 to 153.2 ) | 0.8 (0.7 to 0.9 ) |
| **Mauritania** | 2 (1 to 3 ) | 0.3 (0.2 to 0.4 ) | 4 (3 to 6 ) | 0.3 (0.2 to 0.4 ) | 0.0 (-0.4 to 0.4 ) | 2 (1 to 3 ) | 0.3 (0.2 to 0.4 ) | 4 (2 to 6 ) | 0.3 (0.2 to 0.4 ) | -0.1 (-0.5 to 0.3 ) | 99 (62 to 151 ) | 15.0 (9.4 to 22.8 ) | 213 (132 to 340 ) | 14.6 (9.1 to 23.1 ) | -0.1 (-0.5 to 0.3 ) |
| **Mauritius** | 4 (3 to 4 ) | 0.7 (0.6 to 0.8 ) | 3 (3 to 3 ) | 0.7 (0.6 to 0.7 ) | -0.4 (-1.6 to 0.9 ) | 3 (3 to 4 ) | 0.7 (0.6 to 0.7 ) | 3 (2 to 3 ) | 0.6 (0.5 to 0.6 ) | -0.5 (-1.6 to 0.7 ) | 184 (161 to 209 ) | 37.6 (32.9 to 42.6 ) | 151 (131 to 171 ) | 32.1 (27.9 to 36.4 ) | -0.5 (-1.6 to 0.7 ) |
| **Mexico** | 240 (230 to 250 ) | 0.8 (0.8 to 0.8 ) | 252 (226 to 279 ) | 0.5 (0.4 to 0.5 ) | -1.6 (-1.9 to -1.4 ) | 220 (211 to 228 ) | 0.7 (0.7 to 0.7 ) | 219 (198 to 242 ) | 0.4 (0.4 to 0.5 ) | -1.7 (-2.0 to -1.5 ) | 13109 (12592 to 13635 ) | 41.7 (40.0 to 43.3 ) | 12854 (11588 to 14168 ) | 24.9 (22.4 to 27.4 ) | -1.7 (-2.0 to -1.5 ) |
| **Micronesia (Federated States of)** | 1 (0 to 1 ) | 1.6 (0.9 to 2.5 ) | 1 (0 to 1 ) | 1.8 (1.0 to 3.0 ) | 0.6 (0.5 to 0.6 ) | 1 (0 to 1 ) | 1.4 (0.8 to 2.3 ) | 1 (0 to 1 ) | 1.7 (1.0 to 2.7 ) | 0.5 (0.5 to 0.6 ) | 30 (17 to 50 ) | 83.5 (48.3 to 136.9 ) | 39 (22 to 63 ) | 98.5 (55.5 to 159.6 ) | 0.5 (0.5 to 0.6 ) |
| **Monaco** | 0 (0 to 0 ) | 2.5 (1.7 to 3.5 ) | 0 (0 to 1 ) | 4.2 (2.4 to 6.5 ) | 1.7 (1.6 to 1.8 ) | 0 (0 to 0 ) | 1.8 (1.3 to 2.6 ) | 0 (0 to 0 ) | 2.7 (1.6 to 4.1 ) | 1.2 (1.0 to 1.3 ) | 11 (8 to 15 ) | 104.1 (71.4 to 147.4 ) | 16 (9 to 24 ) | 149.8 (89.6 to 231.5 ) | 1.2 (1.0 to 1.3 ) |
| **Mongolia** | 11 (7 to 16 ) | 1.5 (0.9 to 2.2 ) | 17 (11 to 25 ) | 1.2 (0.8 to 1.8 ) | -0.6 (-1.6 to 0.5 ) | 10 (6 to 15 ) | 1.3 (0.8 to 2.0 ) | 15 (10 to 22 ) | 1.1 (0.7 to 1.6 ) | -0.7 (-1.7 to 0.4 ) | 586 (367 to 866 ) | 78.2 (49.1 to 115.1 ) | 859 (552 to 1272 ) | 62.3 (40.0 to 92.3 ) | -0.7 (-1.7 to 0.3 ) |
| **Montenegro** | 6 (4 to 7 ) | 2.2 (1.6 to 2.8 ) | 4 (3 to 5 ) | 1.6 (1.1 to 2.2 ) | -0.8 (-1.5 to -0.1 ) | 5 (3 to 6 ) | 1.8 (1.3 to 2.3 ) | 3 (2 to 4 ) | 1.3 (0.9 to 1.7 ) | -1.0 (-1.7 to -0.3 ) | 258 (194 to 332 ) | 101.4 (76.2 to 130.6 ) | 165 (117 to 221 ) | 72.4 (51.6 to 97.0 ) | -1.0 (-1.6 to -0.3 ) |
| **Morocco** | 29 (18 to 44 ) | 0.3 (0.2 to 0.5 ) | 32 (19 to 55 ) | 0.2 (0.1 to 0.4 ) | -1.4 (-1.6 to -1.3 ) | 26 (17 to 40 ) | 0.3 (0.2 to 0.5 ) | 29 (17 to 50 ) | 0.2 (0.1 to 0.3 ) | -1.4 (-1.6 to -1.3 ) | 1510 (940 to 2305 ) | 17.0 (10.6 to 26.0 ) | 1651 (970 to 2841 ) | 11.0 (6.5 to 18.9 ) | -1.4 (-1.5 to -1.3 ) |
| **Mozambique** | 4 (3 to 6 ) | 0.1 (0.1 to 0.1 ) | 15 (9 to 24 ) | 0.2 (0.1 to 0.3 ) | 1.7 (1.5 to 1.9 ) | 4 (2 to 5 ) | 0.1 (0.1 to 0.1 ) | 14 (8 to 22 ) | 0.1 (0.1 to 0.2 ) | 1.7 (1.5 to 1.9 ) | 205 (136 to 297 ) | 5.0 (3.3 to 7.2 ) | 796 (474 to 1268 ) | 8.3 (5.0 to 13.2 ) | 1.7 (1.5 to 1.9 ) |
| **Myanmar** | 176 (108 to 261 ) | 1.2 (0.7 to 1.7 ) | 215 (135 to 324 ) | 1.0 (0.6 to 1.5 ) | -0.5 (-0.6 to -0.5 ) | 163 (101 to 240 ) | 1.1 (0.7 to 1.6 ) | 195 (123 to 294 ) | 0.9 (0.6 to 1.3 ) | -0.6 (-0.6 to -0.5 ) | 9638 (5970 to 14223 ) | 61.9 (38.5 to 91.0 ) | 11484 (7229 to 17356 ) | 52.3 (33.0 to 78.9 ) | -0.5 (-0.6 to -0.5 ) |
| **Namibia** | 1 (1 to 1 ) | 0.2 (0.1 to 0.3 ) | 2 (1 to 4 ) | 0.2 (0.1 to 0.4 ) | 0.5 (0.2 to 0.8 ) | 1 (1 to 1 ) | 0.2 (0.1 to 0.3 ) | 2 (1 to 3 ) | 0.2 (0.1 to 0.3 ) | 0.4 (0.1 to 0.8 ) | 48 (32 to 70 ) | 10.7 (7.2 to 15.6 ) | 116 (68 to 186 ) | 12.1 (7.1 to 19.4 ) | 0.4 (0.1 to 0.8 ) |
| **Nauru** | 0 (0 to 0 ) | 2.2 (1.1 to 3.4 ) | 0 (0 to 0 ) | 2.5 (1.2 to 3.7 ) | 0.4 (0.4 to 0.5 ) | 0 (0 to 0 ) | 2.0 (1.0 to 3.2 ) | 0 (0 to 0 ) | 2.2 (1.1 to 3.4 ) | 0.4 (0.3 to 0.5 ) | 4 (2 to 7 ) | 115.4 (59.8 to 184.3 ) | 6 (3 to 9 ) | 130.8 (66.1 to 199.8 ) | 0.4 (0.3 to 0.5 ) |
| **Nepal** | 19 (11 to 31 ) | 0.3 (0.2 to 0.5 ) | 40 (24 to 66 ) | 0.3 (0.2 to 0.5 ) | 0.2 (0.1 to 0.4 ) | 18 (10 to 28 ) | 0.3 (0.2 to 0.4 ) | 36 (21 to 60 ) | 0.3 (0.2 to 0.5 ) | 0.2 (0.1 to 0.3 ) | 1029 (599 to 1650 ) | 15.9 (9.3 to 25.5 ) | 2085 (1232 to 3506 ) | 17.0 (10.1 to 28.4 ) | 0.2 (0.1 to 0.3 ) |
| **Netherlands** | 92 (79 to 107 ) | 1.5 (1.3 to 1.7 ) | 59 (49 to 72 ) | 1.1 (0.9 to 1.3 ) | -0.9 (-1.2 to -0.7 ) | 63 (54 to 73 ) | 1.0 (0.9 to 1.2 ) | 36 (30 to 42 ) | 0.6 (0.5 to 0.8 ) | -1.4 (-1.7 to -1.1 ) | 3536 (3030 to 4103 ) | 56.2 (48.1 to 65.2 ) | 2025 (1694 to 2400 ) | 36.4 (30.4 to 43.1 ) | -1.3 (-1.7 to -1.0 ) |
| **New Zealand** | 11 (9 to 12 ) | 0.8 (0.7 to 0.9 ) | 14 (12 to 16 ) | 0.7 (0.6 to 0.8 ) | -0.3 (-1.2 to 0.6 ) | 8 (7 to 10 ) | 0.6 (0.5 to 0.7 ) | 9 (8 to 11 ) | 0.5 (0.4 to 0.6 ) | -0.9 (-1.7 to 0.0 ) | 468 (403 to 539 ) | 33.8 (29.0 to 38.8 ) | 501 (424 to 588 ) | 26.3 (22.3 to 30.9 ) | -0.9 (-1.7 to 0.0 ) |
| **Nicaragua** | 5 (3 to 6 ) | 0.4 (0.3 to 0.5 ) | 8 (6 to 12 ) | 0.3 (0.2 to 0.4 ) | -1.1 (-1.5 to -0.7 ) | 4 (3 to 6 ) | 0.4 (0.3 to 0.5 ) | 7 (5 to 10 ) | 0.3 (0.2 to 0.4 ) | -1.2 (-1.7 to -0.8 ) | 256 (184 to 350 ) | 20.4 (14.8 to 27.9 ) | 417 (281 to 592 ) | 15.0 (10.1 to 21.3 ) | -1.3 (-1.7 to -0.8 ) |
| **Niger** | 4 (2 to 6 ) | 0.2 (0.1 to 0.3 ) | 11 (6 to 20 ) | 0.2 (0.1 to 0.3 ) | 0.2 (0.0 to 0.4 ) | 3 (2 to 6 ) | 0.1 (0.1 to 0.2 ) | 10 (6 to 19 ) | 0.2 (0.1 to 0.3 ) | 0.2 (0.0 to 0.4 ) | 190 (112 to 323 ) | 8.2 (4.8 to 13.8 ) | 595 (331 to 1107 ) | 8.7 (4.9 to 16.0 ) | 0.2 (0.0 to 0.4 ) |
| **Nigeria** | 12 (9 to 16 ) | 0.0 (0.0 to 0.1 ) | 38 (24 to 55 ) | 0.1 (0.0 to 0.1 ) | 0.6 (0.4 to 0.9 ) | 11 (8 to 15 ) | 0.0 (0.0 to 0.1 ) | 34 (22 to 49 ) | 0.0 (0.0 to 0.1 ) | 0.6 (0.3 to 0.8 ) | 630 (447 to 845 ) | 2.2 (1.6 to 3.0 ) | 1973 (1268 to 2844 ) | 2.7 (1.7 to 3.9 ) | 0.6 (0.3 to 0.9 ) |
| **Niue** | 0 (0 to 0 ) | 1.1 (0.7 to 1.8 ) | 0 (0 to 0 ) | 1.5 (1.0 to 2.3 ) | 0.9 (0.6 to 1.3 ) | 0 (0 to 0 ) | 1.0 (0.6 to 1.6 ) | 0 (0 to 0 ) | 1.4 (0.9 to 2.1 ) | 0.9 (0.5 to 1.2 ) | 0 (0 to 1 ) | 60.2 (36.6 to 94.2 ) | 0 (0 to 1 ) | 80.4 (52.3 to 121.5 ) | 0.9 (0.6 to 1.3 ) |
| **North Macedonia** | 11 (8 to 13 ) | 1.3 (1.0 to 1.7 ) | 8 (6 to 10 ) | 0.8 (0.6 to 1.1 ) | -1.6 (-2.3 to -0.9 ) | 9 (7 to 12 ) | 1.2 (0.9 to 1.5 ) | 6 (5 to 8 ) | 0.7 (0.5 to 0.9 ) | -1.8 (-2.4 to -1.1 ) | 519 (405 to 658 ) | 65.4 (51.1 to 82.9 ) | 354 (263 to 458 ) | 39.3 (29.2 to 50.9 ) | -1.7 (-2.4 to -1.1 ) |
| **Northern Mariana Islands** | 1 (0 to 1 ) | 2.1 (1.2 to 3.2 ) | 0 (0 to 0 ) | 1.6 (1.0 to 2.3 ) | -1.1 (-1.3 to -0.9 ) | 0 (0 to 1 ) | 1.9 (1.1 to 2.9 ) | 0 (0 to 0 ) | 1.4 (0.9 to 2.0 ) | -1.1 (-1.3 to -0.9 ) | 26 (15 to 41 ) | 107.6 (61.7 to 168.2 ) | 13 (9 to 20 ) | 79.8 (53.0 to 118.0 ) | -1.1 (-1.3 to -0.9 ) |
| **Norway** | 12 (11 to 13 ) | 0.7 (0.6 to 0.8 ) | 11 (10 to 13 ) | 0.6 (0.5 to 0.7 ) | -0.6 (-1.1 to 0.0 ) | 9 (8 to 10 ) | 0.5 (0.5 to 0.6 ) | 7 (6 to 7 ) | 0.3 (0.3 to 0.4 ) | -1.4 (-1.9 to -0.9 ) | 500 (460 to 542 ) | 30.0 (27.6 to 32.6 ) | 373 (335 to 414 ) | 19.1 (17.2 to 21.3 ) | -1.4 (-1.9 to -0.8 ) |
| **Oman** | 1 (1 to 2 ) | 0.2 (0.1 to 0.2 ) | 3 (2 to 5 ) | 0.1 (0.1 to 0.2 ) | -1.5 (-1.9 to -1.1 ) | 1 (1 to 2 ) | 0.1 (0.1 to 0.2 ) | 3 (2 to 4 ) | 0.1 (0.1 to 0.1 ) | -1.6 (-2.0 to -1.1 ) | 68 (40 to 106 ) | 8.0 (4.8 to 12.5 ) | 145 (95 to 235 ) | 5.0 (3.3 to 8.1 ) | -1.6 (-2.0 to -1.1 ) |
| **Pakistan** | 161 (119 to 211 ) | 0.5 (0.4 to 0.6 ) | 562 (398 to 766 ) | 0.6 (0.4 to 0.9 ) | 0.8 (0.7 to 0.9 ) | 147 (108 to 194 ) | 0.4 (0.3 to 0.6 ) | 508 (360 to 695 ) | 0.6 (0.4 to 0.8 ) | 0.8 (0.7 to 0.9 ) | 8471 (6205 to 11171 ) | 25.5 (18.6 to 33.5 ) | 29162 (20691 to 39868 ) | 32.3 (22.9 to 44.2 ) | 0.8 (0.6 to 0.9 ) |
| **Palau** | 0 (0 to 0 ) | 2.5 (1.6 to 3.8 ) | 0 (0 to 0 ) | 3.1 (2.1 to 4.4 ) | 0.6 (0.3 to 0.9 ) | 0 (0 to 0 ) | 2.3 (1.5 to 3.5 ) | 0 (0 to 0 ) | 2.8 (1.9 to 4.0 ) | 0.5 (0.3 to 0.8 ) | 9 (6 to 14 ) | 135.9 (85.3 to 205.5 ) | 10 (7 to 15 ) | 162.1 (108.7 to 232.5 ) | 0.5 (0.2 to 0.8 ) |
| **Palestine** | 6 (4 to 10 ) | 1.0 (0.6 to 1.6 ) | 16 (11 to 23 ) | 0.8 (0.6 to 1.2 ) | -0.6 (-0.8 to -0.4 ) | 6 (4 to 9 ) | 1.0 (0.6 to 1.5 ) | 15 (10 to 22 ) | 0.8 (0.5 to 1.1 ) | -0.6 (-0.9 to -0.4 ) | 357 (219 to 554 ) | 55.9 (34.4 to 86.6 ) | 896 (607 to 1286 ) | 45.2 (30.8 to 64.5 ) | -0.6 (-0.9 to -0.4 ) |
| **Panama** | 6 (5 to 7 ) | 0.6 (0.6 to 0.7 ) | 8 (7 to 10 ) | 0.5 (0.4 to 0.6 ) | -0.6 (-1.2 to 0.0 ) | 5 (5 to 6 ) | 0.6 (0.5 to 0.7 ) | 7 (6 to 9 ) | 0.4 (0.3 to 0.5 ) | -0.8 (-1.3 to -0.2 ) | 310 (274 to 352 ) | 33.7 (29.7 to 38.2 ) | 414 (330 to 513 ) | 25.5 (20.3 to 31.6 ) | -0.8 (-1.4 to -0.2 ) |
| **Papua New Guinea** | 11 (5 to 20 ) | 0.7 (0.4 to 1.4 ) | 35 (19 to 60 ) | 0.9 (0.5 to 1.5 ) | 0.5 (0.4 to 0.7 ) | 10 (5 to 19 ) | 0.7 (0.3 to 1.3 ) | 32 (17 to 55 ) | 0.8 (0.4 to 1.4 ) | 0.5 (0.4 to 0.6 ) | 596 (294 to 1126 ) | 39.7 (20.0 to 74.3 ) | 1919 (1034 to 3313 ) | 46.7 (25.1 to 80.6 ) | 0.5 (0.4 to 0.7 ) |
| **Paraguay** | 6 (5 to 8 ) | 0.4 (0.3 to 0.6 ) | 15 (10 to 22 ) | 0.5 (0.3 to 0.7 ) | 0.6 (0.0 to 1.1 ) | 6 (4 to 7 ) | 0.4 (0.3 to 0.5 ) | 14 (9 to 20 ) | 0.4 (0.3 to 0.7 ) | 0.6 (0.3 to 1.0 ) | 326 (236 to 435 ) | 23.0 (16.7 to 30.6 ) | 785 (520 to 1145 ) | 26.0 (17.3 to 38.0 ) | 0.7 (0.3 to 1.0 ) |
| **Peru** | 89 (66 to 120 ) | 1.1 (0.8 to 1.5 ) | 122 (82 to 173 ) | 0.8 (0.6 to 1.2 ) | -1.0 (-2.5 to 0.5 ) | 82 (61 to 109 ) | 1.0 (0.8 to 1.4 ) | 103 (70 to 146 ) | 0.7 (0.5 to 1.0 ) | -1.3 (-2.7 to 0.2 ) | 4929 (3660 to 6549 ) | 61.4 (45.6 to 81.5 ) | 6050 (4084 to 8552 ) | 40.6 (27.4 to 57.4 ) | -1.3 (-2.7 to 0.2 ) |
| **Philippines** | 274 (242 to 307 ) | 1.2 (1.1 to 1.4 ) | 495 (412 to 578 ) | 1.1 (0.9 to 1.3 ) | -0.4 (-0.6 to -0.2 ) | 251 (222 to 281 ) | 1.1 (1.0 to 1.2 ) | 447 (373 to 523 ) | 1.0 (0.8 to 1.2 ) | -0.4 (-0.6 to -0.2 ) | 14707 (12992 to 16451 ) | 64.3 (56.7 to 72.0 ) | 26188 (21830 to 30673 ) | 57.9 (48.2 to 67.8 ) | -0.4 (-0.6 to -0.2 ) |
| **Poland** | 302 (288 to 317 ) | 1.8 (1.7 to 1.8 ) | 111 (100 to 122 ) | 0.7 (0.6 to 0.8 ) | -2.8 (-3.1 to -2.5 ) | 268 (256 to 281 ) | 1.6 (1.5 to 1.6 ) | 94 (86 to 104 ) | 0.6 (0.6 to 0.7 ) | -2.9 (-3.5 to -2.4 ) | 14809 (14139 to 15550 ) | 87.2 (83.2 to 91.6 ) | 5271 (4782 to 5781 ) | 34.6 (31.4 to 37.9 ) | -2.9 (-3.4 to -2.4 ) |
| **Portugal** | 45 (37 to 55 ) | 1.2 (1.0 to 1.5 ) | 21 (17 to 27 ) | 0.6 (0.5 to 0.8 ) | -2.0 (-3.4 to -0.6 ) | 39 (32 to 48 ) | 1.0 (0.9 to 1.3 ) | 16 (13 to 20 ) | 0.5 (0.4 to 0.6 ) | -2.5 (-3.3 to -1.7 ) | 2194 (1785 to 2707 ) | 58.8 (47.8 to 72.5 ) | 892 (712 to 1119 ) | 26.1 (20.8 to 32.7 ) | -2.5 (-3.3 to -1.7 ) |
| **Puerto Rico** | 14 (11 to 17 ) | 1.0 (0.8 to 1.2 ) | 8 (6 to 10 ) | 0.7 (0.5 to 1.0 ) | -1.3 (-2.1 to -0.4 ) | 11 (9 to 14 ) | 0.8 (0.7 to 1.0 ) | 5 (4 to 7 ) | 0.5 (0.4 to 0.6 ) | -1.9 (-3.1 to -0.6 ) | 644 (523 to 786 ) | 47.1 (38.3 to 57.5 ) | 295 (217 to 395 ) | 27.4 (20.2 to 36.7 ) | -1.9 (-3.1 to -0.7 ) |
| **Qatar** | 2 (1 to 3 ) | 0.7 (0.5 to 1.1 ) | 9 (5 to 13 ) | 0.4 (0.3 to 0.6 ) | -1.9 (-2.9 to -0.8 ) | 2 (1 to 3 ) | 0.7 (0.4 to 1.0 ) | 8 (5 to 11 ) | 0.4 (0.2 to 0.5 ) | -2.0 (-3.0 to -0.9 ) | 103 (66 to 154 ) | 38.6 (24.8 to 57.7 ) | 428 (275 to 653 ) | 20.9 (13.6 to 31.7 ) | -2.0 (-2.6 to -1.4 ) |
| **Republic of Korea** | 318 (245 to 401 ) | 1.6 (1.2 to 2.0 ) | 169 (123 to 232 ) | 0.9 (0.7 to 1.2 ) | -1.8 (-2.2 to -1.3 ) | 272 (210 to 344 ) | 1.4 (1.1 to 1.7 ) | 91 (69 to 122 ) | 0.5 (0.4 to 0.7 ) | -3.2 (-3.5 to -2.9 ) | 15766 (12145 to 19917 ) | 78.1 (60.3 to 98.7 ) | 5162 (3856 to 6911 ) | 27.8 (20.7 to 37.3 ) | -3.2 (-3.5 to -3.0 ) |
| **Republic of Moldova** | 29 (25 to 33 ) | 1.5 (1.3 to 1.7 ) | 9 (7 to 11 ) | 0.5 (0.4 to 0.7 ) | -3.4 (-4.1 to -2.6 ) | 25 (22 to 29 ) | 1.3 (1.1 to 1.5 ) | 7 (6 to 8 ) | 0.4 (0.4 to 0.5 ) | -3.7 (-4.4 to -3.0 ) | 1414 (1230 to 1606 ) | 74.0 (64.4 to 84.0 ) | 387 (322 to 461 ) | 24.3 (20.3 to 28.8 ) | -3.7 (-4.4 to -3.0 ) |
| **Romania** | 183 (152 to 221 ) | 2.0 (1.7 to 2.4 ) | 72 (58 to 89 ) | 1.1 (0.9 to 1.4 ) | -1.7 (-2.4 to -1.1 ) | 161 (135 to 193 ) | 1.8 (1.5 to 2.1 ) | 59 (48 to 73 ) | 0.9 (0.8 to 1.2 ) | -2.0 (-2.6 to -1.3 ) | 8972 (7482 to 10761 ) | 98.3 (82.0 to 117.9 ) | 3298 (2645 to 4055 ) | 52.7 (42.1 to 65.0 ) | -1.9 (-2.5 to -1.3 ) |
| **Russian Federation** | 1086 (1048 to 1126 ) | 1.6 (1.6 to 1.7 ) | 513 (474 to 553 ) | 0.8 (0.8 to 0.9 ) | -2.3 (-3.2 to -1.3 ) | 936 (901 to 971 ) | 1.4 (1.4 to 1.5 ) | 401 (371 to 432 ) | 0.7 (0.6 to 0.7 ) | -2.6 (-3.5 to -1.6 ) | 52412 (50529 to 54396 ) | 80.2 (77.4 to 83.3 ) | 22307 (20613 to 24072 ) | 37.2 (34.4 to 40.1 ) | -2.5 (-3.5 to -1.6 ) |
| **Rwanda** | 9 (5 to 13 ) | 0.4 (0.2 to 0.6 ) | 15 (8 to 25 ) | 0.3 (0.2 to 0.5 ) | -0.8 (-1.0 to -0.6 ) | 8 (5 to 12 ) | 0.3 (0.2 to 0.5 ) | 14 (8 to 23 ) | 0.3 (0.1 to 0.4 ) | -0.8 (-1.0 to -0.6 ) | 469 (288 to 689 ) | 19.9 (12.2 to 29.1 ) | 776 (429 to 1323 ) | 15.1 (8.4 to 25.6 ) | -0.8 (-1.0 to -0.6 ) |
| **Saint Kitts and Nevis** | 0 (0 to 0 ) | 0.7 (0.6 to 0.8 ) | 0 (0 to 0 ) | 0.2 (0.2 to 0.4 ) | -3.2 (-3.8 to -2.5 ) | 0 (0 to 0 ) | 0.6 (0.5 to 0.7 ) | 0 (0 to 0 ) | 0.2 (0.1 to 0.3 ) | -3.4 (-4.0 to -2.7 ) | 5 (4 to 6 ) | 34.3 (28.9 to 40.0 ) | 3 (2 to 4 ) | 11.9 (7.9 to 16.9 ) | -3.3 (-4.0 to -2.6 ) |
| **Saint Lucia** | 0 (0 to 0 ) | 0.8 (0.7 to 0.9 ) | 0 (0 to 1 ) | 0.6 (0.5 to 0.8 ) | -0.8 (-1.2 to -0.3 ) | 0 (0 to 0 ) | 0.7 (0.6 to 0.8 ) | 0 (0 to 0 ) | 0.5 (0.4 to 0.6 ) | -0.9 (-1.4 to -0.5 ) | 18 (16 to 20 ) | 38.4 (33.7 to 43.8 ) | 20 (17 to 25 ) | 29.1 (23.6 to 35.6 ) | -0.9 (-1.3 to -0.5 ) |
| **Saint Vincent and the Grenadines** | 0 (0 to 0 ) | 0.6 (0.5 to 0.6 ) | 0 (0 to 0 ) | 0.5 (0.4 to 0.6 ) | -0.3 (-0.9 to 0.2 ) | 0 (0 to 0 ) | 0.5 (0.4 to 0.6 ) | 0 (0 to 0 ) | 0.4 (0.3 to 0.5 ) | -0.4 (-0.9 to 0.1 ) | 10 (9 to 12 ) | 27.7 (23.7 to 32.2 ) | 10 (8 to 13 ) | 24.8 (19.6 to 30.9 ) | -0.2 (-1.0 to 0.6 ) |
| **Samoa** | 0 (0 to 0 ) | 0.2 (0.1 to 0.4 ) | 0 (0 to 0 ) | 0.3 (0.2 to 0.5 ) | 0.7 (0.6 to 0.8 ) | 0 (0 to 0 ) | 0.2 (0.1 to 0.3 ) | 0 (0 to 0 ) | 0.3 (0.1 to 0.4 ) | 0.6 (0.5 to 0.7 ) | 6 (4 to 10 ) | 12.2 (7.7 to 19.4 ) | 10 (6 to 17 ) | 14.7 (8.4 to 23.6 ) | 0.6 (0.5 to 0.7 ) |
| **San Marino** | 0 (0 to 0 ) | 1.2 (0.9 to 1.7 ) | 0 (0 to 0 ) | 0.8 (0.4 to 1.3 ) | -1.6 (-1.8 to -1.4 ) | 0 (0 to 0 ) | 0.9 (0.6 to 1.2 ) | 0 (0 to 0 ) | 0.5 (0.2 to 0.8 ) | -2.0 (-2.3 to -1.6 ) | 5 (3 to 6 ) | 49.6 (35.3 to 68.4 ) | 3 (1 to 4 ) | 27.4 (14.0 to 45.1 ) | -1.9 (-2.3 to -1.6 ) |
| **Sao Tome and Principe** | 0 (0 to 0 ) | 0.8 (0.5 to 1.2 ) | 1 (0 to 1 ) | 0.9 (0.4 to 1.6 ) | 0.0 (-0.5 to 0.5 ) | 0 (0 to 0 ) | 0.7 (0.5 to 1.1 ) | 1 (0 to 1 ) | 0.8 (0.4 to 1.4 ) | 0.0 (-0.5 to 0.5 ) | 14 (8 to 22 ) | 42.2 (25.5 to 64.4 ) | 38 (19 to 71 ) | 45.1 (23.2 to 84.0 ) | 0.1 (-0.4 to 0.6 ) |
| **Saudi Arabia** | 13 (8 to 20 ) | 0.2 (0.1 to 0.3 ) | 60 (35 to 97 ) | 0.3 (0.2 to 0.4 ) | 0.5 (0.2 to 0.9 ) | 12 (7 to 18 ) | 0.2 (0.1 to 0.3 ) | 53 (31 to 88 ) | 0.2 (0.1 to 0.4 ) | 0.4 (0.1 to 0.8 ) | 665 (406 to 1033 ) | 11.4 (7.0 to 17.7 ) | 2978 (1755 to 4934 ) | 13.0 (7.7 to 21.6 ) | 0.4 (0.1 to 0.8 ) |
| **Senegal** | 6 (4 to 10 ) | 0.3 (0.2 to 0.5 ) | 18 (11 to 30 ) | 0.3 (0.2 to 0.5 ) | 0.6 (-0.2 to 1.4 ) | 6 (4 to 9 ) | 0.3 (0.2 to 0.4 ) | 17 (10 to 27 ) | 0.3 (0.2 to 0.5 ) | 0.5 (-0.2 to 1.3 ) | 344 (218 to 540 ) | 15.2 (9.7 to 23.9 ) | 976 (587 to 1581 ) | 17.6 (10.6 to 28.5 ) | 0.6 (-0.2 to 1.4 ) |
| **Serbia** | 75 (50 to 110 ) | 2.0 (1.3 to 2.9 ) | 36 (24 to 51 ) | 1.0 (0.7 to 1.5 ) | -2.0 (-2.3 to -1.6 ) | 65 (43 to 95 ) | 1.7 (1.1 to 2.5 ) | 28 (19 to 39 ) | 0.8 (0.5 to 1.1 ) | -2.3 (-2.6 to -1.9 ) | 3600 (2399 to 5310 ) | 94.7 (63.1 to 139.7 ) | 1549 (1043 to 2191 ) | 45.2 (30.4 to 64.0 ) | -2.3 (-2.6 to -1.9 ) |
| **Seychelles** | 0 (0 to 0 ) | 1.1 (0.7 to 1.5 ) | 0 (0 to 0 ) | 0.7 (0.5 to 1.0 ) | -1.1 (-1.8 to -0.3 ) | 0 (0 to 0 ) | 1.0 (0.7 to 1.4 ) | 0 (0 to 0 ) | 0.6 (0.4 to 0.9 ) | -1.2 (-1.9 to -0.4 ) | 15 (11 to 21 ) | 54.9 (38.4 to 77.6 ) | 15 (10 to 21 ) | 34.9 (23.2 to 50.0 ) | -1.1 (-2.1 to 0.0 ) |
| **Sierra Leone** | 3 (2 to 5 ) | 0.2 (0.1 to 0.3 ) | 9 (5 to 15 ) | 0.3 (0.2 to 0.5 ) | 1.0 (0.8 to 1.3 ) | 3 (2 to 4 ) | 0.2 (0.1 to 0.3 ) | 8 (5 to 14 ) | 0.3 (0.2 to 0.4 ) | 1.0 (0.7 to 1.3 ) | 154 (92 to 252 ) | 11.0 (6.6 to 17.8 ) | 482 (272 to 807 ) | 15.1 (8.6 to 25.2 ) | 1.0 (0.8 to 1.3 ) |
| **Singapore** | 16 (13 to 19 ) | 1.0 (0.8 to 1.2 ) | 17 (13 to 22 ) | 0.7 (0.5 to 0.9 ) | -1.4 (-2.4 to -0.3 ) | 13 (10 to 15 ) | 0.8 (0.7 to 1.0 ) | 9 (7 to 12 ) | 0.4 (0.3 to 0.5 ) | -2.5 (-3.3 to -1.7 ) | 711 (584 to 864 ) | 46.3 (38.1 to 56.3 ) | 528 (410 to 673 ) | 21.9 (17.1 to 27.8 ) | -2.4 (-3.2 to -1.6 ) |
| **Slovakia** | 33 (23 to 45 ) | 1.5 (1.0 to 2.0 ) | 18 (12 to 25 ) | 0.8 (0.6 to 1.2 ) | -1.8 (-2.1 to -1.5 ) | 27 (19 to 37 ) | 1.2 (0.8 to 1.6 ) | 13 (9 to 18 ) | 0.6 (0.4 to 0.9 ) | -2.1 (-2.4 to -1.8 ) | 1503 (1034 to 2052 ) | 66.4 (45.6 to 90.9 ) | 732 (498 to 1031 ) | 34.6 (23.4 to 49.1 ) | -2.1 (-2.3 to -1.8 ) |
| **Slovenia** | 12 (10 to 15 ) | 1.4 (1.2 to 1.7 ) | 6 (4 to 7 ) | 0.8 (0.6 to 1.0 ) | -2.2 (-3.4 to -0.9 ) | 10 (8 to 12 ) | 1.1 (0.9 to 1.4 ) | 4 (3 to 5 ) | 0.5 (0.4 to 0.6 ) | -2.7 (-3.6 to -1.7 ) | 541 (445 to 651 ) | 63.6 (52.3 to 76.7 ) | 204 (162 to 255 ) | 29.3 (23.2 to 36.7 ) | -2.6 (-3.6 to -1.6 ) |
| **Solomon Islands** | 2 (1 to 3 ) | 1.5 (0.8 to 2.6 ) | 5 (3 to 7 ) | 2.0 (1.3 to 2.9 ) | 0.9 (0.7 to 1.0 ) | 1 (1 to 3 ) | 1.4 (0.7 to 2.4 ) | 5 (3 to 7 ) | 1.8 (1.2 to 2.7 ) | 0.9 (0.7 to 1.0 ) | 89 (44 to 152 ) | 82.1 (40.6 to 140.4 ) | 268 (170 to 396 ) | 106.0 (67.5 to 156.3 ) | 0.8 (0.7 to 1.0 ) |
| **Somalia** | 5 (3 to 10 ) | 0.2 (0.1 to 0.4 ) | 14 (7 to 30 ) | 0.2 (0.1 to 0.5 ) | 0.2 (0.1 to 0.4 ) | 5 (3 to 9 ) | 0.2 (0.1 to 0.3 ) | 13 (6 to 27 ) | 0.2 (0.1 to 0.4 ) | 0.2 (0.1 to 0.4 ) | 267 (147 to 494 ) | 10.3 (5.7 to 19.2 ) | 725 (352 to 1519 ) | 10.9 (5.2 to 22.8 ) | 0.2 (0.0 to 0.4 ) |
| **South Africa** | 197 (163 to 235 ) | 1.5 (1.2 to 1.8 ) | 195 (162 to 241 ) | 0.7 (0.6 to 0.9 ) | -2.2 (-3.2 to -1.2 ) | 177 (146 to 211 ) | 1.3 (1.1 to 1.6 ) | 172 (143 to 212 ) | 0.7 (0.5 to 0.8 ) | -2.2 (-3.2 to -1.2 ) | 9942 (8227 to 11851 ) | 75.1 (62.0 to 89.5 ) | 9624 (7995 to 11881 ) | 36.8 (30.6 to 45.5 ) | -2.2 (-3.2 to -1.2 ) |
| **South Sudan** | 4 (2 to 6 ) | 0.2 (0.1 to 0.4 ) | 8 (5 to 13 ) | 0.3 (0.2 to 0.4 ) | 0.8 (0.3 to 1.3 ) | 4 (2 to 6 ) | 0.2 (0.1 to 0.3 ) | 7 (4 to 12 ) | 0.3 (0.2 to 0.4 ) | 0.8 (0.3 to 1.3 ) | 207 (111 to 336 ) | 11.5 (6.2 to 18.5 ) | 429 (258 to 674 ) | 14.3 (8.6 to 22.4 ) | 0.8 (0.3 to 1.3 ) |
| **Spain** | 311 (256 to 375 ) | 2.2 (1.8 to 2.7 ) | 107 (87 to 131 ) | 0.7 (0.6 to 0.9 ) | -3.7 (-4.3 to -3.2 ) | 241 (200 to 289 ) | 1.7 (1.4 to 2.1 ) | 67 (55 to 81 ) | 0.4 (0.4 to 0.5 ) | -4.6 (-5.0 to -4.1 ) | 13650 (11364 to 16399 ) | 96.5 (80.4 to 116.0 ) | 3731 (3080 to 4532 ) | 25.4 (20.9 to 30.9 ) | -4.5 (-5.0 to -4.1 ) |
| **Sri Lanka** | 42 (29 to 60 ) | 0.6 (0.4 to 0.8 ) | 39 (23 to 60 ) | 0.5 (0.3 to 0.7 ) | -0.7 (-1.5 to 0.1 ) | 38 (27 to 55 ) | 0.5 (0.4 to 0.8 ) | 33 (20 to 50 ) | 0.4 (0.2 to 0.6 ) | -0.9 (-1.8 to -0.1 ) | 2253 (1568 to 3215 ) | 31.6 (22.0 to 45.1 ) | 1895 (1170 to 2879 ) | 23.1 (14.2 to 35.1 ) | -1.0 (-1.9 to -0.1 ) |
| **Sudan** | 35 (20 to 56 ) | 0.5 (0.3 to 0.9 ) | 104 (56 to 169 ) | 0.6 (0.3 to 1.0 ) | 0.5 (0.4 to 0.6 ) | 32 (19 to 52 ) | 0.5 (0.3 to 0.8 ) | 96 (52 to 158 ) | 0.6 (0.3 to 1.0 ) | 0.5 (0.4 to 0.6 ) | 1896 (1120 to 3094 ) | 28.9 (17.1 to 47.0 ) | 5687 (3081 to 9399 ) | 33.8 (18.3 to 55.8 ) | 0.5 (0.4 to 0.6 ) |
| **Suriname** | 1 (1 to 2 ) | 0.8 (0.6 to 1.2 ) | 2 (1 to 2 ) | 0.8 (0.5 to 1.1 ) | -0.1 (-1.8 to 1.6 ) | 1 (1 to 1 ) | 0.7 (0.5 to 1.0 ) | 2 (1 to 2 ) | 0.7 (0.5 to 1.0 ) | -0.2 (-1.9 to 1.5 ) | 57 (38 to 79 ) | 42.4 (28.1 to 58.6 ) | 87 (58 to 124 ) | 40.1 (26.6 to 56.9 ) | -0.2 (-2.0 to 1.6 ) |
| **Sweden** | 19 (16 to 23 ) | 0.6 (0.5 to 0.7 ) | 16 (13 to 21 ) | 0.5 (0.4 to 0.6 ) | -1.0 (-1.5 to -0.5 ) | 15 (13 to 18 ) | 0.5 (0.4 to 0.6 ) | 11 (9 to 14 ) | 0.3 (0.2 to 0.4 ) | -1.6 (-2.1 to -1.0 ) | 859 (711 to 1030 ) | 27.5 (22.7 to 32.9 ) | 624 (497 to 794 ) | 17.4 (13.8 to 22.1 ) | -1.4 (-1.9 to -1.0 ) |
| **Switzerland** | 33 (27 to 39 ) | 1.1 (1.0 to 1.4 ) | 25 (20 to 31 ) | 0.8 (0.6 to 1.0 ) | -1.3 (-2.1 to -0.4 ) | 22 (18 to 26 ) | 0.8 (0.6 to 0.9 ) | 14 (11 to 17 ) | 0.4 (0.3 to 0.5 ) | -1.9 (-2.9 to -0.9 ) | 1224 (1016 to 1448 ) | 43.3 (36.0 to 51.3 ) | 779 (626 to 947 ) | 23.9 (19.2 to 29.2 ) | -1.9 (-2.8 to -0.9 ) |
| **Syrian Arab Republic** | 48 (34 to 66 ) | 1.2 (0.9 to 1.7 ) | 38 (25 to 54 ) | 0.8 (0.5 to 1.2 ) | -1.3 (-1.6 to -0.9 ) | 45 (32 to 61 ) | 1.1 (0.8 to 1.5 ) | 35 (23 to 49 ) | 0.7 (0.5 to 1.1 ) | -1.3 (-1.7 to -0.9 ) | 2702 (1901 to 3640 ) | 66.3 (46.5 to 89.7 ) | 2071 (1371 to 2937 ) | 44.0 (29.1 to 62.8 ) | -1.3 (-1.6 to -1.1 ) |
| **Taiwan (Province of China)** | 145 (126 to 167 ) | 1.5 (1.3 to 1.8 ) | 110 (90 to 133 ) | 1.2 (1.0 to 1.5 ) | -0.7 (-1.0 to -0.4 ) | 122 (106 to 140 ) | 1.3 (1.1 to 1.5 ) | 81 (66 to 97 ) | 0.9 (0.7 to 1.1 ) | -1.2 (-1.5 to -0.9 ) | 6997 (6077 to 8026 ) | 74.2 (64.5 to 85.2 ) | 4522 (3719 to 5462 ) | 51.0 (41.9 to 61.8 ) | -1.2 (-1.5 to -0.9 ) |
| **Tajikistan** | 24 (17 to 34 ) | 1.4 (1.0 to 1.9 ) | 29 (17 to 48 ) | 0.7 (0.4 to 1.2 ) | -2.2 (-2.7 to -1.6 ) | 22 (15 to 31 ) | 1.2 (0.9 to 1.7 ) | 26 (15 to 44 ) | 0.6 (0.4 to 1.1 ) | -2.2 (-2.4 to -1.9 ) | 1302 (903 to 1827 ) | 71.9 (49.9 to 100.7 ) | 1533 (888 to 2598 ) | 37.1 (21.6 to 62.8 ) | -2.1 (-2.4 to -1.9 ) |
| **Thailand** | 341 (238 to 466 ) | 1.4 (1.0 to 2.0 ) | 528 (368 to 738 ) | 2.2 (1.6 to 3.1 ) | 1.2 (0.6 to 1.9 ) | 309 (221 to 426 ) | 1.3 (0.9 to 1.8 ) | 441 (306 to 611 ) | 1.9 (1.3 to 2.6 ) | 1.0 (0.3 to 1.7 ) | 18015 (12816 to 24808 ) | 74.5 (53.1 to 102.5 ) | 25152 (17447 to 34888 ) | 108.6 (75.3 to 150.7 ) | 1.0 (0.3 to 1.7 ) |
| **Timor-Leste** | 2 (1 to 3 ) | 0.6 (0.3 to 0.9 ) | 3 (2 to 5 ) | 0.7 (0.4 to 1.0 ) | 0.3 (-0.4 to 1.0 ) | 2 (1 to 2 ) | 0.5 (0.3 to 0.8 ) | 3 (2 to 4 ) | 0.6 (0.4 to 0.9 ) | 0.3 (-0.4 to 1.0 ) | 91 (53 to 145 ) | 31.3 (18.3 to 49.5 ) | 171 (106 to 267 ) | 35.2 (21.9 to 55.0 ) | 0.3 (-0.5 to 1.1 ) |
| **Togo** | 3 (2 to 5 ) | 0.3 (0.2 to 0.4 ) | 10 (5 to 17 ) | 0.3 (0.2 to 0.6 ) | 0.7 (0.4 to 0.9 ) | 3 (2 to 4 ) | 0.2 (0.1 to 0.4 ) | 9 (5 to 15 ) | 0.3 (0.2 to 0.5 ) | 0.7 (0.4 to 0.9 ) | 158 (95 to 244 ) | 14.1 (8.5 to 21.6 ) | 528 (277 to 891 ) | 17.3 (9.1 to 29.2 ) | 0.7 (0.4 to 0.9 ) |
| **Tokelau** | 0 (0 to 0 ) | 1.0 (0.6 to 1.5 ) | 0 (0 to 0 ) | 1.5 (1.0 to 2.3 ) | 1.7 (1.3 to 2.1 ) | 0 (0 to 0 ) | 0.9 (0.5 to 1.4 ) | 0 (0 to 0 ) | 1.4 (0.9 to 2.0 ) | 1.6 (1.2 to 2.0 ) | 0 (0 to 0 ) | 52.5 (31.2 to 83.8 ) | 0 (0 to 1 ) | 81.5 (53.9 to 120.5 ) | 1.7 (1.3 to 2.1 ) |
| **Tonga** | 0 (0 to 0 ) | 1.0 (0.7 to 1.5 ) | 0 (0 to 1 ) | 1.3 (0.8 to 2.1 ) | 0.8 (0.7 to 1.0 ) | 0 (0 to 0 ) | 0.9 (0.6 to 1.3 ) | 0 (0 to 1 ) | 1.2 (0.7 to 1.9 ) | 0.8 (0.6 to 1.0 ) | 17 (11 to 24 ) | 53.9 (35.6 to 77.8 ) | 25 (15 to 40 ) | 69.3 (41.3 to 110.6 ) | 0.8 (0.6 to 1.0 ) |
| **Trinidad and Tobago** | 3 (3 to 3 ) | 0.6 (0.6 to 0.7 ) | 4 (3 to 5 ) | 0.6 (0.5 to 0.8 ) | 0.3 (-1.1 to 1.6 ) | 3 (2 to 3 ) | 0.5 (0.5 to 0.6 ) | 3 (2 to 4 ) | 0.5 (0.4 to 0.7 ) | 0.1 (-1.3 to 1.4 ) | 146 (128 to 165 ) | 30.9 (27.3 to 35.0 ) | 168 (124 to 221 ) | 28.9 (21.5 to 37.9 ) | 0.1 (-1.2 to 1.4 ) |
| **Tunisia** | 16 (10 to 23 ) | 0.6 (0.4 to 0.8 ) | 30 (19 to 46 ) | 0.6 (0.4 to 0.9 ) | 0.3 (0.1 to 0.4 ) | 14 (9 to 22 ) | 0.5 (0.3 to 0.7 ) | 27 (17 to 41 ) | 0.5 (0.3 to 0.8 ) | 0.2 (0.1 to 0.4 ) | 831 (545 to 1239 ) | 28.6 (18.7 to 42.5 ) | 1518 (951 to 2307 ) | 30.6 (19.1 to 46.5 ) | 0.2 (0.1 to 0.3 ) |
| **Turkey** | 555 (356 to 814 ) | 2.6 (1.7 to 3.8 ) | 441 (306 to 610 ) | 1.3 (0.9 to 1.8 ) | -2.2 (-2.7 to -1.7 ) | 513 (328 to 743 ) | 2.4 (1.5 to 3.5 ) | 398 (274 to 553 ) | 1.2 (0.8 to 1.7 ) | -2.3 (-2.7 to -1.8 ) | 30101 (19291 to 43576 ) | 139.2 (89.1 to 201.4 ) | 23078 (15899 to 32072 ) | 69.5 (47.9 to 96.6 ) | -2.2 (-2.6 to -1.8 ) |
| **Turkmenistan** | 16 (14 to 18 ) | 1.2 (1.1 to 1.4 ) | 17 (13 to 22 ) | 0.8 (0.6 to 1.0 ) | -1.3 (-2.6 to 0.1 ) | 15 (13 to 17 ) | 1.1 (1.0 to 1.2 ) | 15 (11 to 19 ) | 0.7 (0.5 to 0.9 ) | -1.3 (-2.6 to 0.0 ) | 877 (769 to 984 ) | 64.2 (56.1 to 72.1 ) | 867 (669 to 1135 ) | 41.6 (32.2 to 54.5 ) | -1.3 (-2.5 to 0.0 ) |
| **Tuvalu** | 0 (0 to 0 ) | 1.1 (0.7 to 1.8 ) | 0 (0 to 0 ) | 1.4 (0.9 to 2.2 ) | 0.8 (0.7 to 0.9 ) | 0 (0 to 0 ) | 1.0 (0.6 to 1.6 ) | 0 (0 to 0 ) | 1.3 (0.8 to 2.0 ) | 0.8 (0.7 to 0.8 ) | 2 (1 to 3 ) | 59.6 (35.5 to 97.3 ) | 4 (2 to 6 ) | 75.1 (44.7 to 118.3 ) | 0.8 (0.7 to 0.8 ) |
| **Uganda** | 9 (6 to 14 ) | 0.2 (0.1 to 0.3 ) | 42 (24 to 65 ) | 0.3 (0.2 to 0.5 ) | 1.6 (1.3 to 1.9 ) | 8 (5 to 13 ) | 0.2 (0.1 to 0.3 ) | 38 (22 to 60 ) | 0.3 (0.2 to 0.4 ) | 1.6 (1.3 to 1.9 ) | 485 (306 to 741 ) | 9.8 (6.3 to 14.8 ) | 2230 (1290 to 3526 ) | 16.1 (9.5 to 25.1 ) | 1.6 (1.3 to 1.9 ) |
| **Ukraine** | 515 (431 to 611 ) | 2.5 (2.1 to 2.9 ) | 183 (123 to 258 ) | 1.0 (0.7 to 1.4 ) | -2.9 (-4.0 to -1.8 ) | 407 (342 to 480 ) | 2.0 (1.7 to 2.3 ) | 136 (93 to 189 ) | 0.8 (0.5 to 1.0 ) | -3.0 (-4.3 to -1.8 ) | 23073 (19393 to 27192 ) | 112.0 (94.2 to 132.1 ) | 7589 (5183 to 10548 ) | 42.8 (29.3 to 59.3 ) | -3.0 (-4.3 to -1.8 ) |
| **United Arab Emirates** | 8 (5 to 12 ) | 0.7 (0.5 to 1.2 ) | 26 (15 to 40 ) | 0.5 (0.3 to 0.7 ) | -1.2 (-2.4 to 0.0 ) | 7 (4 to 11 ) | 0.7 (0.4 to 1.1 ) | 23 (14 to 36 ) | 0.5 (0.3 to 0.7 ) | -1.2 (-2.3 to 0.0 ) | 428 (260 to 664 ) | 40.9 (24.9 to 63.5 ) | 1294 (784 to 2036 ) | 27.5 (17.3 to 42.0 ) | -1.0 (-2.2 to 0.2 ) |
| **United Kingdom** | 204 (198 to 210 ) | 1.0 (0.9 to 1.0 ) | 173 (166 to 180 ) | 0.7 (0.7 to 0.7 ) | -1.2 (-2.1 to -0.3 ) | 161 (157 to 166 ) | 0.8 (0.7 to 0.8 ) | 117 (113 to 121 ) | 0.5 (0.5 to 0.5 ) | -1.7 (-2.6 to -0.8 ) | 9007 (8781 to 9269 ) | 42.7 (41.7 to 44.0 ) | 6519 (6300 to 6751 ) | 26.8 (25.8 to 27.7 ) | -1.7 (-2.6 to -0.8 ) |
| **United Republic of Tanzania** | 20 (13 to 31 ) | 0.3 (0.2 to 0.4 ) | 60 (37 to 92 ) | 0.3 (0.2 to 0.5 ) | 0.5 (0.4 to 0.6 ) | 18 (12 to 28 ) | 0.2 (0.2 to 0.4 ) | 54 (34 to 84 ) | 0.3 (0.2 to 0.4 ) | 0.5 (0.4 to 0.6 ) | 1058 (684 to 1646 ) | 13.6 (8.9 to 21.1 ) | 3139 (1946 to 4858 ) | 15.8 (9.9 to 24.3 ) | 0.5 (0.4 to 0.6 ) |
| **United States of America** | 0 (0 to 1 ) | 1.5 (1.4 to 1.5 ) | 748 (715 to 783 ) | 0.6 (0.6 to 0.7 ) | -2.7 (-2.9 to -2.5 ) | 0 (0 to 1 ) | 1.1 (1.1 to 1.2 ) | 506 (485 to 529 ) | 0.4 (0.4 to 0.4 ) | -3.0 (-3.2 to -2.8 ) | 21 (15 to 29 ) | 62.5 (60.5 to 64.5 ) | 28688 (27451 to 29998 ) | 24.2 (23.2 to 25.3 ) | -2.9 (-3.1 to -2.7 ) |
| **United States Virgin Islands** | 1678 (1623 to 1734 ) | 1.1 (0.7 to 1.5 ) | 0 (0 to 1 ) | 1.4 (0.9 to 2.0 ) | 1.0 (0.8 to 1.3 ) | 1253 (1214 to 1293 ) | 0.9 (0.6 to 1.3 ) | 0 (0 to 0 ) | 1.1 (0.7 to 1.6 ) | 0.9 (0.7 to 1.1 ) | 69801 (67634 to 72034 ) | 52.0 (36.6 to 72.1 ) | 16 (10 to 24 ) | 63.2 (41.3 to 95.1 ) | 1.0 (0.8 to 1.2 ) |
| **Uruguay** | 22 (17 to 27 ) | 2.0 (1.6 to 2.4 ) | 19 (15 to 24 ) | 1.5 (1.2 to 2.0 ) | -0.6 (-1.0 to -0.2 ) | 19 (15 to 23 ) | 1.7 (1.4 to 2.1 ) | 16 (12 to 20 ) | 1.3 (1.0 to 1.6 ) | -0.8 (-1.2 to -0.4 ) | 1069 (849 to 1324 ) | 96.0 (76.2 to 118.8 ) | 890 (688 to 1131 ) | 72.3 (56.0 to 91.8 ) | -0.7 (-1.2 to -0.3 ) |
| **Uzbekistan** | 100 (82 to 120 ) | 1.3 (1.1 to 1.6 ) | 100 (78 to 125 ) | 0.7 (0.5 to 0.9 ) | -2.2 (-3.2 to -1.2 ) | 91 (74 to 109 ) | 1.2 (1.0 to 1.4 ) | 89 (70 to 112 ) | 0.6 (0.5 to 0.8 ) | -2.3 (-3.2 to -1.3 ) | 5287 (4315 to 6335 ) | 68.9 (56.3 to 82.5 ) | 5158 (4020 to 6504 ) | 35.5 (27.6 to 44.8 ) | -2.2 (-3.2 to -1.2 ) |
| **Vanuatu** | 0 (0 to 1 ) | 0.8 (0.4 to 1.6 ) | 1 (1 to 2 ) | 1.1 (0.6 to 1.9 ) | 0.7 (0.5 to 0.9 ) | 0 (0 to 1 ) | 0.8 (0.4 to 1.5 ) | 1 (1 to 2 ) | 1.0 (0.5 to 1.7 ) | 0.7 (0.5 to 0.9 ) | 25 (12 to 48 ) | 45.9 (22.9 to 89.1 ) | 66 (36 to 117 ) | 57.0 (30.7 to 100.3 ) | 0.7 (0.5 to 0.9 ) |
| **Venezuela (Bolivarian Republic of)** | 84 (75 to 95 ) | 1.2 (1.0 to 1.3 ) | 97 (70 to 130 ) | 1.0 (0.7 to 1.3 ) | -0.6 (-1.2 to 0.1 ) | 76 (68 to 86 ) | 1.0 (0.9 to 1.2 ) | 85 (61 to 114 ) | 0.9 (0.6 to 1.1 ) | -0.6 (-1.3 to 0.0 ) | 4457 (3953 to 4997 ) | 60.5 (53.6 to 67.9 ) | 5005 (3612 to 6689 ) | 51.0 (36.8 to 68.0 ) | -0.6 (-1.2 to 0.0 ) |
| **Viet Nam** | 161 (105 to 231 ) | 0.7 (0.4 to 1.0 ) | 332 (213 to 511 ) | 0.8 (0.5 to 1.2 ) | 0.4 (0.3 to 0.5 ) | 146 (95 to 211 ) | 0.6 (0.4 to 0.9 ) | 282 (183 to 437 ) | 0.6 (0.4 to 1.0 ) | 0.2 (0.2 to 0.3 ) | 8346 (5439 to 12082 ) | 34.2 (22.2 to 49.3 ) | 15832 (10258 to 24471 ) | 36.4 (23.6 to 56.1 ) | 0.2 (0.2 to 0.3 ) |
| **Yemen** | 18 (8 to 34 ) | 0.5 (0.2 to 0.9 ) | 60 (28 to 110 ) | 0.5 (0.2 to 0.9 ) | 0.1 (-0.2 to 0.4 ) | 16 (8 to 32 ) | 0.4 (0.2 to 0.8 ) | 55 (25 to 102 ) | 0.4 (0.2 to 0.8 ) | 0.1 (-0.2 to 0.5 ) | 934 (439 to 1832 ) | 23.7 (11.4 to 46.0 ) | 3193 (1416 to 5925 ) | 25.1 (11.3 to 46.3 ) | 0.1 (-0.2 to 0.5 ) |
| **Zambia** | 7 (5 to 10 ) | 0.3 (0.2 to 0.5 ) | 35 (18 to 70 ) | 0.5 (0.3 to 1.0 ) | 1.4 (1.3 to 1.6 ) | 7 (5 to 10 ) | 0.3 (0.2 to 0.4 ) | 32 (16 to 64 ) | 0.5 (0.2 to 0.9 ) | 1.4 (1.3 to 1.5 ) | 396 (270 to 570 ) | 17.3 (11.8 to 24.7 ) | 1835 (944 to 3728 ) | 26.7 (13.9 to 53.7 ) | 1.4 (1.3 to 1.5 ) |
| **Zimbabwe** | 11 (8 to 15 ) | 0.4 (0.3 to 0.5 ) | 41 (25 to 62 ) | 0.7 (0.5 to 1.1 ) | 2.4 (1.7 to 3.1 ) | 10 (7 to 14 ) | 0.3 (0.2 to 0.5 ) | 37 (23 to 56 ) | 0.7 (0.4 to 1.0 ) | 2.4 (1.7 to 3.1 ) | 566 (391 to 797 ) | 18.3 (12.6 to 25.6 ) | 2107 (1296 to 3221 ) | 37.8 (23.2 to 57.8 ) | 2.4 (1.7 to 3.2 ) |

Rates are reported per 100,000 person-years. Data in parentheses are 95% uncertainty intervals for cases and age-standardized rates of incidence, mortality, and DALYs, and 95% confidence intervals for AAPCs. Abbreviations: AYAs, adolescents and young adults; DALYs, disability-adjusted life-years; ASIR, age-standardized incidence rate; ASMR, age-standardized mortality rate; ASDR, age-standardized DALYs rate; AAPC, average annual percent change; UI, uncertainty interval.
