# Supplementary material for: Persistent burden and health inequalities of lung cancer among adolescents and young adults, 1990-2021
Source: Front Oncol. 2025 Sep 30;15:1624401. doi: 10.3389/fonc.2025.1624401 (PMC12518105; doi:10.3389/fonc.2025.1624401)
Supplement: Supplementary file 7 [file Table3.docx]

**Supplemental table 3. Changes in** **incidence, mortality, and DALYs number according to population-level determinants and causes from 1990 to 2021.**

| **Location** | **Overall difference** **in incidence** | **Change due to Population-level determinants in incidence (% contribute to the total changes)** | | | **Overall difference in** **mortality** | **Change due to Population-level determinants in mortality (% contribute to the total changes)** | | | **Overall difference in** **DALYs** | **Change due to Population-level determinants in DALYs (% contribute to the total changes)** | | |
| --- | --- | --- | --- | --- | --- | --- | --- | --- | --- | --- | --- | --- |
|  |  | **Aging** | **Population** | **Change in incidence rate** |  | **Aging** | **Population** | **Change in mortality rate** |  | **Aging** | **Population** | **Change in DALYs rate** |
| **Global** | 1213.1(4.5%) | 3143.9(11.7%) | 8532(31.8%) | -10463.1(-39.0%) | -687.0(-2.9%) | 2545.0(10.9%) | 7199(30.8%) | -10431.7(-44.7%) | -44848.1(-3.3%) | 134224.6(10.0%) | 411596.4(30.8%) | -590669(-44.2%) |
| **Female** | 1818.8(19.4%) | 1205.3(12.9%) | 3111(33.3%) | -2497.7(-26.7%) | 556.7(6.9%) | 940.7(11.7%) | 2547(31.7%) | -2931.08(-36.5%) | 29397.0(6.3%) | 49560.9(10.7%) | 146001.2(31.6%) | -166165(-36.0%) |
| **Male** | -605.6(-3.4%) | 1893.7(10.8%) | 5444(31.1%) | -7944.4(-45.4%) | -1243.7(-8.1%) | 1564.5(10.2%) | 4674(30.5%) | -7482.9(-48.9%) | -74245.1(-8.4%) | 82576.8(9.4%) | 266853.1(30.5%) | -423675.168(-48.4%) |
| **High SDI** | -2022.3(-38.0%) | 400.2(7.5%) | 78(1.4%) | -2500.8(-47.0%) | -1438.0(-54.1%) | 192.9(7.2%) | 65(2.4%) | -1696.4(-63.9%) | -109781.5(-47.4%) | 15073.8(6.5%) | 3205.4(1.3%) | -128060(-55.3%) |
| **High-middle SDI** | -1443.5(-15.2%) | 1711.3(18.0%) | -243(-2.5%) | -2911.6(-30.6%) | -1669.3(-29.1%) | 899.3(15.7%) | -25(-0.4%) | -2543.6(-44.4%) | -119028.0(-25.1%) | 73092.1(15.4%) | -11518.1(-2.4%) | -180602(-38.0%) |
| **Middle SDI** | 1774.9(18.7%) | 2008.3(21.2%) | 2173(22.9%) | -2406.8(-25.4%) | 456.9(8.4%) | 995.1(18.4%) | 1208(22.3%) | -1746.4(-32.3%) | 34710.2(7.0%) | 88067.2(17.7%) | 108381.3(21.8%) | -161738(-32.6%) |
| **Low-middle SDI** | 2198.7(106.0%) | 269.6(12.9%) | 1726(83.2%) | 202.6(9.7%) | 1054.8(84.9%) | 134.9(10.8%) | 975(78.6%) | -56.0(-4.5%) | 111888.1(100.2%) | 12260.3(10.9%) | 91502.4(82.0%) | 8125(7.2%) |
| **Low SDI** | 712.0(168.8%) | 0.04(0.01%) | 637(151.0%) | 74.8(17.7%) | 358.5(140.8%) | -2.8(-1.1%) | 364(143.0%) | -2.7(-1.0%) | 37708.5(167.4%) | -1.5(-0.007%) | 33935.0(150.6%) | 3774(16.7%) |
| **High-income Asia Pacific** | -445.6(-49.9%) | 98.2(11.0%) | -189(-21.2%) | -354.1(-39.6%) | -283.9(-68.7%) | 40.1(9.7%) | -71(-17.4%) | -252.0(-61.0%) | -24881.4(-65.2%) | 3414.4(8.9%) | -7157.5(-18.7%) | -21138(-55.4%) |
| **High-income North America** | -1017.9(-54.7%) | -18.6(-1.0%) | 114(6.1%) | -1113.9(-59.8%) | -524.4(-64.0%) | -8.4(-1.0%) | 49(5.9%) | -565.0(-69.0%) | -45353.6(-58.5%) | -790.5(-1.0%) | 4646.8(6.0%) | -49209(-63.5%) |
| **Western Europe** | -869.3(-40.5%) | 184.8(8.6%) | -179(-8.3%) | -875.0(-40.7%) | -789.4(-64.1%) | 84.0(6.8%) | -84(-6.8%) | -789.2(-64.1%) | -49974.7(-53.5%) | 6783.1(7.2%) | -7127.7(-7.6%) | -49630(-53.2%) |
| **Australasia** | 8.1(11.0%) | 6.5(8.9%) | 19(26.5%) | -17.9(-24.4%) | -7.2(-27.3%) | 1.7(6.4%) | 6(22.6%) | -15.0(-56.4%) | -326.6(-11.6%) | 210.1(7.5%) | 674.8(24.0%) | -1211(-43.2%) |
| **Andean Latin America** | 60.3(44.5%) | 19.4(14.3%) | 93(68.9%) | -52.4(-38.7%) | 19.0(26.7%) | 7.9(11.2%) | 48(68.6%) | -37.8(-53.1%) | 2480.5(33.3%) | 835.2(11.2%) | 4944.9(66.5%) | -3299(-44.3%) |
| **Tropical Latin America** | 160.4(33.6%) | 97.9(20.5%) | 177(37.1%) | -114.5(-24.0%) | 22.2(8.5%) | 45.1(17.3%) | 91(35.0%) | -114.3(-43.8%) | 6748.6(27.0%) | 4292.4(17.2%) | 9036.1(36.2%) | -6579(-26.3%) |
| **Central Latin America** | 47.5(9.2%) | 71.6(13.9%) | 216(42.2%) | -241.0(-46.9%) | -15.5(-5.4%) | 30.6(10.8%) | 116(41.0%) | -162.3(-57.3%) | 676.4(2.4%) | 3101.6(11.1%) | 11454.0(41.1%) | -13879(-49.8%) |
| **Southern Latin America** | -62.4(-22.8%) | 20.9(7.6%) | 76(27.9%) | -159.6(-58.5%) | -80.9(-45.8%) | 11.2(6.3%) | 45(25.9%) | -138.1(-78.2%) | -3595.6(-26.5%) | 920.1(6.8%) | 3710.7(27.4%) | -8226(-60.8%) |
| **Caribbean** | -0.7(-0.5%) | 20.8(14.9%) | 28(20.5%) | -50.3(-36.0%) | -10.0(-13.3%) | 10.3(13.6%) | 16(21.4%) | -36.6(-48.3%) | -375.7(-5.4%) | 881.9(12.7%) | 1387.26(20.0%) | -2644(-38.2%) |
| **Central Europe** | -590.3(-59.6%) | 78.9(7.9%) | -195(-19.7%) | -473.8(-47.8%) | -466.5(-71.1%) | 49.1(7.4%) | -113(-17.2%) | -402.4(-61.3%) | -29745.6(-62.3%) | 3476.4(7.2%) | -9189.2(-19.2%) | -24032(-50.4%) |
| **Eastern Europe** | -986.4(-56.8%) | 207.9(11.9%) | -315(-18.1%) | -879.0(-50.6%) | -759.0(-66.4%) | 130.4(11.4%) | -188(-16.5%) | -700.8(-61.3%) | -49811.6(-60.8%) | 8664.6(10.5%) | -14392.1(-17.5%) | -44084(-53.8%) |
| **Central Asia** | -193.8(-37.8%) | 67.8(13.2%) | 121(23.6%) | -383.0(-74.7%) | -144.5(-44.7%) | 42.3(13.1%) | 78(24.3%) | -265.5(-82.2%) | -10663.8(-39.6%) | 3031.5(11.2%) | 6283.5(23.3%) | -19979(-74.3%) |
| **North Africa and Middle East** | 733.8(64.7%) | 297.5(26.2%) | 966(85.2%) | -530.2(-46.7%) | 311.4(40.5%) | 188.8(24.5%) | 631(82.1%) | -508.5(-66.1%) | 36962.5(59.8%) | 13247.9(21.4%) | 51847.2(83.9%) | -28132(-45.5%) |
| **South Asia** | 1907.6(127.2%) | 195.1(13.0%) | 1394(93.0%) | 317.7(21.2%) | 904.9(90.5%) | 104.2(10.4%) | 831(83.1%) | -30.2(-3.0%) | 95822.5(119.5%) | 8876.2(11.0%) | 73209.6(91.3%) | 13736(17.1%) |
| **Southeast Asia** | 1352.7(76.6%) | 342.2(19.3%) | 810(45.9%) | 200.2(11.3%) | 626.8(63.6%) | 165.8(16.8%) | 475(48.2%) | -14.1(-1.4%) | 64528.2(68.1%) | 15096.4(15.9%) | 42366.5(44.7%) | 7065(7.4%) |
| **East Asia** | 533.6(4.4%) | 3313.4(27.3%) | -2076(-17.1%) | -703.0(-5.7%) | -339.5(-5.0%) | 1602.0(23.7%) | -981(-14.5%) | -960.1(-14.2%) | -67285.0(-10.7%) | 143515.6(23.0%) | -98670.5(-15.8%) | -112130(-17.9%) |
| **Oceania** | 30.1(158.1%) | 2.0(10.9%) | 23(124.1%) | 4.4(23.0%) | 18.9(139.3%) | 0.8(6.5%) | 16(118.6%) | 1.9(14.1%) | 1640.1(154.6%) | 89.6(8.4%) | 1307.2(123.2%) | 243(22.9%) |
| **Western Sub-Saharan Africa** | 176.6(214.8%) | -0.6(-0.7%) | 149(182.1%) | 27.5(33.5%) | 87.9(182.3%) | -4.1(-8.5%) | 81(169.8%) | 10.1(21.0%) | 9262.1(212.9%) | -38.6(-0.8%) | 7894.7(181.5%) | 1406(32.3%) |
| **Eastern Sub-Saharan Africa** | 233.3(160.0%) | 6.8(4.6%) | 220(150.9%) | 6.3(4.3%) | 110.9(132.7%) | 2.7(3.3%) | 121(145.3%) | -13.3(-15.9%) | 12217.8(158.4%) | 315.2(4.0%) | 11606.1(150.5%) | 296(3.8%) |
| **Central Sub-Saharan Africa** | 95.4(157.5%) | 2.5(4.1%) | 96(159.6%) | -3.7(-6.2%) | 53.4(143.1%) | 1.7(4.6%) | 58(155.9%) | -6.5(-17.4%) | 4993.3(155.8%) | 114.4(3.5%) | 5097.6(159.1%) | -218(-6.8%) |
| **Southern Sub-Saharan Africa** | 40.1(18.7%) | 56.4(26.3%) | 110(51.5%) | -126.5(-59.1%) | 21.8(16.8%) | 33.8(26.1%) | 71(54.8%) | -83.0(-64.1%) | 1833.4(16.9%) | 2644.1(24.4%) | 5526.3(51.1%) | -6337(-58.6%) |

Abbreviations: AYAs, adolescents and young adults; DALYs, disability-adjusted life-years; SDI, socio-demographic index.
